# Review of sample size determination methods for the intraclass correlation coefficient in the one-way analysis of variance model

**DOI:** 10.1177/09622802231224657

**Published:** 2024-02-06

**Authors:** Dipro Mondal, Sophie Vanbelle, Alberto Cassese, Math JJM Candel

**Affiliations:** 1 Faculty of Health Medicine and Life Sciences, Department of Methodology and Statistics, Care and Public Health Research Institute (CAPHRI), Maastricht University, Limburg, The Netherlands; 2 Department of Statistics, Computer Science, Applications “Giuseppe Parenti”, The University of Florence, Italy

**Keywords:** Intrarater reliability, interrater reliability, measurement errors, reproducibility (of results), observer variation

## Abstract

Reliability of measurement instruments providing quantitative outcomes is usually assessed by an intraclass correlation coefficient. When participants are repeatedly measured by a single rater or device, or, are each rated by a different group of raters, the intraclass correlation coefficient is based on a one-way analysis of variance model. When planning a reliability study, it is essential to determine the number of participants and measurements per participant (i.e. number of raters or number of repeated measurements). Three different sample size determination approaches under the one-way analysis of variance model were identified in the literature, all based on a confidence interval for the intraclass correlation coefficient. Although eight different confidence interval methods can be identified, Wald confidence interval with Fisher’s large sample variance approximation remains most commonly used despite its well-known poor statistical properties. Therefore, a first objective of this work is comparing the statistical properties of all identified confidence interval methods—including those overlooked in previous studies. A second objective is developing a general procedure to determine the sample size using all approaches since a closed-form formula is not always available. This procedure is implemented in an R Shiny app. Finally, we provide advice for choosing an appropriate sample size determination method when planning a reliability study.

## 1. Introduction

Reliability is important in many scientific disciplines.^1–3^ All measurement and evaluation processes are subject to measurement error. These errors can have a serious impact on research undermining the conclusions of the study, as well as in daily practice when measurement and evaluation processes are used to make diagnoses or assess the progression of participants, for example. It is therefore essential for measurement instruments to be reliable (i.e. the device/rater is able to distinguish among participants in a population) and valid (i.e. measurements reflect the underlying true values). The reliability of a device/rater is usually evaluated during a reliability study. Generally, a reliability study consists of participants measured repeatedly under similar conditions by the same device/rater (intrarater reliability) or by different devices/raters (interrater reliability). In interrater reliability studies, the set of raters can be the same, or different for every participant. In this article, we focus (1) on intrarater studies where the same number of repeated measurements is made simultaneously on each participant, and the order of the measurements is interchangeable, and, (2) on specific interrater reliability studies where the set of raters is different for every participant, and the same number of raters rates each participant. In the second case the reliability coefficient additionally reflects the differences between raters, next to the measurement error.

When the outcome measurements are quantitative, reliability can be quantified using an intraclass correlation coefficient (ICC). ICC is defined as the correlation between repeated measurements at multiple occasions made by the same rater/device or by different raters/devices on the same participants. It compares the variability of measurements/ratings within participants to the variability of measurements/ratings between participants. Depending on the design of the study, different forms of ICC should be used.^4,5^ This article focuses on the ICC defined in the one-way analysis of variance (ANOVA) model, ICC(1).^4^ When planning a reliability study, determining the minimum number of raters/repetitions and participants is of prime importance. In fact, too many participants may prove to be time-consuming and may also increase the research budget, while too few may adversely impact the precision of the ICC estimate, preventing the drawing of any conclusion on the study. Several approaches to determine sample sizes can be identified in the literature. The aim of this review is two-fold. First, it is to compare the statistical properties of the sample sizes obtained with the approaches in realistic settings. Second, it is to develop a general procedure for sample size determination, since a closed-form formula is not always available for all the approaches.

Existing literature on determining sample size indicates two main approaches, namely, the confidence interval approach^6,8^ and the hypothesis testing approach.^6,8–10^ The confidence interval approach requires defining, around a planned ICC, a target width of the confidence interval that the researcher aims to achieve. A generalization of the width of the confidence interval approach, the assurance probability approach,^6^ is based on testing whether the width of the confidence interval is less than a pre-specified width with a given assurance probability. The testing approach is based on the power of testing the hypothesis that the ICC is lower or equal to (null hypothesis), or, above (alternative hypothesis) a pre-specified value of the ICC. A common feature of these approaches is that the variance of the ICC estimator needs to be defined. In the literature, two closed-form approximations of the large-sample variance of the ICC estimate are mainly used. These are namely, the Swiger variance,^11^ which is based on the Taylor-series expansion of the ratio of the ANOVA mean squares, and the Fisher variance,^12^ a large-sample approximation obtained by Fisher. We further consider another form of the variance, known as the Zerbe variance,^13^ based on the formulation of the ratio of two independent *F*-statistics. This variance is far less popular and was not included in previous reviews.

Confidence intervals formed around the ICC are mainly based on the Wald method,^6,14^ or on the *F*-statistic, termed the Searle method.^15^ The Wald and the Searle methods can be further applied using a normalization transformation.^12,16^ When comparing the coverage probability of the confidence intervals (confidence intervals based on the Wald method with the Fisher variance and the Searle method), Zou^6^ concluded that the normalized Searle method performs better than the Wald method with the Fisher variance. However, when comparing the coverage probabilities and mean interval widths of confidence intervals obtained with the Wald method (with the Swiger variance), the Searle method, and the normalized Searle method, Donner and Wells^17^ concluded that no method was superior in all situations.

In the context of sample size determination with the confidence interval approach, a closed-form formula was derived by Bonett^7^ for the Wald confidence interval with the Fisher variance and is the most common choice.^18^ While Shieh more recently defined a numerical procedure for the Searle method,^19^ no procedure to determine sample sizes exists for the other methods. Note that, in common statistical software like R,^18^ SAS, and PASS,^20^ the Wald method with the Fisher variance is the only one that is available (see Appendix E). As for the comparison among the methods, Shieh^19^ compared the statistical properties of the Wald method (with the Fisher variance) and the Searle method with respect to the width of the confidence interval approach and the assurance probability approach. To summarize the results, the Searle method and the assurance probability approach, with a 90% assurance probability, showed better coverage than the width of the confidence interval approach.^6^ Furthermore, this was achieved with a somewhat smaller width of the confidence interval. We aim to complete these comparisons by considering all the identified confidence interval methods.

For the testing approach, sample size determination was derived only for the normalized Searle method^18^ and the Searle method (numerically). Only the latter is available in common statistical software.^20^ As for the comparison, Shieh^21^ showed that the approximate sample size formula obtained using the normalized Searle method^8^ under-performs, with respect to the observed power of the hypothesis test, when compared to numerical sample sizes obtained via the Searle method. We extend the work of Shieh,^21^ by comparing the results that can be obtained using all the methods for sample size determination identified in this article.

Several studies have investigated inference procedures for the ICC in this context but are incomplete as these studies do not consider all the confidence interval methods identified. In summary, our contribution is as follows. First, we compare the statistical properties of all identified confidence interval methods. Second, we analytically derive the sample size formulas using the Swiger and the Zerbe variances. Third, we develop a numerical procedure to obtain sample sizes with all identified confidence interval methods under the three sample size approaches. In this numerical procedure, we derive formulas to approximate the assurance probability function and the power function (except for the Searle method for which these formulas were already derived^21^). Additionally, we provide guidelines for end users. We further provide an user-friendly and interactive R Shiny application to obtain sample sizes with all the methods discussed in this article on https://github.com/DiproMondal/sample-size-ICCGithub and the https://dipro.shinyapps.io/sample-size-icc/Shiny server.

The article is organized as follows. Section 2 introduces the methods to estimate ICC, its variance, and confidence interval. Section 3 introduces the simulation setup that is used to evaluate the statistical properties of the confidence interval methods. Section 4 describes different approaches for sample size calculation when the number of raters, k , is fixed. We further propose a general procedure to obtain minimum sample sizes under any approach. Section 5 presents a case study. Finally, Section 6 concludes the article with a summary and a discussion of the results obtained in this article.

## 2. Definition

Consider the scenario in which each participant is measured on a quantitative scale by a different set of raters randomly drawn from a population of raters,^4^ or is measured repeatedly by a measuring device several times under identical conditions. Further assume that the number of raters/repeated measurements per participant is the same, which is a common assumption when planning a reliability study. Let $Y_{ij}$ represent the measurement of participant i (i=1,2,…,n) by rater j (j=1,2,…,k) . This outcome can be described by a one-way ANOVA model, which can be written as

(1) $$Y_{ij}=\mu +s_{i}+\epsilon_{ij}$$

where μ is the grand mean, $s_{i}$ is the effect of participant i , and $\epsilon_{ij}$ is the measurement error for participant i measured by rater j . The total number of observations is denoted by N ( N=kn ). The assumptions of this ANOVA model are that the participant effects $s_{i}$ are identically and normally distributed with mean 0 and variance $\sigma_{s}^{2}$ , the measurement errors $\epsilon_{ij}$ are identically and normally distributed with mean 0 and variance $\sigma_{\epsilon }^{2}$ , and the errors and participant effects are independent. Table 1 shows the variance components of this one-way ANOVA model. The mean squares in Table 1 are $BMS=\frac{k}{n-1}\sum_{i=1}^{n}({\bar{Y}}_{i.}-{\bar{Y}}_{..}{)}^{2}$ and $WMS=\frac{1}{N-n}\sum_{i=1}^{n}\sum_{j=1}^{k}(Y_{ij}-{\bar{Y}}_{i.}{)}^{2}$ , where ${\bar{Y}}_{i.}=\frac{1}{k}\sum_{j=1}^{k}Y_{ij}$ and ${\bar{Y}}_{..}=\frac{1}{N}\sum_{i=1}^{n}\sum_{j=1}^{k}Y_{ij}$ . Using this variance decomposition, the ICC is defined as

(2) $$\rho =\frac{\sigma_{s}^{2}}{\sigma_{s}^{2}+\sigma_{\epsilon }^{2}},\;0\leq \rho \leq 1$$

Note that the value of ρ becomes closer to 1 as the measurement error variance becomes smaller ( $\sigma_{\epsilon }^{2}<<\sigma_{s}^{2}$ ) and ρ becomes closer to 0 as it increases ( $\sigma_{\epsilon }^{2}>>\sigma_{s}^{2}$ ).

**Table 1.** Variance decomposition as for the one-way ANOVA model described by equation (1).

Source of	Degrees of	Mean	Expected
variation	freedom	squares	mean squares
Between participants	n−1	BMS	$\sigma_{\epsilon }^{2}+k\sigma_{s}^{2}$
Within participants	N−n	WMS	$\sigma_{\epsilon }^{2}$

ANOVA: analysis of variance; BMS: between mean squares; WMS: within mean squares.

### 2.1. Estimation of ICC

ICC is usually estimated using the ANOVA^4^ or the maximum-likelihood estimator. The ANOVA estimator is given by

(3) $${\hat{\rho }}_{ANOVA}=\frac{BMS-WMS}{BMS+(k-1)WMS}$$

Since this estimator is negatively biased,^22^ a maximum-likelihood estimator has been suggested^23^:

$${\hat{\rho }}_{ML}=\frac{BMS(n-1)/n-WMS}{BMS(n-1)/n+(k-1)WMS}$$

Comparing the bias of the two estimators, Wang et al.^23^ showed that the bias of ${\hat{\rho }}_{ML}$ is still quite large and decreases only slightly for large samples. For instance, to achieve a bias of ${\hat{\rho }}_{ML}$ not > 10 % , a total of 100 observations (e.g. 20 participants and five raters) are required when expecting ρ=0.5 (the value of ρ at which the bias is maximum). When one expects higher values of ρ , as in the context considered in this article, the bias of ${\hat{\rho }}_{ANOVA}$ is small and the two estimators lead to almost identical estimates. For this reason, the maximum-likelihood estimator is generally not used in the literature. Accordingly, we will only consider the ANOVA estimator in this article. Note that this estimator relies on the assumptions of the ANOVA model (equation (1)). A brief discussion of what happens when these assumptions are violated is given in Section 6.

### 2.2. Large sample variance of the ICC

Here we focus on the three approximated closed-form expressions of the variance of $\hat{\rho }$ available in the literature for large n . Swiger et al.^11^ provided the large sample variance of $\hat{\rho }$ as,

(4) $$var(\hat{\rho }{)}_{S}=\frac{2(N-1)(1-\rho {)}^{2}[1+(k-1)\rho {]}^{2}}{k^{2}(N-n)(n-1)}.$$

Given that k(N−n)=nk(k−1) , as N=kn , this leads to the variance obtained by Fisher^12^ when $\frac{N-1}{k(n-1)}\approx 1$ , which is a reasonable assumption for small k and n≥30 ,^24^

(5) $$var(\hat{\rho }{)}_{F}=\frac{2(1-\rho {)}^{2}[1+(k-1)\rho {]}^{2}}{nk(k-1)}.$$

Note that in equation (5), n is sometimes replaced by n−1 .^25^ Lastly, following Zerbe and Goldgar^13^ and Kaart,^26^ the variance can also be estimated by the ratio of two independent *F*-statistics as,

(6) $$var(\hat{\rho }{)}_{Ze}=\frac{2(1-\rho {)}^{2}[1+(k-1)\rho {]}^{2}(N-n{)}^{2}(N-3)}{k^{2}(n-1)(N-n-2{)}^{2}(N-n-4)}.$$

These three formulas are related by the following inequality, $var(\hat{\rho }{)}_{Ze}>var(\hat{\rho }{)}_{S}>var(\hat{\rho }{)}_{F}$ (see Appendix A for proof).

### 2.3. Confidence interval for the ICC

In the literature, there are four methods to compute the upper ( U ) and lower ( L ) bounds of the confidence interval for ρ , namely the Wald method,^6^ the Searle method,^9,15^ and their normalized versions. Demetrashvili et al.^27^ further suggested two generic methods not considered here because they are not accurate in the balanced one-way random effects model.

#### 2.3.1. Wald confidence interval ( $Wald_{S}$ , $Wald_{F}$ , and $Wald_{Ze}$ )

Based on the central limit theorem, the upper ( U ) and lower ( L ) bounds of the confidence interval for the ICC can be written as,^6,14^

(7) $$U,L=\hat{\rho }\pm z_{1-\alpha /2}\sqrt{var(\hat{\rho })},$$

where $z_{1-\alpha /2}$ is the (1−α/2)×100 percentile of the standard normal distribution. Plugging equations (4) to (6) into (7) as the variance leads to confidence intervals, which we denote as *$Wald_{S}$ *, *$Wald_{F}$ *, and *$Wald_{Ze}$ *, respectively.

The Wald method assumes that the sampling distribution of $\hat{\rho }$ is normally distributed. However, ρ is bounded between 0 and 1, implying a skewed sampling distribution of $\hat{\rho }$ when ρ is close to the boundaries.^28^ Since typically ICC values close to one are of interest in a reliability study, Wald confidence intervals may thus have poor statistical properties in this context.

#### 2.3.2. Searle method ( $F_{\rho }$ )

Under the assumption of normality of the ANOVA model, the ratio of the between-mean squares and within-mean squares (i.e. the *F*-statistic) is distributed as $\frac{1+(k-1)\rho }{1-\rho }F_{\nu_{1},\nu_{2}}$ , where $F_{\nu_{1},\nu_{2}}$ represents an *F*-distribution with $\nu_{1}=n-1$ and $\nu_{2}=n(k-1)$ degrees of freedom. We represent this ratio as

(8) $$F(\hat{\rho })=\frac{BMS}{WMS}=\frac{1+(k-1)\hat{\rho }}{1-\hat{\rho }}.$$

Then, the upper and lower bounds of the confidence interval for ρ are given by Searle^14^ as,

(9) $$U,L=\frac{F(\hat{\rho })/F_{l}-1}{F(\hat{\rho })/F_{l}+k-1},\frac{F(\hat{\rho })/F_{u}-1}{F(\hat{\rho })/F_{u}+k-1}.$$

where $F_{l}$ and $F_{u}$ are the α/2×100 and the (1−α/2)×100 percentile of an *F*-distribution with n−1 and n(k−1) degrees of freedom, respectively. We denote this method as $F_{\rho }$ .

Rather than making a normality assumption on $\hat{\rho }$ , this method makes an assumption of normality on the outcome $Y_{ij}$ . Hence this method has been referred to as being an exact procedure by several authors.^6,17^

#### 2.3.3. Normalized ICC method ( $Z_{S},Z_{F},\mathrm{and}\;Z_{Ze}$ )

The Fisher transformation can be applied to the ICC so that the transformed ICC approximately follows a normal distribution. Applying this transformation to $\hat{\rho }$ leads to

(10) $$Z(\hat{\rho })=\frac{1}{2}ln\frac{1+\hat{\rho }}{1-\hat{\rho }}\dot{∼}N(E(Z(\hat{\rho })),var(Z(\hat{\rho }))),$$

where $E(Z(\hat{\rho }))=\frac{1}{2}ln\frac{1+\rho }{1-\rho }$ , and the variance, $var(Z(\hat{\rho }))$ can be derived applying the Delta method^16^ to one of the variances defined in equations (4) to (6) leading, respectively, to

(11) $$\begin{array}{rl} var(Z(\hat{\rho }){)}_{S} & =\frac{2(N-1)[1+(k-1)\rho {]}^{2}}{k^{2}(1+\rho {)}^{2}(N-n)(n-1)}, \end{array}$$

(12) $$\begin{array}{rl} var(Z(\hat{\rho }){)}_{F} & =\frac{2[1+(k-1)\rho {]}^{2}}{N(1+\rho {)}^{2}(k-1)}, \end{array}$$

(13) $$\begin{array}{rl} var(Z(\hat{\rho }){)}_{Ze} & =\frac{2[1+(k-1)\rho {]}^{2}(N-n{)}^{2}(N-3)}{k^{2}(n-1)(N-n-2{)}^{2}(N-n-4)(1+\rho {)}^{2}}. \end{array}$$

Since $Z(\hat{\rho })$ is approximately normally distributed and defined on the real line, we can then compute the Wald confidence interval for this transformation as

$$U_{Z},L_{Z}=Z(\hat{\rho })\pm z_{1-\alpha /2}\sqrt{var(Z(\hat{\rho }))}.$$

Finally, the confidence interval for ρ is obtained by back-transformation leading to

(14) $$U,L=\frac{exp(2U_{Z})-1}{exp(2U_{Z})+1},\frac{exp(2L_{Z})-1}{exp(2L_{Z})+1}.$$

We refer to the confidence intervals obtained by these methods as *$Z_{S}$ *, *$Z_{F}$ *, and *$Z_{Ze}$ *, respectively.

#### 2.3.4. Normalized Searle method ( $ZF_{\rho }$ )

The *F*-statistic ( $F(\hat{\rho })$ ) can also be normalized by a log-transformation to obtain confidence limits.^6,9,14^ Normalizing $F(\hat{\rho })$ starting from equation (8), we obtain

(15) $$Z(F(\hat{\rho }))=\frac{1}{2}ln\frac{1+(k-1)\hat{\rho }}{1-\hat{\rho }}∼N(E(Z(F(\hat{\rho }))),var(Z(F(\hat{\rho })))),$$

where $E(Z(F(\hat{\rho })))=\frac{1}{2}ln\frac{1+(k-1)\rho }{1-\rho }$ and $var(Z(F(\hat{\rho })))=\frac{1}{2}(\frac{1}{n-1}+\frac{1}{n(k-1)})$ . The confidence interval on this log transformed scale $Z(F(\hat{\rho }))$ is then

$$U_{ZF},L_{ZF}=Z(F(\hat{\rho }))\pm z_{1-\alpha /2}\sqrt{var(Z(F(\hat{\rho })))}.$$

Note that the expression for $var(Z(F(\hat{\rho })))$ provided in equation (3) of Zou^6^ is not correct, so we use $var(Z(F(\hat{\rho })))$ as specified above. The confidence limits for ρ can be obtained directly by back-transforming as

(16) $$U,L=\frac{exp(2U_{ZF})-1}{exp(2U_{ZF})+k-1},\;\frac{exp(2L_{ZF})-1}{exp(2L_{ZF})+k-1}.$$

We denote this method as $ZF_{\rho }$ . Note that for k=2 , the confidence intervals based on the transformed *F*-statistic and the normalized ICC with the Swiger variance (following equations (.1) and (14)), are the same.

## 3. Simulation comparison of the confidence interval methods

We set up a Monte Carlo simulation to evaluate the statistical properties of the eight confidence interval methods described in Section 2.3. Based on the ANOVA model defined in equation (1), n participant effects ( $s_{i}$ ) are drawn from a standard normal distribution. Then, N (= nk ) errors ( $\epsilon_{ij}$ ) are drawn from a normal distribution, with zero mean and variance determined by the relation in equation (2) for a given value of ρ . This process is replicated 25,000 times. For each replication, the confidence interval using the eight methods described in Section 2.3 is obtained. We study the properties of the methods for values of k varying from 2 to 10 (in steps of 1 ), n from 20 to 100 (in steps of 10 ), and ρ from 0.1 to 0.9 (in steps of 0.1 ).

The methods are compared based on the coverage probability and average confidence interval width in each scenario. The coverage probability is defined as the proportion of times the true value of ρ is covered by the confidence intervals across the 25,000 replications. We define coverage probability as acceptable if it falls within the range $1-\alpha \pm z_{0.975}\sqrt{\frac{(1-\alpha )\alpha }{\eta_{sim}}}$ where 1−α is the nominal coverage and $\eta_{sim}(=25,000)$ is the number of simulations. This is the range of proportions from the simulation, where one expects these proportions to lie in 95% of the cases, if the nominal coverage is the true coverage probability. Specifically, for a nominal coverage of 95%, the coverage probabilities from the simulation are expected to lie between 0.947 and 0.953. The average width of a confidence interval is defined as the average difference between the upper and lower limits of a confidence interval over the 25,000 replications. Since a shorter width of the confidence interval is desirable, methods with a smaller average width of the confidence interval are considered to be better.

Table 2 summarizes the results for ρ≥0.7 , while complete results can be found in Supplemental Material 1. Table 2 shows that for k=2 and ρ≥0.7 , $Wald_{Ze}$ , F , $Z_{S}$ (equivalent to $ZF_{\rho }$ ), and $ZF_{\rho }$ provide acceptable coverage for all values of n , while $Z_{F}$ provides acceptable coverage only for n≥40 . $Wald_{S}$ , $Wald_{F}$ , and $Z_{Ze}$ do not provide acceptable coverage (based on sample sizes explored in Table 2, i.e., n≤100 ).

**Table 2.** Summary of the methods which show acceptable coverage for the 95% confidence interval, that is, between 0.947 and 0.953, for the ICC, ρ≥0.7 and different number of raters, k , and participants, n . In each row, the method providing the average minimum width of the confidence interval is marked in bold. For n≥60 , the differences in average width are <0.01 , therefore, none of the methods have been marked bold in those cases.

k	ρ	n	$Wald_{S}$	$Wald_{F}$	$Wald_{Ze}$	F	$Z_{S}$	$Z_{F}$	$Z_{Ze}$	$ZF_{\rho }$
2	0.7–0.9	≥20			✓	✓	✓			✓
		≥40			✓	✓	✓	✓		✓
>2	0.7–0.8	≥20				✓				
		≥40			✓	✓				✓
		≥80	✓		✓	✓			✓	✓
		≥90	✓		✓	✓	✓	✓	✓	✓
	0.9	≥20			✓	✓				
		≥40			✓	✓				✓
		≥50	✓		✓	✓	✓			✓
		≥60	✓		✓	✓	✓		✓	✓
		≥80	✓	✓	✓	✓	✓	✓	✓	✓

ICC: intraclass correlation coefficient.

For k>2 , F still provides acceptable coverage under all scenarios while $ZF_{\rho }$ and $Wald_{Ze}$ only for n≥40 . The coverage of $Z_{S}$ and $Z_{F}$ deteriorates first when increasing k from 2 to 3 , and then improves on increasing k further. These confidence interval methods provide acceptable coverage when ρ≥0.7 for n≥90 . $Z_{Ze}$ on the other hand provides acceptable coverage when ρ≥0.7 for n≥80 . $Wald_{S}$ provides acceptable coverage when ρ≥0.7 for n≥80 , while $Wald_{F}$ provides acceptable coverage only when ρ≥0.9 for n≥90 . The effect of increasing k is not monotonic for some of the confidence interval methods. However, increasing k above 5 does not seem to improve notably the coverage of the methods (see blueSupplemental Material 1).

The confidence interval methods providing the smallest average width most frequently, under the different scenarios, are marked in bold. The difference in average width between the different confidence interval methods decreases from ∼ 0.1 to <0.01 as n increases from 20 to ≥60 . It must be noted here that though $Wald_{Ze}$ provides better coverage compared to $Wald_{F}$ , it has the largest width among the confidence interval methods.

In summary for ρ≥0.7 , $Wald_{Ze}$ , $ZF_{\rho }$ , and F provide acceptable coverage in almost all scenarios.

## 4. Sample size determination

Sample size determination when the number of raters, k , is fixed, is reviewed for three approaches, namely, the width of confidence interval approach, the assurance probability approach, and the testing approach. These sample size approaches require a planning value, ρ , and yield valid results when the initial guess for ρ is accurate. The eight confidence interval methods reviewed in Section 2.3 can be used with each of the three approaches. However, a closed-form formula for sample size determination is not always available, which necessitates numerical evaluation procedures to determine sample sizes.

### 4.1. Width of confidence interval approach

The approach consists in finding the minimum number of participants for a given value of the expected width, ω , of the confidence interval around a planned value of ρ and for a given number of raters k . Bonett^7^ derived an analytical formula based on the Wald confidence interval and the Fisher variance ( $Wald_{F}$ ). We generalize this approach by considering all large sample variance formulas reviewed in Section 2.2.

The expected width of the Wald confidence interval is given by $\omega =2z_{1-\alpha /2}\sqrt{var(\hat{\rho })}$ , where 1−α is the confidence level and the variance can be estimated using equations (4) to (6). Using the Swiger variance (equation (4)), under the approximation N≈N−1 and taking the positive root, the minimum number of participants is given by (see Appendix B.1 for the derivation)

(17) $$n=1+\frac{8A_{k,\rho }^{2}z_{1-\alpha /2}^{2}}{k(k-1)\omega^{2}},$$

where $A_{k,\rho }=(1-\rho )\times [1+(k-1)\rho ]$ . Using the Fisher variance (equation (5)), the expression for the required minimum number of participants is the same as equation (17), but subtracting one participant. Bonett^7^ used the Fisher variance with n−1 in the denominator of equation (5) instead of n . As a result, the sample size derived by Bonnet is the same as equation (17).

Using the Zerbe variance (equation (6)), the minimum number of participants obtained under the assumption that $\frac{N-3}{N-k}\approx 1$ is:

(18) $$\begin{array}{r} n=[(A_{\omega }^{3}+24A_{\omega }^{2}+103A_{\omega }+16+6\sqrt{3A_{\omega }}\sqrt{4A_{\omega }^{2}+71A_{\omega }+8}{)}^{\frac{1}{3}}\\ +(A_{\omega }^{3}+24A_{\omega }^{2}+103A_{\omega }+16-6\sqrt{3A_{\omega }}\sqrt{4A_{\omega }^{2}+71A_{\omega }+8}{)}^{\frac{1}{3}}\\ +A_{\omega }+8]\times \frac{1}{3(k-1)}, \end{array}$$

where $A_{\omega }=\frac{8z_{1-\alpha /2}^{2}A_{k,\rho }^{2}}{k\omega^{2}}$ and $A_{k,\rho }=(1-\rho )\times [1+(k-1)\rho ]$ (see Appendix B.2 for the derivation).

Giraudeau and Mary^29^ provided an approximate formula for the width of the confidence interval obtained with the Searle method which coincides with the width obtained using the Wald confidence interval with the Fisher variance. Analytical formulas can hardly be obtained for the Searle and the normalization methods. Hence, we propose a general numerical procedure to determine the minimum sample size, n , which can be used with all confidence interval methods. Specifically, this numerical evaluation method consists of finding the expected width of the confidence interval for the specified values of ρ and k . This is done for every n , starting from n=4 and increasing n by one unit at a time. The minimum sample size is the smallest value of n for which the expected width of confidence interval is smaller or equal to ω . Bonett^7^ and Shieh^19^ used a similar numerical approach to obtain sample sizes for F .

Table 3 shows the minimal sample sizes obtained by using the numerical evaluation for ω ∈ {0.1,0.2}, ρ ∈ {0.7,0.8,0.9}, and k ∈ {2,3,6}. The values within parentheses indicate sample sizes obtained using equation (17), equation (17) with a subtraction of one participant and equation (18) for $Wald_{S}$ , $Wald_{F}$ , and $Wald_{Ze}$ , respectively. It can be observed that the sample sizes obtained with the different confidence interval methods are rather close. Sample sizes providing acceptable coverage (the calculation of the acceptable range is given in Section 3) for different combinations of ω , ρ , and k , are marked in bold. Table 3 indicates that the confidence interval methods $Wald_{Ze}$ , F , and $ZF_{\rho }$ provide sample sizes with acceptable coverage in most cases. Note that the numerical approach of Bonnett^7^ and Shieh^19^ leads to sample sizes very close to the values we obtain (data not shown).

**Table 3.** The minimum number of participants, n , required to achieve an expected width, ω , of the 95% confidence interval, given ρ and the number of raters, k , according to the numerical evaluation method. Sample sizes that provide coverage within an acceptable range (based on 25,000 simulations, i.e. between 0.947 and 0.953) are marked in bold. The values in parentheses indicate sample sizes obtained with analytical formulas given in equations (17) for $Wald_{S}$ , (17) with a subtraction of one participant for $Wald_{F}$ , and (18) for $Wald_{Ze}$ , respectively.

ω	ρ	k	$Wald_{S}$	$Wald_{F}$	$Wald_{Ze}$	F	$Z_{S}$	$Z_{F}$	$Z_{Ze}$	$ZF_{\rho }$
0.1	0.7	2	**401** (401)	400 (400)	**408** (408)	**403**	**402**	**401**	**409**	**402**
		3	**267** (267)	266 (266)	**270** (270)	**267**	**267**	**267**	**271**	**267**
		6	**188** (188)	**187** (187)	**189** (188)	**187**	**189**	**188**	**190**	**188**
	0.8	2	**200** (200)	**200** (199)	**207** (207)	**204**	**202**	**202**	**209**	**202**
		3	**140** (139)	139 (138)	**143** (142)	**140**	**141**	**141**	**145**	**141**
		6	**104** (103)	**103** (102)	**105** (104)	**103**	**105**	**104**	**106**	**104**
	0.9	2	56 (56)	56 (55)	**63** (63)	**61**	**60**	**59**	67	**60**
		3	41 (41)	41 (40)	**45** (44)	**43**	**44**	43	47	**44**
		6	32 (32)	31 (31)	**34** (33)	**32**	34	33	**35**	34
0.2	0.7	2	101 (101)	100 (100)	**108** (108)	**103**	**102**	**102**	109	**102**
		3	68 (67)	67 (66)	**71** (70)	**67**	**68**	**68**	72	**68**
		6	48 (48)	47 (47)	**49** (48)	**47**	49	48	**50**	**48**
	0.8	2	51 (51)	50 (50)	**57** (57)	**54**	**53**	53	60	**53**
		3	36 (36)	35 (35)	**39** (38)	**36**	37	37	41	37
		6	27 (27)	26 (26)	**28** (27)	**26**	28	27	29	27
	0.9	2	15 (15)	14 (14)	21 (21)	**19**	18	17	24	18
		3	11 (11)	11 (10)	**14** (14)	13	13	13	16	13
		6	9 (9)	8 (8)	**10** (9)	**9**	11	10	12	10

### 4.2. Assurance probability approach

The assurance probability approach based on the width of the confidence interval for ρ ,^19^ consists of finding the minimum number of participants n such that

(19) P(W≤ω)≥1−γ,

where P(W≤ω) is the probability that the width W , is less than or equal to a constant, ω , and 1−γ is the assurance probability. The assurance probability approach based on the width of the confidence interval was introduced by Zou,^6^ who pointed out that the width of confidence interval approach seen in the previous subsection is a special case, which corresponds to setting the assurance probability to 0.5 . Zou^6^ also introduced an assurance probability approach based on the lower limit of a confidence interval, see Section 4.3.

Zou^6^ derived an analytical formula based on the Wald confidence interval and the Fisher variance ( $Wald_{F}$ ). Shieh^19^ later extended the approach numerically to the Searle method ( F ). In this article, we numerically generalize the assurance probability approach by considering all the confidence interval methods mentioned in Section 2.3.

Using the Wald confidence interval and the Fisher variance (equation (5)), Zou^6^ obtained the minimum number of participants as

(20) $$\begin{array}{rl} n=\frac{1}{k(k-1)\omega^{2}} & [4A_{k,\rho }z_{1-\alpha /2}(A_{k,\rho }z_{1-\alpha /2}+B_{k,\rho }z_{1-\gamma }\omega )\;+\\ & \sqrt{16A_{k,\rho }^{3}z_{1-\alpha /2}^{3}(A_{k,\rho }z_{1-\alpha /2}+2B_{k,\rho }z_{1-\gamma }\omega )}], \end{array}$$

where $A_{k,\rho }=(1-\rho )\times [1+(k-1)\rho ]$ and $B_{k,\rho }=2(k-1)\rho -k+2$ . Zou^6^ used n−1 in equation (5) and derived the formula considering the half-width of the confidence interval. As a result, the formula in Zou has different coefficients than equation (20). Using the Swiger variance (equation (4)), we derived the sample size under the approximation that N≈N−1 (taking the positive root). This leads to equation (20) with the addition of one participant.

The analytical forms of the other confidence interval methods (including $Wald_{Ze}$ ) are too complex. Therefore, we propose a generalization of the numerical approach explained in Section 4.1, which uses assurance probability functions to find the minimum n satisfying a pre-defined value of the assurance probability ( 1−γ ). This numerical procedure works with all the confidence interval methods mentioned in Section 2.3. The derivation of the assurance probability functions are given in Appendix C.

Table 4 shows the sample sizes obtained by the numerical procedure using assurance probability functions. The analytical counterparts are shown between parentheses (when available). It can be observed that the minimum sample sizes obtained analytically are close to the values obtained via the numerical procedure. Further, our method gives sample sizes close to the ones obtained by Shieh, who also used a numerical method for F (Tables 8 and 9 of Shieh^19^). It can be further observed that the sample sizes obtained by the different confidence interval methods are rather close. Sample sizes providing acceptable assurance probability under different combinations of ω , ρ , and k , for 1−γ=0.9 are marked in bold. The lower limit of the acceptable range of assurance probabilities is calculated in the same way as in Section 3 where 1−α is replaced by 1−γ . Confidence interval methods F , $Z_{S}$ , $Z_{F}$ , and $Z_{Ze}$ provide sample sizes with acceptable assurance probability in most cases while $ZF_{\rho }$ for k=2 only.

**Table 4.** Minimum number of participants, n , required to achieve an expected width, ω , of the 95% confidence interval, given ρ , the number of raters, k , and the assurance probability 1−γ=0.9 , according to the numerical procedure using assurance probability functions. Sample sizes that provide acceptable empirical assurance probability (i.e. above 0.896 for 25,000 simulations) are marked in bold. The values in parentheses indicate sample sizes obtained from the analytical formulas given in equation (20) for $Wald_{F}$ and equation (20) with an addition of 1 participant for $Wald_{S}$ , respectively.

ω	ρ	k	$Wald_{S}$	$Wald_{F}$	$Wald_{Ze}$	F	$Z_{S}$	$Z_{F}$	$Z_{Ze}$	$ZF_{\rho }$
0.1	0.7	2	**470** (470)	469 (469)	**477**	**473**	**472**	**471**	**479**	**473**
		3	**309** (308)	**308** (307)	**312**	**309**	**314**	**313**	**317**	308
		6	**214** (214)	213 (213)	**216**	**214**	**221**	**220**	**222**	213
	0.8	2	255 (255)	254 (254)	**262**	**260**	**259**	**258**	**266**	**260**
		3	176 (176)	175 (175)	179	**178**	**180**	**180**	**184**	177
		6	129 (129)	128 (128)	130	**130**	**134**	**133**	**135**	129
	0.9	2	87 (87)	87 (86)	94	**94**	**93**	**93**	**100**	**94**
		3	63 (63)	63 (62)	67	**67**	**68**	**67**	**71**	66
		6	49 (49)	48 (48)	50	**52**	**53**	**52**	**54**	50
0.2	0.7	2	134 (134)	**134** (133)	**141**	**137**	**136**	**135**	**143**	**137**
		3	**88** (88)	87 (87)	**91**	**88**	**91**	**90**	**94**	87
		6	**61** (61)	**60** (60)	**62**	**60**	**64**	**64**	**66**	59
	0.8	2	77 (77)	76 (76)	84	**81**	**80**	**80**	**87**	**81**
		3	53 (53)	53 (52)	56	**55**	**56**	**56**	**60**	54
		6	39 (39)	38 (38)	40	**40**	**42**	**41**	**43**	39
	0.9	2	29 (29)	29 (28)	36	**35**	**34**	**34**	**41**	**35**
		3	22 (22)	21 (21)	25	**25**	**25**	24	**28**	24
		6	17 (17)	16 (16)	18	**19**	**20**	**19**	**21**	18

### 4.3. Testing approach

The testing approach consists of finding the minimum number of participants when one is interested in achieving a pre-specified power (1−β) when testing the null hypothesis that ρ is less than or equal to a constant, $\rho_{0}$ , that is, $\rho \leq \rho_{0}$ , against the alternative that ρ is greater than $\rho_{0}$ , that is, $\rho >\rho_{0}$ . Denoting $\rho =\rho_{A}$ under the alternative hypothesis, the power of this test can be defined as the probability that the null hypothesis is rejected when the alternative hypothesis is true ( $\rho =\rho_{A}$ ). In our case, this is the probability that the lower limit L of the confidence interval for ρ is greater than $\rho_{0}$ when the alternative hypothesis is true. The mathematical form of the criterion under this approach can be written as,^6^

(21) $$P(L\geq \rho_{0}|\rho =\rho_{A})\geq 1-\beta ,$$

where $P(L\geq \rho_{0}|\rho =\rho_{A})$ is the probability that the lower limit of the confidence interval for ρ , L , is greater than the pre-specified value, $\rho_{0}$ , under the alternative hypothesis that $\rho =\rho_{A}$ $\left(1>\rho_{A}>\rho_{0}>0\right)$ . Donner and Eliasziw,^10^ Walter et al.,^9^ and Zou^6^ derived an analytical formula for the minimum number of participants, n , based on the transformation of the *F*-statistic ( $ZF_{\rho }$ ) when minimizing the criterion specified in equation (.1). Specifically,

(22) $$n=1+\frac{2(z_{1-\beta }+z_{1-\alpha }{)}^{2}k}{\left[ln(F(\rho_{A})/F(\rho_{0})){]}^{2}(k-1\right)}.$$

Shieh^21^ used a numerical evaluation procedure to obtain sample sizes for the Searle method. Zou^6^ obtained equation (22) by introducing an assurance probability based on a pre-specified lower limit of an asymmetrical interval procedure, which is equivalent to the testing approach.

We derived power functions following equation (.1) for all the confidence interval methods (see Appendix D). These power functions were then used to obtain sample sizes for the testing approach using the numerical procedure mentioned in Section 4.2. The numerical procedure uses the power functions to find the minimum n satisfying a pre-defined power ( 1−β ).

Table 5 shows the sample sizes obtained by the numerical procedure using the power functions and numerical evaluation. The values within parentheses indicate sample sizes obtained by the analytical formulas for the method $ZF_{\rho }$ which correspond exactly to the ones obtained by our numerical procedure. The values obtained for the method F using our numerical procedure are exactly one unit greater than the values obtained by the numerical method of Shieh.^21^ Furthermore, unlike the previous approaches, the sample sizes obtained via the Wald confidence interval methods tend to require smaller sample sizes than other confidence interval methods. The actual power of the hypothesis test was also calculated at the obtained sample sizes. Sample sizes providing acceptable power for different combinations of 1−β , $\rho_{0}$ , $\rho_{A}$ , and k are marked in bold. The lower limit of the acceptable range of power is calculated in the same way as in Section 3, where 1−α is replaced by 1−β . The confidence interval methods F and $Z_{Ze}$ provide the sample sizes with acceptable power in most cases while $Z_{S}$ , $Z_{F}$ , and $ZF_{\rho }$ provide sample sizes with acceptable power for k=2 only. The Wald methods always have power below acceptable value (i.e. < 0.795 when 1−β=0.8 , and 0.896 when 1−β=0.9 ). For example, the actual power for the Wald methods can go as low as 0.729 which is the case for 1−β=0.8 , $\rho_{0}=0.7$ , $\rho_{A}=0.8$ , and k=2 .

**Table 5.** Minimum number of participants, n , for a given value of ρ considering the null ( $\rho_{0}$ ) and alternative hypothesis ( $\rho_{A}$ ) for a specified number of raters, k , and power of the test 1−β according to the numerical procedure using power functions. Sample sizes that provide acceptable empirical power (i.e. above 0.896 when 1−β=0.9 and above 0.795 when 1−β=0.8 for 25,000 simulations) are marked in bold. The values in parentheses indicate the sample sizes obtained from the analytical formula given in equation (22) for $ZF_{\rho }$ .

1−β	$\rho_{0}$	$\rho_{A}$	k	$Wald_{S}$	$Wald_{F}$	$Wald_{Ze}$	F	$Z_{S}$	$Z_{F}$	$Z_{Ze}$	$ZF_{\rho }$
0.9	0.7	0.8	2	112	111	119	**162**	**161**	**161**	**168**	**162** (162)
			3	78	78	82	**110**	112	112	**116**	110 (110)
			6	58	58	60	**80**	**84**	**83**	**85**	80 (80)
	0.8	0.9	2	32	31	38	**63**	**62**	**62**	**69**	**63** (63)
			3	24	23	27	**45**	**46**	45	**49**	45 (45)
			6	19	18	20	**34**	36	35	**37**	35 (35)
0.8	0.7	0.8	2	81	81	88	**117**	**117**	**116**	**123**	**117** (117)
			3	57	56	60	**79**	**82**	**81**	**85**	80 (80)
			6	43	42	44	**57**	61	60	**62**	58 (58)
	0.8	0.9	2	23	23	30	**46**	**45**	**45**	**52**	**46** (46)
			3	17	17	20	**32**	33	33	**36**	33 (33)
			6	14	13	15	**25**	26	25	27	25 (25)

### 4.4. Software for sample size calculation

Currently, only the method $Wald_{F}$ is available in common software (see Appendix E) for the width of the confidence interval and assurance probability approaches, while only $ZF_{\rho }$ is available for the testing approach. Therefore, a Shiny app containing all the approaches to determine minimum required sample sizes has been developed^30^ and made available on https://github.com/DiproMondal/sample-size-ICCGithub and the https://dipro.shinyapps.io/sample-size-icc/Shiny server.

## 5. Empirical illustration

### 5.1. Reliability of systolic blood pressure measurements

In this section, we illustrate how the confidence interval methods described in Section 2.3 and the approaches for sample size determination described in Section 4 are used in the context of a reliability study. In the study of Bland and Altman,^31^ three repeated systolic blood pressure measurements ( k=3 ) were made on 85 participants ( n=85 ) by two experienced observers raters J and R and a semi-automatic blood pressure monitor. For the purpose of our illustration, we use the measurements made by rater J only, which can be modeled by a one-way ANOVA.

The ANOVA model assumes that the outcome measurements are normally distributed and the variance across repetitions is homogeneous across participants. Exploratory data analysis revealed that the excess kurtosis for the repetitions was mild while the degree of asymmetry of the repetitions indicated moderate skewness. Furthermore, the data also present mild heteroscedasticity on the repeated measurements. Following equation (3), we obtain $\hat{\rho }=0.962$ . The confidence intervals obtained using the eight confidence interval methods are shown in Table 6, which have rather similar bounds.

**Table 6.** Lower and upper limits of the 95% confidence intervals for ρ for the systolic blood pressure measurements.

	$Wald_{S}$	$Wald_{F}$	$Wald_{Ze}$	F	$Z_{S}$	$Z_{F}$	$Z_{Ze}$	$ZF_{\rho }$
Lower limit	0.948	0.948	0.947	0.945	0.945	0.945	0.945	0.945
Upper limit	0.975	0.975	0.976	0.974	0.973	0.973	0.973	0.973

### 5.2. Planning a reliability study

A researcher may be interested in planning a study to measure blood pressure aiming at a reliability of ρ=0.9 . The sample size approaches described in previous sections can be used to find the number of participants required for such a study.

**Figure 1.**
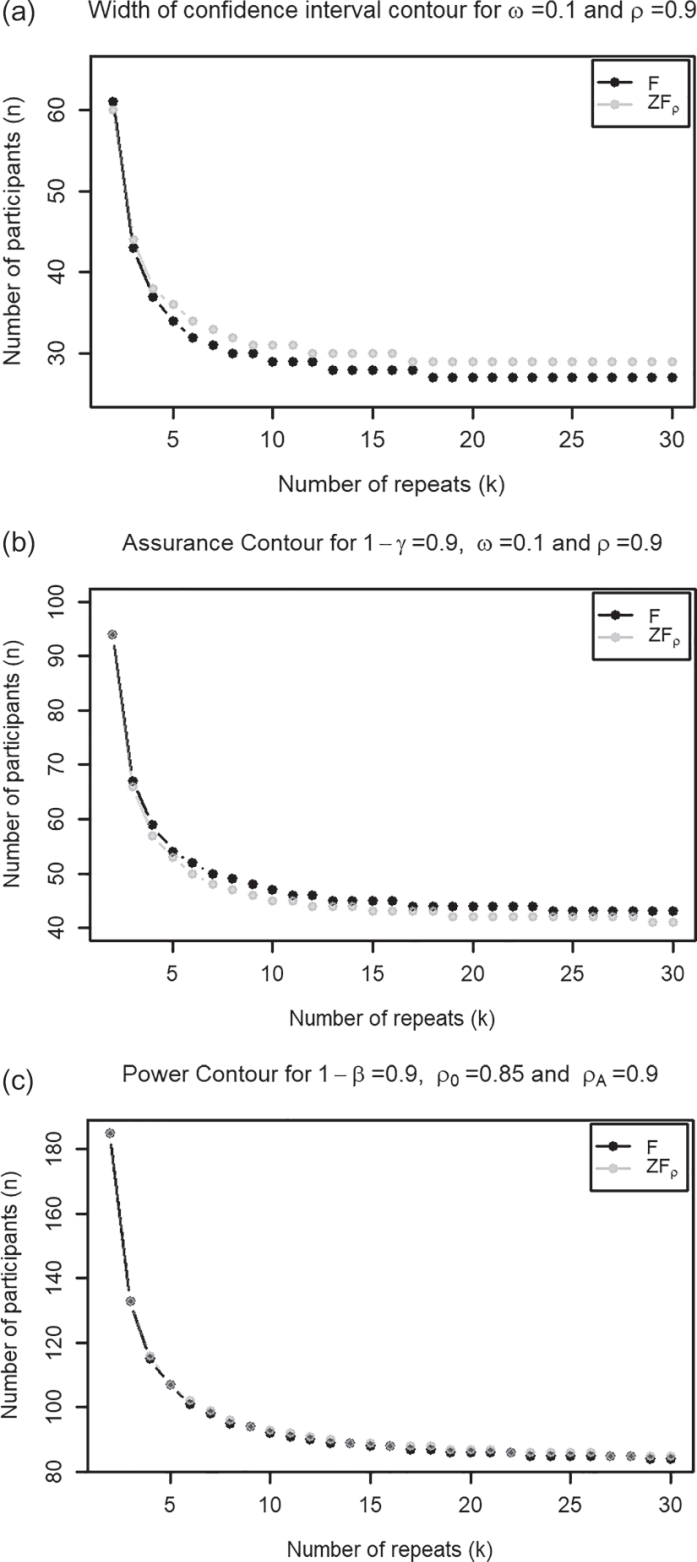
Combinations for n and k for the confidence interval methods F and $ZF_{\rho }$ satisfying the criteria specified for the three different sample size approaches. The criteria for the sample size approaches are mentioned in the sub-figures where, for the width of confidence interval approach, ω is the expected width of the confidence interval around a given value ρ ; for the assurance probability approach, the notations are the same as the width of confidence interval approach with the addition of 1−γ denoting the assurance probability; for the testing approach 1−β is the power of the hypothesis test, $\rho_{0}$ and $\rho_{A}$ are the values under the null and alternative hypotheses. (a) Width of confidence interval approach; (b) assurance probability approach; and (c) testing approach.

Figure 1 shows, for each k in the interval [2,30] (*x*-axis), the minimum required n (*y*-axis) using (top-down) the width of confidence interval approach described in Section 4.1, the assurance probability approach described in Section 4.2, and the testing approach described in Section 4.3 for the confidence interval methods F and $ZF_{\rho }$ . For example, suppose the study only allows for three repeated measurements per participant. Then using the width of confidence interval approach, the assurance probability approach, and the testing approach the researcher would require, respectively, 43, 67, and 133 (considering the Searle confidence interval method) participants for the criteria given in Figure 1. The effect of increasing the number of measurements per participant to four is a decrease in the number of participants to 37, 59, and 115 participants (considering the Searle confidence interval method), respectively, for the width of confidence interval approach, the assurance probability approach, and the testing approach. The gain in having a smaller number of participants for the study decreases as the number of measurements per participant increases.

If, instead, there is flexibility in choosing the number of repetitions per participant, the researcher can consider a cost-constraint approach to find the optimal combination of the number of participants ( n ) and number of repeated measures per participant ( k ). Then, the optimal combination of (k,n) is obtained by finding the value of k and n for which the total cost, *T*, is minimum. A plausible cost function is

(23) $$T=nc_{1}+nkc_{2},$$

where T is the total cost, $c_{1}$ is the cost of recruiting a participant, and $c_{2}$ is the cost of making one observation.

Table 7 shows the optimal combinations of (k,n) obtained by minimizing the total cost, T (equation (23)), for different combinations of $c_{1}$ and $c_{2}$ . It can be observed from the table that as $c_{1}$ increases relative to $c_{2}$ , more repetitions per participant are required with a smaller number of participants to achieve the same criterion value.

**Table 7.** Optimal combination of the number of repetitions and participants, ( k,n ) for the sample size approaches with the confidence interval methods, F and $ZF_{\rho }$ , for different costs of recruiting a participant, $c_{1}$ and making an observation, $c_{2}$ .

Sample size approach	$c_{1}$	$c_{2}$	F	$ZF_{\rho }$
Width of confidence interval approach for ω=0.1 and ρ=0.9	1	5	(261)	(260)
	1	1	(343)	(344)
	5	1	(437)	(438)
Assurance probability approach for 1−γ=0.9 , ω=0.1 , and ρ=0.9	1	5	(294)	(295)
	1	1	(367)	(367)
	5	1	(459)	(458)
Testing approach for 1−β=0.9 , $\rho_{0}=0.85$ , and $\rho_{A}=0.9$	1	5	(2185)	(2185)
	1	1	(3133)	(3133)
	5	1	(4115)	(4116)

## 6. Discussion

Sample size determination is a crucial aspect of the planning stage of a reliability study. Usually, the number of raters, k , is fixed due to budget or time constraints in the study, and the sample size of participants, n , needs to be determined. This article gives a complete overview of the different approaches available in that case. Analytical closed-form solutions for sample size determination only exist in a few cases. Therefore, we proposed a general procedure that entails deriving an assurance probability or power function (depending on the approach) and finding optimal n via a simple search procedure.

Before inspecting the different approaches for sample size determination, we looked at the statistical properties of the different confidence interval methods. We have shown that the confidence interval based on the Searle method ( F ) provides acceptable coverage in almost all scenarios for n≥20 , and, $Wald_{Ze}$ and $ZF_{\rho }$ for n≥40 . This can be explained by the fact that F is an exact method and $ZF_{\rho }$ is based on a normalizing transformation of F . $Wald_{Ze}$ is the Wald method based on the Zerbe variance which was also derived as a ratio of *F*-statistics. It must be noted however, that $Wald_{Ze}$ does not provide acceptable coverage for small ρ (when ρ<0.5 , see blueSupplemental Material 1). The other methods, based on some approximations, only provide acceptable coverage in few scenarios. It is worthwhile to note that the Wald confidence interval using the Fisher variance, $Wald_{F}$ , widely used in the literature shows acceptable coverage only for large sample sizes, n≥80 , when ρ≥0.9 and k>2 . Note that the Zerbe variance provides better statistical properties than the Fisher variance when ρ≥0.7 , but the width of the confidence interval is larger.

Sample sizes were determined using three different approaches which rely on the limits of a confidence interval for ρ . Sample sizes in the case of the width of confidence interval were obtained via a numerical evaluation. We derived the assurance probability and power functions for assurance probability and testing approaches, respectively, to determine sample sizes. These functions, when combined with the numerical evaluation, enabled us to determine sample sizes for all the methods discussed. Sample sizes obtained through this procedure and the corresponding available analytical formulas led to similar sample sizes. Furthermore, sample sizes obtained with different confidence interval methods in the width of confidence interval approach and the assurance probability approach were similar. However, this was not the case in the testing approach where smaller sample sizes were obtained using the Wald confidence interval to achieve a required power level compared to the other confidence interval methods. This is probably because the Wald confidence interval method assumes a symmetric distribution for the estimate of ρ , which is not a realistic assumption when ρ is large (e.g. 0.8, 0.9).^28^ In all the approaches, the Searle method ( F ) provided sample sizes with good statistical properties as well as $ZF_{\rho }$ when k=2 . We, therefore, advise the use of these methods to make statistical inference on the ICC in the one-way ANOVA setting.

We have shown that the choice of the approach to determine sample size or even the choice of the confidence interval method, has an impact on the resulting sample size. We, therefore advise researchers to carefully consider requirements for their studies as a guide to choose the appropriate sample size approach. For the three different approaches discussed in this article, the Searle confidence interval method demonstrated good statistical properties, making it our recommended choice. Furthermore, in order to determine sample sizes, we have developed an R Shiny app which we believe will prove valuable to researchers in need of a simple and efficient interface for obtaining sample sizes.

Our study is not without limitations. First, the confidence interval methods investigated in this paper, except the Searle confidence interval method (which is exact), rely on large sample approximations. Therefore, practitioners should exercise caution when calculations lead to a small minimal sample size because a good statistical behavior is not guaranteed. Note that the minimal sample sizes obtained with the different approaches rarely go below 20 in realistic scenarios (see Tables 3 to 5). Second, the estimator of ρ and its confidence interval rely on the assumptions of normality and homoscedasticity in line with the one-way ANOVA model (equation (1)). Violations of these conditions impact the statistical properties of the confidence intervals. The effect of non-normality on the Type-I error rate of the *F*-statistic was studied by various authors.^32–37^ However, simulation studies^38^ showed that the effect of heteroscedasticity outweighs the effect of non-normality on the Type-I error rate of the *F*-statistic, even for a balanced design^39^ as considered here. We, therefore, advise researchers to check for violations of the assumptions of the ANOVA model (equation (1)) before using the methods described in this article. Readers interested in non-parametric estimators of ICC, not requiring the normality assumption, are directed to the works of Rothery,^40^ Shirahata,^41^ Commenges and Jacqmin,^42^ and Ukoumunne et al.^43^ Note that, however, these papers do not develop a sample size procedure. Third, as previously mentioned, we consider an equal number of ratings per participant constituting a balanced design. Considering unbalanced designs will require specifying the degree of imbalance in advance, which is not an easy task. Furthermore, Donner^14^ showed that with an unbalanced design, the *F*-statistic is not exact and this, in turn, affects the statistical properties of the ICC and its confidence interval. Fourth, we focused on reliability in the context of a one-way ANOVA model. Whether the numerical procedure we developed can be extended to multi-way ANOVA models, will require further investigation, as methods to construct confidence intervals are different in that case.^44,45^

## Supplemental Material

sj-pdf-1-smm-10.1177_09622802231224657 - Supplemental material for Review of sample size determination methods for the intraclass correlation coefficient in the one-way analysis of variance model Supplemental material, sj-pdf-1-smm-10.1177_09622802231224657 for Review of sample size determination methods for the intraclass correlation coefficient in the one-way analysis of variance model by Dipro Mondal, Sophie Vanbelle, Alberto Cassese and Math JJM Candel in Statistical Methods in Medical Research

## Appendix A. Ratio of variance estimators

We will show that,

$$\begin{array}{r} var(\hat{\rho }{)}_{Ze}>var(\hat{\rho }{)}_{S}>var(\hat{\rho }{)}_{F}, \end{array}$$

and thus

$$\begin{array}{rl} & \Omega (\hat{\rho }{)}_{Ze}>\Omega (\hat{\rho }{)}_{S}>\Omega (\hat{\rho }{)}_{F} \end{array}$$

where $\Omega (\hat{\rho }{)}_{m}$ is the average width of the confidence interval based on variance approximation m (m=S,F,Ze) .

Note that the variance estimators (equations (4) to (6)) can be written in the form,

$$var(\hat{\rho }{)}_{m}=(\frac{f(\rho )}{\sqrt{f(n,k{)}_{m}}}{)}^{2}=\frac{(f(\rho ){)}^{2}}{f(n,k{)}_{m}}$$

where $(f(\rho ){)}^{2}=2(1-\rho {)}^{2}[1+(k-1)\rho {]}^{2}$ and $f(n,k{)}_{m}$ depends on the form of the variance approximation m . For the Swiger variance, $f(n,k{)}_{S}=\frac{k^{2}(N-n)(n-1)}{\left(N-1\right)}$ , for the Fisher variance, $f(n,k{)}_{F}=nk(k-1)$ , and for the Zerbe variance, $f(n,k{)}_{Ze}=\frac{k^{2}(n-1)(N-n-2{)}^{2}(N-n-4)}{\left(N-n{)}^{2}(N-3\right)}$ .

1. $\frac{var(\hat{\rho }{)}_{S}}{var(\hat{\rho }{)}_{F}}$ = $\frac{N-1}{N-k}>1$ .
2. $\frac{var(\hat{\rho }{)}_{Ze}}{var(\hat{\rho }{)}_{S}}=\frac{\left(N-n{)}^{3}(N-3\right)}{\left(N-n-2{)}^{2}(N-n-4)(N-1\right)}>1$ .

The proof of 2 is as follows:

We can re-write the ratio of the Zerbe and the Swiger variances into two parts as,

$$\frac{var(\hat{\rho }{)}_{Ze}}{var(\hat{\rho }{)}_{S}}=\frac{(N-n{)}^{2}}{(N-n-2{)}^{2}}\times \frac{\left(N-n)(N-3\right)}{\left(N-n-4)(N-1\right)}$$

We have $\frac{(N-n{)}^{2}}{(N-n-2{)}^{2}}>1$ . For the second part, we have,

$$\begin{array}{r} (N-n)(N-3)-(N-n-4)(N-1)=2(N+n)-4>0\;(\text{for }n\geq 2\;\mathrm{and}\;N\geq 4), \end{array}$$

implying that

$$\begin{array}{r} \frac{\left(N-n)(N-3\right)}{\left(N-n-4)(N-1\right)}>1. \end{array}$$

## Appendix B. Sample size determination in the width of confidence interval approach

The sample size formulas for the width of confidence interval approach are derived here. Using the Wald confidence interval from Section 2.3.1, the minimum value of n is obtained when:

$$2z_{1-\alpha /2}\sqrt{var(\hat{\rho })}\leq \omega ,\text{ i.e.}\;var(\hat{\rho })\leq \frac{\omega^{2}}{4z_{1-\alpha /2}^{2}},$$

where ω is the expected width of the confidence interval, $z_{1-\alpha /2}$ is the (1−α/2)×100 percentile of the standard normal distribution and $var(\hat{\rho })$ can be determined by equations (4) to (6).

### B.1 Width of confidence interval approach with the Wald confidence interval and the Swiger variance

Using the Swiger variance formula (equation (4)), we have:

$$\begin{array}{rl} & \frac{2(nk-1)(1-\rho {)}^{2}[1+(k-1)\rho {]}^{2}}{k^{2}n(k-1)(n-1)}=\frac{\omega^{2}}{4z_{1-\alpha /2}^{2}},\\ & \;\;⟺\;\frac{n(n-1)}{nk-1}=\frac{8A_{k,\rho }^{2}z_{1-\alpha /2}^{2}}{k^{2}(k-1)\omega^{2}}\;\text{where }A_{k,\rho }=(1-\rho )(1+(k-1)\rho ),\\ & \;\;⟺\;n^{2}-n[1+\frac{8A_{k,\rho }^{2}z_{1-\alpha /2}^{2}}{k(k-1)\omega^{2}}]+\frac{8A_{k,\rho }^{2}z_{1-\alpha /2}^{2}}{k^{2}(k-1)\omega^{2}}=0. \end{array}$$

Solving the last equation yields the following solution:

$$\begin{array}{rl} & n=\frac{k(k-1)\omega^{2}+8A_{k,\rho }^{2}z_{1-\alpha /2}^{2}+\sqrt{[k(k-1)\omega^{2}+8A_{k,\rho }^{2}z_{1-\alpha /2}^{2}{]}^{2}-32A_{k,\rho }^{2}(k-1)\omega^{2}z_{1-\alpha /2}^{2}}}{2k(k-1)\omega^{2}}, \end{array}$$

which leads to the approximation,

$$\begin{array}{r} n\approx \frac{k(k-1)\omega^{2}+8A_{k,\rho }^{2}z_{1-\alpha /2}^{2}+\sqrt{[k(k-1)\omega^{2}+8A_{k,\rho }^{2}z_{1-\alpha /2}^{2}{]}^{2}}}{2k(k-1)\omega^{2}}=n^{*}. \end{array}$$

The other solution (with a negative sign in front of the square root) leads to *n* approximately equal to 0. The excess part $-32A_{k,\rho }^{2}(k-1)\omega^{2}z_{1-\alpha /2}^{2}$ creates a difference <1 between *n* and $n^{*}$ . (By numerical evaluation for k∈{2−20} , ρ∈{0.1−0.9} , ω∈{0.1−0.3} and α∈{0.05,0.01} we observe a maximum difference of 0.5 between *n* and $n^{*}$ .)

$$\begin{array}{r} \;⟺\;n\approx 1+\frac{8A_{k,\rho }^{2}z_{1-\alpha /2}^{2}}{k(k-1)\omega^{2}}. \end{array}$$

### B.2 Width of confidence interval approach with the Wald confidence interval and the Zerbe variance

In a similar way, we obtain with the Zerbe variance (equation (6))

$$\begin{array}{rl} & \frac{2(1-\rho {)}^{2}[1+(k-1)\rho {]}^{2}(nk-n{)}^{2}(nk-3)}{k^{2}(n-1)(nk-n-2{)}^{2}(nk-n-4)}=\frac{\omega^{2}}{4z_{1-\alpha /2}},\\ & \;\;⟺\;\frac{8A_{k,\rho }^{2}z_{1-\alpha /2}^{2}(k-1{)}^{2}(nk-3)}{\omega^{2}k^{2}(n-1)}=\frac{\left(nk-n-2{)}^{2}(nk-n-4\right)}{n^{2}}. \end{array}$$

Assuming $\frac{nk-3}{nk-k}\approx 1$ , leads to the following result

$$\begin{array}{rl} & \frac{8A_{k,\rho }^{2}z_{1-\alpha /2}^{2}}{\omega^{2}k}=\frac{\left(nk-n-2{)}^{2}(nk-n-4\right)}{(nk-n{)}^{2}},\\ & \;\;⟺\;(nk-n{)}^{3}-(8+\frac{8A_{k,\rho }^{2}z_{1-\alpha /2}^{2}}{\omega^{2}k})(nk-n{)}^{2}+20(nk-n)-16=0. \end{array}$$

Solving the last equation gives only one root in the real domain:

$$\begin{array}{rl} n= & [(A_{\omega }^{3}+24A_{\omega }^{2}+103A_{\omega }+16+6\sqrt{3A_{\omega }}\sqrt{4A_{\omega }^{2}+71A_{\omega }+8}{)}^{\frac{1}{3}}\\ & \;+(A_{\omega }^{3}+24A_{\omega }^{2}+103A_{\omega }+16-6\sqrt{3A_{\omega }}\sqrt{4A_{\omega }^{2}+71A_{\omega }+8}{)}^{\frac{1}{3}}\\ & \;+A_{\omega }+8]\times \frac{1}{3(k-1)}. \end{array}$$

where $A_{\omega }=\frac{8z_{1-\alpha /2}^{2}A_{k,\rho }^{2}}{k\omega^{2}}$ .

## Appendix C. Assurance probability functions

The assurance probability functions for the assurance probability approach^6^ are derived here. The criterion for this approach is given as,

P(W≤ω)≥1−γ.

### C.1 Wald confidence interval method

The expected width of the Wald confidence interval is given as $2z_{1-\alpha /2}\sqrt{var(\hat{\rho })}$ . Then the criterion can be further specified as,

$$P(2z_{1-\alpha /2}\sqrt{\hat{var}(\hat{\rho })}\leq \omega )\geq 1-\gamma .$$

Rewriting $\hat{var}(\hat{\rho })=(\frac{f(\hat{\rho })}{\sqrt{f(n,k)}}{)}^{2}$ , with $f(\hat{\rho })$ and f(n,k) defined in Appendix A, we have,

$$\begin{array}{rl} & P(2z_{1-\alpha /2}\frac{f(\hat{\rho })}{\sqrt{f(n,k)}}\leq \omega )\geq 1-\gamma ,\\ & \;\;⟺\;P(f(\hat{\rho })\leq \frac{\omega }{2z_{1-\alpha /2}}\sqrt{f(n,k)})\geq 1-\gamma ,\\ & \;\;⟺\;P(\frac{f(\rho )-f(\hat{\rho })}{\sqrt{var(f(\hat{\rho }))}}\geq \frac{f(\rho )-\frac{\omega }{2z_{1-\alpha /2}}\sqrt{f(n,k)}}{\sqrt{var(f(\hat{\rho }))}})\geq 1-\gamma . \end{array}$$

Using the Delta method, we obtain, $var(f(\hat{\rho }))=var(\rho )\times |f^{\prime }(\rho ){|}^{2}$ . Then, the assurance probability function can be written as,

$$1-\Phi_{z}(\frac{f(\rho )-\frac{\omega }{2z_{1-\alpha /2}}\sqrt{f(n,k)}}{f(\rho )|f^{\prime }(\rho )|}\sqrt{f(n,k)}).$$

where $\Phi_{z}(.)$ is the cumulative standard normal distribution.

### C.2 Searle method

The width of the Searle confidence interval is given as,

$$\frac{F(\hat{\rho })/F_{l}-1}{F(\hat{\rho })/F_{l}+k-1}-\frac{F(\hat{\rho })/F_{u}-1}{F(\hat{\rho })/F_{u}+k-1}=\frac{kF(\hat{\rho })(F_{u}-F_{l})}{\left(F(\hat{\rho })+(k-1)F_{u})(F(\hat{\rho })+(k-1)F_{l}\right)}.$$

Rewriting $\frac{kF(\hat{\rho })(F_{u}-F_{l})}{\left(F(\hat{\rho })+(k-1)F_{u})(F(\hat{\rho })+(k-1)F_{l}\right)}=f_{F}(F(\hat{\rho }))$ , we notice that $f_{F}(.)$ is a decreasing function of $F(\hat{\rho })$ when k=2 , otherwise concave. Assuming $F(\hat{\rho })=x$ , the point of extrema (i.e. $f_{F}^{\prime }(x)=0$ ) is $(k-1)\sqrt{F_{u}F_{l}}$ . Therefore, we can define the inverse function, $f_{F}^{-1}(x)$ when $x>(k-1)\sqrt{F_{u}F_{l}}$ as,

$$f_{F+}^{-1}(x)=\frac{1}{2x}[F_{u}^{*}-F_{l}^{*}+\sqrt{(F_{u}-F_{l})(\frac{F_{u}^{*2}}{F_{u}}-\frac{F_{l}^{*2}}{F_{l}})}\;],$$

and the inverse function when $x\leq (k-1)\sqrt{F_{u}F_{l}}$ as,

$$f_{F-}^{-1}(x)=\frac{1}{2x}[F_{u}^{*}-F_{l}^{*}-\sqrt{(F_{u}-F_{l})(\frac{F_{u}^{*2}}{F_{u}}-\frac{F_{l}^{*2}}{F_{l}})}\;],$$

where $F_{u}^{*}=F_{u}(k-(k-1)x)$ and $F_{l}^{*}=F_{l}(k+(k-1)x)$ . Following the criterion for the assurance probability approach,

$$\begin{array}{rl} & P(\frac{kF(\hat{\rho })(F_{u}-F_{l})}{\left(F(\hat{\rho })+(k-1)F_{u})(F(\hat{\rho })+(k-1)F_{l}\right)}\leq \omega )\geq 1-\gamma ,\\ & \;\;⟺\;P(f_{F}(F(\hat{\rho }))\leq \omega )\geq 1-\gamma ,\\ & \;\;⟺\;P(F(\hat{\rho })\leq f_{F-}^{-1}(\omega ))+P(F(\hat{\rho })>f_{F+}^{-1}(\omega ))\geq 1-\gamma . \end{array}$$

Then, the assurance probability function can be written as,

$$1+\Phi_{F}(\frac{f_{F-}^{-1}(\omega )}{\tau })-\Phi_{F}(\frac{f_{F+}^{-1}(\omega )}{\tau }),$$

where $\Phi_{F}(.)$ is the cumulative *F* distribution with (n−1) and n(k−1) degrees of freedom, $\tau =\frac{1+(k-1)\rho }{1-\rho }$ , and $f_{F-}^{-1}(.)$ and $f_{F+}^{-1}(.)$ are defined above.

### C.3 Normalized ICC method

The width of the normalized ICC confidence interval method is given as,

$$\frac{exp(2U_{Z})-1}{exp(2U_{Z})+1}-\frac{exp(2L_{Z})-1}{exp(2L_{Z})+1}.$$

Denoting $x=2Z(\hat{\rho })$ and $\delta =2z_{1-\alpha /2}\sqrt{var(Z(\hat{\rho }))}$ , assuming the variance of $Z(\hat{\rho })$ to be known, we can rewrite the width of the normalized ICC confidence interval method as,

$$\begin{array}{rl} & \frac{exp(x)exp(\delta )-1}{exp(x)exp(\delta )+1}-\frac{exp(x)exp(-\delta )-1}{exp(x)exp(-\delta )+1},\\ & \;=\frac{2exp(x-\delta )(exp(2\delta )-1)}{\left(exp(x)+exp(\delta ))(exp(x)+exp(-\delta )\right)},\\ & \;=\frac{4exp(x)}{\left(exp(x)+exp(\delta ))(exp(x)+exp(-\delta )\right)}\times \frac{exp(2\delta )-1}{2exp(\delta )}. \end{array}$$

which after some algebraic manipulation and using Euler’s transformation of the hyperbolic function is equal to $\frac{2sinh(\delta )}{cosh(\delta )+cosh(x)}$ . Then, following the criterion for the assurance probability approach,

$$\begin{array}{rl} & P(\frac{2sinh(\delta )}{cosh(\delta )+cosh(x)}\leq \omega )\geq 1-\gamma ,\\ & \;\;⟺\;P(\frac{cosh(x)}{2sinh(\delta )}\geq \frac{1}{\omega }-\frac{cosh(\delta )}{2sinh(\delta )})\geq 1-\gamma ,\\ & \;\;⟺\;P(cosh(x)\geq \frac{2sinh(\delta )}{\omega }-cosh(\delta ))\geq 1-\gamma . \end{array}$$

Denoting $A=\frac{2sinh(\delta )}{\omega }-cosh(\delta )$ , then,

$$\begin{array}{rl} & cosh(x)=A,\\ & \;⟺\;e^{x}-2A+e^{-x}=0,\\ & \;⟺\;e^{2x}-2Ae^{x}+1=0. \end{array}$$

which gives $x=ln(A\pm \sqrt{A^{2}-1})$ . Note that cosh(.) is a convex function ( $\frac{\partial^{2}}{\partial x^{2}}cosh(x)=cosh(x)=\frac{e^{x}+e^{-x}}{2}>0$ , since, $e^{x}>0$ and $e^{-x}>0$ , ∀x∈R ). Therefore,

$$\begin{array}{rl} & P(cosh(x)\geq A)=\left\{ \begin{array}{ll} P(x\leq ln(A-\sqrt{A^{2}-1}))+P(x\geq ln(A+\sqrt{A^{2}-1})) & \text{if}\;A\geq \;1\\ 1 & \text{if}\;A<1 \end{array}\right.,\\ & \;=\left\{ \begin{array}{ll} P(Z(\hat{\rho })\leq \frac{1}{2}ln(A-\sqrt{A^{2}-1}))+P(Z(\hat{\rho })\geq \frac{1}{2}ln(A+\sqrt{A^{2}-1})) & \text{if}\;A\geq \;1\\ 1 & \text{if}\;A<1 \end{array}\right.. \end{array}$$

Therefore, the assurance probability can be approximated by,

$$\begin{array}{r} \left\{ \begin{array}{ll} \Phi (\frac{\frac{1}{2}ln(A-\sqrt{A^{2}-1})-E(Z(\hat{\rho }))}{\sqrt{var(Z(\hat{\rho }))}})+1-\Phi (\frac{\frac{1}{2}ln(A+\sqrt{A^{2}-1})-E(Z(\hat{\rho }))}{\sqrt{var(Z(\hat{\rho }))}}) & \text{if}\;A\geq \;1\\ 1 & \text{if}\;A<1 \end{array}\right., \end{array}$$

where $E(Z(\hat{\rho }))=\frac{1}{2}ln(\frac{1+\rho }{1-\rho })$ , and $var(Z(\hat{\rho }))$ is substituted from equations (11) to (13) for the three different variance approximations, respectively.

### C.4 Normalized Searle method

In the normalized Searle method, the normalization is $\frac{1}{2}lnF(\hat{\rho })$ . Following the steps of the derivation of the assurance probability function for the Searle method, we have,

$$P(F(\hat{\rho })\leq f_{F-}^{-1}(\omega ))+P(F(\hat{\rho })>f_{F+}^{-1}(\omega ))\geq 1-\gamma .$$

We can take the logarithm to obtain,

$$P(\frac{1}{2}lnF(\hat{\rho })\leq \frac{1}{2}lnf_{F-}^{-1}(\omega ))+P(\frac{1}{2}lnF(\hat{\rho })>\frac{1}{2}lnf_{F+}^{-1}(\omega ))\geq 1-\gamma .\;$$

Now, since

$$P(\frac{1}{2}lnF(\hat{\rho })\leq \frac{1}{2}lnf_{F-}^{-1}(\omega ))=P(\frac{\frac{1}{2}lnF(\rho )-\frac{1}{2}lnF(\hat{\rho })}{\sqrt{var(Z(F(\hat{\rho })))}}\geq \frac{\frac{1}{2}lnF(\rho )-\frac{1}{2}lnf_{F-}^{-1}(\omega )}{\sqrt{var(Z(F(\hat{\rho })))}}),$$

and

$$P(\frac{1}{2}lnF(\hat{\rho })>\frac{1}{2}lnf_{F+}^{-1}(\omega ))=P(\frac{\frac{1}{2}lnF(\rho )-\frac{1}{2}lnF(\hat{\rho })}{\sqrt{var(Z(F(\hat{\rho })))}}\leq \frac{\frac{1}{2}lnF(\rho )-\frac{1}{2}lnf_{F+}^{-1}(\omega )}{\sqrt{var(Z(F(\hat{\rho })))}}),$$

the assurance probability function can be approximated by,

$$1+\Phi_{Z}(\frac{\frac{1}{2}lnF(\rho )-\frac{1}{2}lnf_{F+}^{-1}(\omega )}{\sqrt{var(Z(F(\hat{\rho })))}})-\Phi_{Z}(\frac{\frac{1}{2}lnF(\rho )-\frac{1}{2}lnf_{F-}^{-1}(\omega )}{\sqrt{var(Z(F(\hat{\rho })))}}),$$

where $var(Z(F(\hat{\rho })))=\frac{1}{2}(\frac{1}{n-1}+\frac{1}{n(k-1)})$ .

## Appendix D. Power functions

The power functions for the testing approach are derived here. The criterion for this approach is given as,

$$P(L\geq \rho_{0}|\rho =\rho_{A})\geq 1-\beta .$$

where L is the lower limit of the confidence interval for ρ .

### D.1 Wald confidence interval method

The lower limit of the confidence interval is given as $\hat{\rho }-z_{1-\alpha }\sqrt{var(\hat{\rho })}$ . Then, assuming $var(\hat{\rho })$ to be known, the criterion can be elaborated as,

$$\begin{array}{rl} & P(\hat{\rho }-z_{1-\alpha }\sqrt{var(\hat{\rho })}\geq \rho_{0}|\rho =\rho_{A})\geq 1-\beta ,\\ & \;\;⟺\;P(\hat{\rho }\geq \rho_{0}+z_{1-\alpha }\sqrt{var(\hat{\rho })}|\rho =\rho_{A})\approx P(\hat{\rho }\geq \rho_{0}+z_{1-\alpha }\sqrt{var(\hat{\rho }|\rho =\rho_{A})})\geq 1-\beta , \end{array}$$

so that,

$$\begin{array}{r} P(\frac{\rho_{A}-\hat{\rho }}{\sqrt{var(\hat{\rho }|\rho =\rho_{A})}}\leq \frac{\rho_{A}-\rho_{0}}{\sqrt{var(\hat{\rho }|\rho =\rho_{A})}}-z_{1-\alpha }|\rho =\rho_{A})\geq 1-\beta . \end{array}$$

Then, the power function can be written as,

$$\Phi_{z}(\frac{\rho_{A}-\rho_{0}}{\sqrt{var(\hat{\rho }|\rho =\rho_{A})}}-z_{1-\alpha }).$$

where $\Phi_{z}(.)$ is the cumulative standard normal distribution.

### D.2 Searle method

Following Shieh,^21^ the power function can be written as,

$$1-\Phi_{F}(\frac{\tau_{0}}{\tau_{A}}F_{n-1,n(k-1),1-\alpha }),$$

where $\Phi_{F}(.)$ is the cumulative *F*-distribution with n−1 and n(k−1) degrees of freedom, and $\tau_{i}=1+\frac{k\rho_{i}}{1-\rho_{i}}$ with i∈{0,A} representing values under the null ( 0 ) and alternative hypotheses ( A ).

### D.3 Normalized ICC method

The lower limit of the confidence interval is given as $\frac{exp(2L_{Z})-1}{exp(2L_{Z})+1}$ . Then the criterion can be approximated by,

$$\begin{array}{rl} & P(\frac{exp(2L_{Z})-1}{exp(2L_{Z})+1}\geq \rho_{0}|\rho =\rho_{A})\geq 1-\beta ,\\ & \;\;⟺\;P(L_{Z}\geq \frac{1}{2}ln\frac{1+\rho_{0}}{1-\rho_{0}}|\rho =\rho_{A})\geq 1-\beta . \end{array}$$

Following the same steps as for the Wald confidence interval method, we get,

$$1-\Phi_{z}(z_{1-\alpha }-\frac{\mu_{zA}^{*}-\mu_{z0}^{*}}{\sqrt{var(Z(\hat{\rho })|\rho =\rho_{A})}}),$$

where $\mu_{zi}^{*}=\frac{1}{2}ln\tau_{i}^{*}$ , and $\tau_{i}^{*}=\frac{1+\rho_{i}}{1-\rho_{i}}$ with i∈{0,A} representing values under the null and alternative hypotheses.

### D.4 Normalized Searle method

The lower limit of the confidence interval is given as $\frac{exp(2L_{ZF})-1}{exp(2L_{ZF})+k-1}$ . Then the criterion can be approximated by,

$$\begin{array}{rl} & P(\frac{exp(2L_{ZF})-1}{exp(2L_{ZF})+k-1}\geq \rho_{0}|\rho =\rho_{A})\geq 1-\beta ,\\ & \;\;⟺\;P(L_{ZF}\geq \frac{1}{2}ln\frac{1+(k-1)\rho_{0}}{1-\rho_{0}}|\rho =\rho_{A})\geq 1-\beta . \end{array}$$

Then, following the same steps as we did for the Wald confidence interval method, we get,

(.1)
$$1-\Phi_{z}(z_{1-\alpha }-\frac{\mu_{zA}^{*}-\mu_{z0}^{*}}{\sqrt{var(Z(\hat{\rho })|\rho =\rho_{A})}}),$$

where $\mu_{zi}=\frac{1}{2}ln\tau_{i}$ , with i∈{0,A} representing values under the null and alternative hypotheses.

## Appendix E. Sample size formulas used in common statistical software

An overview of the methods implemented in common statistical software is provided in Table A.1.

**Table 8.** A list of other relevant software commonly used to obtain sample size for ICC(1).

Source	Sample size approaches	Additional comments
SAS^19,21^	Testing ( $ZF_{\rho }$ )	Shieh provided sample size calculation for assurance probability ( $Wald_{F}$ , F ) and testing ( F , $ZF_{\rho }$ ) also including cost-constraints
PASS^20^	Testing ( $ZF_{\rho }$ )	
*R package* MBESS^46^	Assurance probability ( $Wald_{F}$ ) Testing ( $ZF_{\rho }$ )	Allows sample size calculation under cost-constraints
*R package* presize^47^	Assurance probability ( $Wald_{F}$ ) Testing ( $ZF_{\rho }$ )	Allows sample size calculation with dropout rates. (webcalculator)
*R package* ICC.Sample.Size^48^	Testing ( $ZF_{\rho }$ )	Allows sample size calculation for the testing approach with two tails

ICC: intraclass correlation coefficient; SAS: Statistical Analysis System.
