# Supplementary material for: Review of sample size determination methods for the intraclass correlation coefficient in the one-way analysis of variance model
Source: Stat Methods Med Res. 2024 Feb 6;33(3):532–53. doi: 10.1177/09622802231224657 (PMC10981208; doi:10.1177/09622802231224657)
Supplement: sj-pdf-1-smm-10.1177_09622802231224657 - Supplemental material for Review of sample size determination methods for the intraclass correlation coefficient in the one-way analysis of variance model [file sj-pdf-1-smm-10.1177_09622802231224657.pdf]

Supplementary Tables

0.1 Supplementary Table 1

The table shows the coverage probability obtained from 25,000 simulations for the eight confidence interval methods under different scenarios mentioned in Section 3 of the main paper.

ρ	k	n	$E[\hat{\rho}]$	$Wald_S$	$Wald_F$	$Wald_{Ze}$	F	Z_S	Z_F	Z_{Ze}	ZF_ρ
0.1	2	20	0.095	0.914	0.912	0.953	0.949	0.943	0.940	0.978	0.943
0.2	2	20	0.190	0.916	0.913	0.955	0.951	0.945	0.942	0.978	0.945
0.3	2	20	0.286	0.917	0.915	0.957	0.952	0.945	0.943	0.978	0.945
0.4	2	20	0.383	0.919	0.916	0.956	0.952	0.946	0.942	0.979	0.946
0.5	2	20	0.481	0.920	0.917	0.955	0.953	0.946	0.943	0.979	0.946
0.6	2	20	0.580	0.920	0.917	0.954	0.953	0.946	0.943	0.979	0.946
0.7	2	20	0.681	0.920	0.917	0.952	0.953	0.946	0.943	0.979	0.946
0.8	2	20	0.784	0.920	0.918	0.950	0.952	0.946	0.943	0.979	0.946
0.9	2	20	0.890	0.920	0.918	0.947	0.952	0.946	0.942	0.979	0.946
0.1	3	20	0.095	0.922	0.917	0.940	0.950	0.931	0.927	0.948	0.944
0.2	3	20	0.192	0.921	0.918	0.941	0.950	0.932	0.928	0.950	0.944
0.3	3	20	0.288	0.924	0.919	0.943	0.950	0.933	0.929	0.952	0.944
0.4	3	20	0.385	0.925	0.920	0.944	0.950	0.935	0.931	0.954	0.944
0.5	3	20	0.483	0.927	0.923	0.945	0.949	0.938	0.934	0.956	0.943
0.6	3	20	0.581	0.928	0.925	0.947	0.949	0.939	0.935	0.957	0.943
0.7	3	20	0.682	0.931	0.928	0.948	0.950	0.941	0.937	0.959	0.944
0.8	3	20	0.785	0.934	0.931	0.950	0.950	0.942	0.938	0.960	0.944
0.9	3	20	0.891	0.938	0.935	0.952	0.951	0.942	0.938	0.961	0.944
0.1	4	20	0.097	0.921	0.917	0.933	0.951	0.926	0.922	0.937	0.945
0.2	4	20	0.193	0.922	0.917	0.934	0.952	0.928	0.923	0.939	0.945
0.3	4	20	0.290	0.924	0.920	0.937	0.952	0.931	0.925	0.942	0.944
0.4	4	20	0.386	0.927	0.922	0.941	0.953	0.932	0.927	0.944	0.945
0.5	4	20	0.484	0.930	0.925	0.943	0.953	0.934	0.929	0.946	0.944
0.6	4	20	0.582	0.933	0.928	0.946	0.953	0.937	0.933	0.949	0.945
0.7	4	20	0.683	0.936	0.932	0.948	0.952	0.939	0.935	0.951	0.945
0.8	4	20	0.785	0.940	0.934	0.950	0.952	0.940	0.936	0.953	0.944
0.9	4	20	0.891	0.943	0.939	0.953	0.950	0.942	0.938	0.955	0.944
0.1	5	20	0.097	0.918	0.914	0.927	0.953	0.921	0.916	0.930	0.945
0.2	5	20	0.194	0.922	0.917	0.930	0.953	0.925	0.920	0.933	0.943
0.3	5	20	0.291	0.924	0.919	0.933	0.953	0.928	0.923	0.936	0.944
0.4	5	20	0.387	0.927	0.922	0.936	0.953	0.931	0.926	0.939	0.944
0.5	5	20	0.485	0.929	0.924	0.939	0.953	0.932	0.928	0.941	0.945

0.6	5	20	0.583	0.933	0.927	0.943	0.952	0.935	0.930	0.944	0.945
0.7	5	20	0.683	0.936	0.931	0.946	0.952	0.938	0.933	0.947	0.945
0.8	5	20	0.785	0.941	0.935	0.949	0.952	0.940	0.935	0.949	0.944
0.9	5	20	0.891	0.944	0.939	0.951	0.952	0.942	0.936	0.951	0.944
0.1	6	20	0.098	0.920	0.914	0.926	0.952	0.922	0.917	0.927	0.945
0.2	6	20	0.195	0.922	0.917	0.929	0.951	0.924	0.920	0.932	0.944
0.3	6	20	0.291	0.926	0.921	0.933	0.952	0.929	0.924	0.935	0.945
0.4	6	20	0.388	0.927	0.921	0.935	0.951	0.931	0.926	0.937	0.946
0.5	6	20	0.485	0.930	0.924	0.939	0.952	0.933	0.929	0.941	0.946
0.6	6	20	0.584	0.932	0.927	0.940	0.951	0.936	0.931	0.944	0.945
0.7	6	20	0.684	0.935	0.930	0.943	0.951	0.938	0.932	0.944	0.943
0.8	6	20	0.786	0.939	0.934	0.947	0.951	0.939	0.934	0.946	0.943
0.9	6	20	0.891	0.944	0.939	0.950	0.950	0.941	0.935	0.948	0.943
0.1	7	20	0.098	0.920	0.916	0.926	0.952	0.922	0.917	0.927	0.945
0.2	7	20	0.195	0.922	0.917	0.928	0.951	0.925	0.919	0.930	0.944
0.3	7	20	0.292	0.925	0.919	0.930	0.951	0.927	0.922	0.932	0.944
0.4	7	20	0.389	0.926	0.921	0.933	0.952	0.929	0.924	0.935	0.945
0.5	7	20	0.486	0.930	0.925	0.936	0.952	0.932	0.926	0.938	0.944
0.6	7	20	0.584	0.932	0.927	0.939	0.952	0.934	0.929	0.941	0.944
0.7	7	20	0.684	0.936	0.930	0.942	0.952	0.936	0.931	0.943	0.943
0.8	7	20	0.786	0.941	0.935	0.946	0.951	0.938	0.933	0.944	0.943
0.9	7	20	0.891	0.945	0.941	0.950	0.949	0.940	0.935	0.947	0.943
0.1	8	20	0.098	0.918	0.913	0.923	0.952	0.919	0.914	0.924	0.943
0.2	8	20	0.195	0.920	0.916	0.925	0.950	0.922	0.917	0.926	0.943
0.3	8	20	0.292	0.923	0.916	0.928	0.951	0.924	0.919	0.928	0.943
0.4	8	20	0.389	0.925	0.919	0.930	0.951	0.927	0.922	0.931	0.943
0.5	8	20	0.486	0.929	0.923	0.934	0.951	0.929	0.924	0.935	0.943
0.6	8	20	0.584	0.931	0.926	0.937	0.950	0.932	0.928	0.938	0.943
0.7	8	20	0.684	0.935	0.929	0.940	0.950	0.936	0.931	0.941	0.942
0.8	8	20	0.786	0.940	0.935	0.945	0.949	0.938	0.933	0.943	0.944
0.9	8	20	0.891	0.946	0.942	0.951	0.948	0.941	0.935	0.946	0.944
0.1	9	20	0.098	0.916	0.911	0.920	0.952	0.917	0.912	0.921	0.943
0.2	9	20	0.196	0.919	0.913	0.923	0.952	0.920	0.915	0.924	0.942
0.3	9	20	0.292	0.922	0.916	0.926	0.951	0.923	0.919	0.928	0.943
0.4	9	20	0.389	0.925	0.919	0.930	0.949	0.927	0.921	0.931	0.943
0.5	9	20	0.486	0.929	0.923	0.934	0.950	0.930	0.925	0.935	0.943
0.6	9	20	0.584	0.932	0.926	0.937	0.950	0.933	0.928	0.937	0.943
0.7	9	20	0.684	0.934	0.929	0.939	0.950	0.935	0.930	0.940	0.942
0.8	9	20	0.786	0.940	0.934	0.944	0.949	0.938	0.932	0.942	0.942
0.9	9	20	0.891	0.946	0.941	0.951	0.950	0.940	0.935	0.944	0.942
0.1	10	20	0.098	0.917	0.912	0.920	0.950	0.917	0.912	0.920	0.942
0.2	10	20	0.196	0.920	0.916	0.924	0.953	0.921	0.916	0.925	0.944
0.3	10	20	0.292	0.923	0.917	0.927	0.952	0.924	0.920	0.928	0.944
0.4	10	20	0.389	0.925	0.920	0.930	0.951	0.928	0.922	0.931	0.945
0.5	10	20	0.486	0.929	0.923	0.934	0.950	0.929	0.924	0.933	0.944
0.6	10	20	0.584	0.931	0.926	0.936	0.950	0.933	0.928	0.937	0.943
0.7	10	20	0.684	0.935	0.930	0.940	0.950	0.935	0.930	0.939	0.943
0.8	10	20	0.786	0.941	0.935	0.944	0.950	0.937	0.933	0.941	0.943
0.9	10	20	0.891	0.947	0.941	0.950	0.950	0.939	0.935	0.943	0.942
0.1	2	30	0.096	0.928	0.926	0.953	0.951	0.947	0.945	0.970	0.947

0.2	2	30	0.193	0.928	0.926	0.952	0.950	0.945	0.943	0.970	0.945
0.3	2	30	0.291	0.927	0.926	0.953	0.949	0.945	0.943	0.971	0.945
0.4	2	30	0.389	0.926	0.924	0.953	0.950	0.946	0.944	0.970	0.946
0.5	2	30	0.487	0.926	0.924	0.952	0.951	0.946	0.944	0.970	0.946
0.6	2	30	0.587	0.928	0.926	0.952	0.951	0.947	0.945	0.970	0.947
0.7	2	30	0.688	0.930	0.928	0.952	0.950	0.947	0.945	0.970	0.947
0.8	2	30	0.790	0.930	0.928	0.952	0.951	0.947	0.945	0.970	0.947
0.9	2	30	0.894	0.931	0.930	0.951	0.951	0.947	0.945	0.971	0.947
0.1	3	30	0.097	0.931	0.928	0.944	0.952	0.937	0.935	0.950	0.947
0.2	3	30	0.195	0.931	0.929	0.945	0.950	0.939	0.937	0.952	0.947
0.3	3	30	0.292	0.933	0.930	0.946	0.951	0.942	0.938	0.953	0.947
0.4	3	30	0.390	0.934	0.931	0.948	0.951	0.941	0.939	0.953	0.946
0.5	3	30	0.489	0.935	0.933	0.948	0.951	0.942	0.940	0.955	0.946
0.6	3	30	0.588	0.937	0.934	0.950	0.951	0.943	0.941	0.957	0.947
0.7	3	30	0.688	0.938	0.935	0.951	0.951	0.945	0.942	0.958	0.948
0.8	3	30	0.790	0.939	0.937	0.951	0.950	0.947	0.944	0.957	0.948
0.9	3	30	0.894	0.940	0.937	0.951	0.951	0.945	0.943	0.957	0.946
0.1	4	30	0.098	0.930	0.927	0.938	0.951	0.933	0.931	0.941	0.946
0.2	4	30	0.196	0.931	0.929	0.940	0.950	0.935	0.933	0.943	0.946
0.3	4	30	0.294	0.932	0.929	0.941	0.951	0.937	0.934	0.945	0.947
0.4	4	30	0.392	0.934	0.932	0.943	0.950	0.938	0.935	0.946	0.946
0.5	4	30	0.490	0.936	0.933	0.944	0.950	0.940	0.937	0.948	0.946
0.6	4	30	0.589	0.938	0.935	0.946	0.950	0.942	0.939	0.950	0.946
0.7	4	30	0.689	0.940	0.936	0.948	0.950	0.942	0.940	0.950	0.945
0.8	4	30	0.791	0.943	0.940	0.950	0.950	0.943	0.940	0.951	0.945
0.9	4	30	0.894	0.945	0.943	0.952	0.949	0.944	0.941	0.953	0.945
0.1	5	30	0.099	0.930	0.927	0.936	0.951	0.931	0.929	0.937	0.946
0.2	5	30	0.197	0.931	0.928	0.938	0.950	0.934	0.931	0.940	0.946
0.3	5	30	0.294	0.933	0.930	0.939	0.950	0.936	0.933	0.941	0.946
0.4	5	30	0.392	0.935	0.931	0.941	0.952	0.936	0.932	0.942	0.946
0.5	5	30	0.490	0.937	0.934	0.945	0.951	0.938	0.934	0.944	0.946
0.6	5	30	0.589	0.938	0.935	0.945	0.951	0.939	0.937	0.945	0.945
0.7	5	30	0.689	0.940	0.937	0.947	0.950	0.941	0.937	0.947	0.945
0.8	5	30	0.791	0.943	0.939	0.949	0.949	0.942	0.938	0.947	0.944
0.9	5	30	0.894	0.946	0.943	0.951	0.949	0.944	0.940	0.950	0.945
0.1	6	30	0.099	0.929	0.926	0.934	0.951	0.931	0.928	0.935	0.945
0.2	6	30	0.197	0.929	0.926	0.934	0.952	0.931	0.928	0.936	0.946
0.3	6	30	0.295	0.932	0.929	0.938	0.951	0.934	0.931	0.939	0.946
0.4	6	30	0.393	0.933	0.929	0.938	0.951	0.935	0.932	0.940	0.945
0.5	6	30	0.491	0.934	0.931	0.940	0.950	0.936	0.933	0.941	0.945
0.6	6	30	0.590	0.937	0.934	0.942	0.950	0.938	0.935	0.942	0.944
0.7	6	30	0.690	0.940	0.937	0.945	0.950	0.940	0.937	0.945	0.944
0.8	6	30	0.791	0.944	0.940	0.948	0.949	0.942	0.938	0.946	0.944
0.9	6	30	0.894	0.946	0.942	0.950	0.949	0.943	0.940	0.947	0.944
0.1	7	30	0.099	0.926	0.924	0.931	0.950	0.928	0.925	0.931	0.944
0.2	7	30	0.197	0.928	0.924	0.932	0.950	0.930	0.926	0.933	0.945
0.3	7	30	0.295	0.930	0.926	0.934	0.951	0.932	0.928	0.935	0.945
0.4	7	30	0.393	0.933	0.929	0.937	0.950	0.934	0.931	0.938	0.945
0.5	7	30	0.491	0.935	0.932	0.939	0.949	0.936	0.933	0.940	0.945
0.6	7	30	0.590	0.937	0.933	0.940	0.948	0.939	0.935	0.942	0.944

0.7	7	30	0.690	0.939	0.936	0.943	0.948	0.940	0.937	0.944	0.945
0.8	7	30	0.791	0.943	0.940	0.947	0.948	0.942	0.938	0.946	0.944
0.9	7	30	0.894	0.946	0.943	0.950	0.949	0.942	0.939	0.946	0.944
0.1	8	30	0.099	0.927	0.923	0.930	0.949	0.928	0.924	0.931	0.944
0.2	8	30	0.197	0.929	0.925	0.932	0.949	0.930	0.927	0.933	0.944
0.3	8	30	0.295	0.930	0.927	0.934	0.949	0.931	0.928	0.935	0.944
0.4	8	30	0.393	0.932	0.928	0.935	0.950	0.933	0.929	0.936	0.944
0.5	8	30	0.491	0.934	0.931	0.938	0.950	0.935	0.932	0.938	0.943
0.6	8	30	0.590	0.938	0.934	0.941	0.949	0.936	0.934	0.940	0.944
0.7	8	30	0.690	0.941	0.937	0.944	0.949	0.939	0.936	0.942	0.944
0.8	8	30	0.791	0.944	0.941	0.947	0.949	0.941	0.938	0.945	0.945
0.9	8	30	0.894	0.947	0.944	0.950	0.949	0.942	0.940	0.946	0.944
0.1	9	30	0.099	0.926	0.923	0.929	0.948	0.927	0.924	0.929	0.943
0.2	9	30	0.197	0.929	0.926	0.931	0.949	0.929	0.926	0.932	0.944
0.3	9	30	0.295	0.931	0.927	0.934	0.949	0.932	0.929	0.935	0.944
0.4	9	30	0.393	0.932	0.929	0.935	0.949	0.934	0.930	0.937	0.944
0.5	9	30	0.491	0.935	0.931	0.938	0.950	0.935	0.932	0.939	0.944
0.6	9	30	0.590	0.938	0.935	0.941	0.949	0.938	0.934	0.940	0.944
0.7	9	30	0.690	0.941	0.937	0.944	0.949	0.940	0.936	0.942	0.944
0.8	9	30	0.791	0.944	0.940	0.947	0.950	0.941	0.937	0.944	0.944
0.9	9	30	0.894	0.948	0.944	0.950	0.950	0.942	0.939	0.945	0.943
0.1	10	30	0.099	0.924	0.921	0.927	0.949	0.925	0.921	0.927	0.943
0.2	10	30	0.197	0.927	0.924	0.930	0.948	0.928	0.925	0.930	0.944
0.3	10	30	0.295	0.929	0.925	0.932	0.948	0.930	0.927	0.932	0.944
0.4	10	30	0.393	0.930	0.927	0.933	0.949	0.931	0.927	0.934	0.943
0.5	10	30	0.491	0.934	0.930	0.936	0.950	0.933	0.930	0.936	0.943
0.6	10	30	0.590	0.937	0.933	0.940	0.950	0.937	0.933	0.939	0.942
0.7	10	30	0.690	0.941	0.937	0.943	0.949	0.938	0.935	0.940	0.944
0.8	10	30	0.791	0.944	0.940	0.947	0.949	0.939	0.936	0.943	0.943
0.9	10	30	0.894	0.948	0.944	0.951	0.949	0.942	0.938	0.944	0.943
0.1	2	40	0.097	0.935	0.933	0.953	0.951	0.948	0.947	0.967	0.948
0.2	2	40	0.195	0.935	0.934	0.954	0.952	0.949	0.948	0.967	0.949
0.3	2	40	0.293	0.935	0.933	0.954	0.951	0.948	0.947	0.967	0.948
0.4	2	40	0.391	0.935	0.934	0.953	0.951	0.948	0.947	0.967	0.948
0.5	2	40	0.490	0.934	0.933	0.953	0.951	0.947	0.946	0.967	0.947
0.6	2	40	0.590	0.935	0.934	0.953	0.952	0.949	0.947	0.967	0.949
0.7	2	40	0.691	0.935	0.934	0.952	0.952	0.949	0.947	0.967	0.949
0.8	2	40	0.793	0.935	0.934	0.952	0.952	0.949	0.947	0.967	0.949
0.9	2	40	0.896	0.935	0.934	0.951	0.952	0.949	0.947	0.968	0.949
0.1	3	40	0.098	0.935	0.934	0.945	0.951	0.941	0.939	0.949	0.947
0.2	3	40	0.196	0.938	0.936	0.947	0.951	0.943	0.941	0.951	0.948
0.3	3	40	0.294	0.937	0.935	0.949	0.952	0.943	0.941	0.951	0.949
0.4	3	40	0.393	0.938	0.936	0.948	0.950	0.944	0.943	0.953	0.948
0.5	3	40	0.492	0.939	0.937	0.948	0.950	0.945	0.942	0.954	0.947
0.6	3	40	0.591	0.940	0.938	0.950	0.951	0.945	0.943	0.955	0.947
0.7	3	40	0.692	0.941	0.940	0.950	0.951	0.946	0.944	0.955	0.948
0.8	3	40	0.793	0.942	0.940	0.951	0.951	0.946	0.945	0.956	0.948
0.9	3	40	0.896	0.943	0.942	0.951	0.951	0.947	0.945	0.957	0.948
0.1	4	40	0.098	0.936	0.935	0.942	0.951	0.938	0.936	0.944	0.949
0.2	4	40	0.196	0.937	0.934	0.943	0.951	0.940	0.937	0.945	0.947

0.3	4	40	0.295	0.937	0.935	0.942	0.951	0.940	0.938	0.945	0.946
0.4	4	40	0.393	0.937	0.935	0.943	0.950	0.941	0.939	0.945	0.945
0.5	4	40	0.492	0.940	0.938	0.946	0.950	0.941	0.939	0.947	0.946
0.6	4	40	0.591	0.942	0.940	0.948	0.951	0.943	0.941	0.949	0.947
0.7	4	40	0.692	0.943	0.940	0.949	0.951	0.945	0.942	0.951	0.948
0.8	4	40	0.793	0.945	0.942	0.950	0.951	0.947	0.944	0.952	0.948
0.9	4	40	0.896	0.946	0.944	0.952	0.951	0.947	0.945	0.953	0.948
0.1	5	40	0.098	0.932	0.930	0.937	0.949	0.934	0.931	0.939	0.945
0.2	5	40	0.197	0.935	0.932	0.939	0.949	0.937	0.934	0.942	0.945
0.3	5	40	0.295	0.936	0.934	0.940	0.950	0.938	0.936	0.943	0.946
0.4	5	40	0.393	0.938	0.935	0.943	0.951	0.940	0.938	0.944	0.947
0.5	5	40	0.492	0.941	0.938	0.945	0.951	0.942	0.939	0.946	0.947
0.6	5	40	0.592	0.942	0.940	0.947	0.951	0.943	0.941	0.948	0.948
0.7	5	40	0.692	0.944	0.942	0.949	0.951	0.943	0.941	0.948	0.947
0.8	5	40	0.793	0.947	0.945	0.951	0.951	0.945	0.943	0.949	0.947
0.9	5	40	0.896	0.949	0.948	0.953	0.951	0.946	0.943	0.951	0.947
0.1	6	40	0.099	0.932	0.930	0.936	0.950	0.934	0.931	0.937	0.946
0.2	6	40	0.197	0.937	0.934	0.940	0.951	0.938	0.935	0.942	0.948
0.3	6	40	0.296	0.937	0.935	0.941	0.952	0.940	0.937	0.942	0.947
0.4	6	40	0.394	0.940	0.938	0.943	0.951	0.941	0.938	0.944	0.948
0.5	6	40	0.493	0.942	0.939	0.945	0.952	0.942	0.940	0.946	0.948
0.6	6	40	0.592	0.943	0.940	0.946	0.952	0.943	0.941	0.946	0.948
0.7	6	40	0.692	0.944	0.942	0.948	0.951	0.944	0.942	0.948	0.948
0.8	6	40	0.793	0.947	0.945	0.950	0.951	0.946	0.944	0.949	0.949
0.9	6	40	0.896	0.949	0.947	0.952	0.952	0.946	0.944	0.950	0.947
0.1	7	40	0.099	0.932	0.929	0.935	0.951	0.933	0.930	0.936	0.947
0.2	7	40	0.197	0.934	0.931	0.937	0.951	0.935	0.932	0.938	0.947
0.3	7	40	0.296	0.935	0.932	0.938	0.950	0.937	0.935	0.940	0.947
0.4	7	40	0.394	0.937	0.935	0.940	0.950	0.939	0.937	0.942	0.946
0.5	7	40	0.493	0.939	0.937	0.943	0.951	0.940	0.939	0.943	0.946
0.6	7	40	0.592	0.942	0.940	0.945	0.951	0.942	0.939	0.945	0.947
0.7	7	40	0.692	0.945	0.943	0.948	0.951	0.944	0.941	0.947	0.947
0.8	7	40	0.793	0.947	0.945	0.950	0.951	0.945	0.943	0.948	0.947
0.9	7	40	0.896	0.950	0.947	0.953	0.952	0.947	0.944	0.949	0.948
0.1	8	40	0.099	0.932	0.930	0.935	0.949	0.932	0.930	0.935	0.945
0.2	8	40	0.197	0.933	0.931	0.936	0.949	0.934	0.931	0.937	0.945
0.3	8	40	0.296	0.936	0.933	0.938	0.949	0.937	0.934	0.939	0.945
0.4	8	40	0.394	0.938	0.935	0.940	0.950	0.938	0.935	0.940	0.946
0.5	8	40	0.493	0.939	0.937	0.942	0.950	0.940	0.938	0.942	0.946
0.6	8	40	0.592	0.942	0.939	0.945	0.951	0.941	0.939	0.943	0.946
0.7	8	40	0.692	0.945	0.943	0.947	0.951	0.942	0.940	0.945	0.947
0.8	8	40	0.793	0.948	0.946	0.950	0.952	0.944	0.942	0.947	0.946
0.9	8	40	0.896	0.951	0.949	0.953	0.951	0.945	0.942	0.948	0.946
0.1	9	40	0.099	0.930	0.928	0.932	0.950	0.931	0.928	0.933	0.945
0.2	9	40	0.198	0.933	0.931	0.936	0.951	0.934	0.931	0.936	0.946
0.3	9	40	0.296	0.936	0.934	0.938	0.949	0.937	0.934	0.939	0.947
0.4	9	40	0.394	0.938	0.935	0.940	0.949	0.938	0.936	0.940	0.947
0.5	9	40	0.493	0.940	0.937	0.942	0.950	0.940	0.938	0.943	0.947
0.6	9	40	0.592	0.942	0.939	0.945	0.952	0.942	0.939	0.944	0.947
0.7	9	40	0.692	0.946	0.943	0.948	0.952	0.942	0.940	0.945	0.947

0.8	9	40	0.793	0.948	0.946	0.950	0.952	0.943	0.941	0.946	0.947
0.9	9	40	0.896	0.951	0.948	0.952	0.952	0.945	0.943	0.947	0.947
0.1	10	40	0.099	0.932	0.929	0.933	0.951	0.932	0.930	0.934	0.947
0.2	10	40	0.198	0.935	0.932	0.937	0.950	0.935	0.933	0.937	0.947
0.3	10	40	0.296	0.936	0.933	0.938	0.951	0.936	0.934	0.938	0.946
0.4	10	40	0.394	0.938	0.935	0.939	0.951	0.938	0.935	0.940	0.946
0.5	10	40	0.493	0.939	0.937	0.941	0.951	0.940	0.937	0.942	0.946
0.6	10	40	0.592	0.942	0.939	0.944	0.951	0.941	0.938	0.943	0.947
0.7	10	40	0.692	0.946	0.943	0.948	0.952	0.943	0.940	0.944	0.947
0.8	10	40	0.793	0.948	0.946	0.950	0.952	0.944	0.942	0.946	0.947
0.9	10	40	0.896	0.951	0.949	0.953	0.952	0.945	0.942	0.947	0.947
0.1	2	50	0.098	0.935	0.934	0.951	0.950	0.947	0.946	0.963	0.947
0.2	2	50	0.196	0.935	0.934	0.951	0.950	0.948	0.946	0.963	0.948
0.3	2	50	0.294	0.935	0.934	0.951	0.950	0.947	0.945	0.964	0.947
0.4	2	50	0.393	0.936	0.935	0.951	0.950	0.947	0.946	0.963	0.947
0.5	2	50	0.492	0.937	0.935	0.952	0.949	0.947	0.946	0.962	0.947
0.6	2	50	0.592	0.938	0.937	0.952	0.950	0.947	0.946	0.961	0.947
0.7	2	50	0.693	0.938	0.936	0.952	0.950	0.948	0.947	0.962	0.948
0.8	2	50	0.794	0.938	0.937	0.951	0.950	0.948	0.947	0.962	0.948
0.9	2	50	0.896	0.937	0.937	0.950	0.950	0.948	0.947	0.963	0.948
0.1	3	50	0.099	0.938	0.936	0.946	0.950	0.942	0.941	0.950	0.947
0.2	3	50	0.197	0.938	0.936	0.946	0.949	0.942	0.940	0.949	0.947
0.3	3	50	0.296	0.939	0.937	0.946	0.950	0.942	0.941	0.950	0.947
0.4	3	50	0.394	0.939	0.937	0.948	0.950	0.943	0.942	0.951	0.947
0.5	3	50	0.493	0.941	0.939	0.948	0.950	0.945	0.943	0.953	0.948
0.6	3	50	0.593	0.942	0.941	0.950	0.950	0.946	0.944	0.953	0.947
0.7	3	50	0.693	0.943	0.942	0.950	0.951	0.946	0.945	0.954	0.948
0.8	3	50	0.794	0.944	0.942	0.951	0.950	0.948	0.946	0.955	0.949
0.9	3	50	0.897	0.946	0.944	0.952	0.950	0.948	0.946	0.955	0.948
0.1	4	50	0.099	0.939	0.937	0.944	0.950	0.941	0.939	0.946	0.948
0.2	4	50	0.197	0.940	0.938	0.944	0.951	0.942	0.940	0.947	0.948
0.3	4	50	0.296	0.941	0.939	0.945	0.951	0.943	0.941	0.949	0.948
0.4	4	50	0.395	0.943	0.941	0.947	0.951	0.945	0.943	0.950	0.949
0.5	4	50	0.494	0.943	0.941	0.948	0.951	0.945	0.944	0.951	0.949
0.6	4	50	0.593	0.944	0.942	0.948	0.951	0.945	0.944	0.950	0.948
0.7	4	50	0.693	0.944	0.942	0.949	0.950	0.945	0.944	0.951	0.948
0.8	4	50	0.794	0.945	0.944	0.950	0.950	0.947	0.945	0.951	0.947
0.9	4	50	0.897	0.947	0.946	0.951	0.950	0.947	0.945	0.951	0.948
0.1	5	50	0.099	0.937	0.936	0.941	0.951	0.939	0.937	0.942	0.947
0.2	5	50	0.198	0.939	0.937	0.942	0.949	0.941	0.939	0.944	0.947
0.3	5	50	0.296	0.939	0.937	0.943	0.950	0.942	0.940	0.945	0.948
0.4	5	50	0.395	0.941	0.939	0.945	0.951	0.942	0.940	0.946	0.948
0.5	5	50	0.494	0.942	0.940	0.945	0.950	0.944	0.942	0.946	0.948
0.6	5	50	0.593	0.943	0.941	0.947	0.950	0.944	0.942	0.948	0.948
0.7	5	50	0.694	0.943	0.941	0.947	0.950	0.945	0.943	0.948	0.947
0.8	5	50	0.795	0.946	0.944	0.949	0.949	0.945	0.944	0.949	0.947
0.9	5	50	0.897	0.948	0.946	0.951	0.950	0.946	0.945	0.950	0.947
0.1	6	50	0.099	0.937	0.935	0.939	0.950	0.938	0.936	0.941	0.947
0.2	6	50	0.198	0.937	0.935	0.940	0.950	0.938	0.936	0.941	0.946
0.3	6	50	0.297	0.939	0.938	0.942	0.950	0.942	0.940	0.945	0.947

0.4	6	50	0.395	0.940	0.938	0.944	0.949	0.941	0.939	0.944	0.947
0.5	6	50	0.494	0.941	0.939	0.944	0.949	0.942	0.940	0.944	0.947
0.6	6	50	0.594	0.941	0.940	0.945	0.948	0.943	0.941	0.946	0.946
0.7	6	50	0.694	0.943	0.941	0.945	0.947	0.945	0.943	0.947	0.947
0.8	6	50	0.795	0.945	0.943	0.948	0.948	0.945	0.943	0.947	0.947
0.9	6	50	0.897	0.948	0.946	0.950	0.949	0.946	0.943	0.949	0.947
0.1	7	50	0.099	0.935	0.932	0.937	0.948	0.935	0.934	0.938	0.945
0.2	7	50	0.198	0.935	0.933	0.938	0.949	0.936	0.934	0.938	0.944
0.3	7	50	0.297	0.938	0.935	0.939	0.948	0.937	0.935	0.940	0.946
0.4	7	50	0.395	0.938	0.936	0.941	0.949	0.939	0.937	0.941	0.946
0.5	7	50	0.494	0.940	0.938	0.943	0.948	0.941	0.939	0.944	0.945
0.6	7	50	0.594	0.942	0.940	0.944	0.949	0.943	0.940	0.945	0.946
0.7	7	50	0.694	0.943	0.941	0.946	0.948	0.944	0.942	0.946	0.946
0.8	7	50	0.795	0.945	0.944	0.947	0.949	0.944	0.943	0.946	0.946
0.9	7	50	0.897	0.949	0.947	0.951	0.950	0.945	0.943	0.947	0.946
0.1	8	50	0.099	0.934	0.932	0.936	0.949	0.934	0.932	0.936	0.947
0.2	8	50	0.198	0.936	0.934	0.938	0.950	0.936	0.934	0.938	0.947
0.3	8	50	0.297	0.937	0.935	0.939	0.950	0.938	0.936	0.940	0.947
0.4	8	50	0.395	0.939	0.937	0.941	0.950	0.939	0.937	0.941	0.946
0.5	8	50	0.494	0.941	0.939	0.943	0.949	0.941	0.939	0.943	0.946
0.6	8	50	0.594	0.943	0.941	0.945	0.950	0.943	0.941	0.945	0.946
0.7	8	50	0.694	0.946	0.943	0.948	0.951	0.944	0.942	0.946	0.946
0.8	8	50	0.795	0.947	0.945	0.949	0.951	0.944	0.942	0.946	0.947
0.9	8	50	0.897	0.950	0.948	0.952	0.950	0.946	0.943	0.947	0.946
0.1	9	50	0.099	0.934	0.932	0.936	0.949	0.934	0.932	0.936	0.946
0.2	9	50	0.198	0.933	0.932	0.935	0.950	0.934	0.932	0.935	0.947
0.3	9	50	0.297	0.937	0.935	0.939	0.950	0.937	0.935	0.938	0.948
0.4	9	50	0.396	0.940	0.938	0.942	0.950	0.940	0.937	0.942	0.947
0.5	9	50	0.494	0.941	0.939	0.943	0.950	0.941	0.939	0.943	0.945
0.6	9	50	0.594	0.943	0.940	0.945	0.950	0.942	0.940	0.944	0.946
0.7	9	50	0.694	0.946	0.943	0.947	0.950	0.943	0.941	0.945	0.946
0.8	9	50	0.795	0.947	0.945	0.949	0.950	0.944	0.942	0.946	0.946
0.9	9	50	0.897	0.949	0.948	0.951	0.950	0.946	0.944	0.948	0.947
0.1	10	50	0.099	0.935	0.933	0.936	0.950	0.935	0.934	0.937	0.947
0.2	10	50	0.198	0.937	0.935	0.938	0.950	0.937	0.935	0.939	0.947
0.3	10	50	0.297	0.938	0.936	0.940	0.950	0.939	0.936	0.940	0.948
0.4	10	50	0.396	0.940	0.938	0.941	0.951	0.940	0.937	0.941	0.946
0.5	10	50	0.494	0.942	0.940	0.943	0.950	0.940	0.938	0.941	0.947
0.6	10	50	0.594	0.943	0.942	0.945	0.950	0.942	0.940	0.944	0.946
0.7	10	50	0.694	0.945	0.943	0.947	0.951	0.943	0.941	0.945	0.947
0.8	10	50	0.795	0.947	0.945	0.949	0.950	0.944	0.943	0.946	0.946
0.9	10	50	0.897	0.950	0.948	0.952	0.950	0.947	0.945	0.948	0.948
0.1	2	60	0.099	0.938	0.937	0.952	0.951	0.949	0.948	0.961	0.949
0.2	2	60	0.198	0.938	0.938	0.952	0.950	0.948	0.947	0.961	0.948
0.3	2	60	0.296	0.938	0.937	0.952	0.950	0.948	0.947	0.962	0.948
0.4	2	60	0.395	0.939	0.938	0.952	0.950	0.948	0.947	0.961	0.948
0.5	2	60	0.495	0.939	0.938	0.951	0.950	0.948	0.947	0.961	0.948
0.6	2	60	0.594	0.940	0.938	0.951	0.951	0.949	0.948	0.962	0.949
0.7	2	60	0.695	0.939	0.938	0.951	0.951	0.949	0.948	0.961	0.949
0.8	2	60	0.796	0.939	0.938	0.951	0.952	0.950	0.949	0.962	0.950

0.9	2	60	0.897	0.939	0.938	0.951	0.952	0.950	0.949	0.962	0.950
0.1	3	60	0.099	0.939	0.938	0.946	0.948	0.943	0.942	0.949	0.946
0.2	3	60	0.198	0.939	0.938	0.946	0.949	0.943	0.942	0.949	0.947
0.3	3	60	0.297	0.941	0.939	0.947	0.950	0.944	0.943	0.950	0.947
0.4	3	60	0.396	0.940	0.938	0.948	0.950	0.945	0.944	0.951	0.947
0.5	3	60	0.495	0.942	0.941	0.948	0.950	0.945	0.944	0.952	0.947
0.6	3	60	0.595	0.943	0.942	0.949	0.951	0.947	0.946	0.953	0.948
0.7	3	60	0.695	0.944	0.943	0.950	0.951	0.948	0.947	0.953	0.949
0.8	3	60	0.796	0.945	0.944	0.951	0.950	0.948	0.947	0.954	0.949
0.9	3	60	0.897	0.945	0.944	0.951	0.950	0.948	0.947	0.954	0.948
0.1	4	60	0.099	0.938	0.937	0.942	0.949	0.940	0.939	0.944	0.947
0.2	4	60	0.198	0.940	0.939	0.944	0.949	0.942	0.941	0.945	0.948
0.3	4	60	0.297	0.940	0.939	0.944	0.949	0.942	0.941	0.946	0.947
0.4	4	60	0.396	0.941	0.939	0.944	0.949	0.943	0.941	0.947	0.946
0.5	4	60	0.495	0.942	0.940	0.946	0.949	0.944	0.942	0.948	0.947
0.6	4	60	0.595	0.943	0.941	0.947	0.950	0.945	0.943	0.949	0.947
0.7	4	60	0.695	0.944	0.943	0.948	0.949	0.946	0.945	0.950	0.948
0.8	4	60	0.795	0.946	0.944	0.950	0.950	0.946	0.945	0.950	0.947
0.9	4	60	0.897	0.948	0.946	0.951	0.949	0.947	0.945	0.951	0.947
0.1	5	60	0.099	0.938	0.936	0.940	0.947	0.939	0.937	0.942	0.945
0.2	5	60	0.198	0.938	0.936	0.941	0.947	0.940	0.938	0.942	0.944
0.3	5	60	0.297	0.939	0.937	0.942	0.947	0.940	0.939	0.943	0.945
0.4	5	60	0.396	0.940	0.938	0.943	0.946	0.942	0.941	0.945	0.946
0.5	5	60	0.495	0.941	0.939	0.943	0.948	0.942	0.941	0.946	0.946
0.6	5	60	0.595	0.942	0.941	0.945	0.948	0.944	0.942	0.947	0.946
0.7	5	60	0.695	0.944	0.942	0.947	0.948	0.944	0.943	0.947	0.946
0.8	5	60	0.795	0.944	0.943	0.948	0.948	0.945	0.943	0.948	0.946
0.9	5	60	0.897	0.946	0.945	0.948	0.948	0.945	0.944	0.948	0.946
0.1	6	60	0.099	0.937	0.935	0.940	0.948	0.938	0.936	0.941	0.947
0.2	6	60	0.198	0.938	0.936	0.940	0.949	0.939	0.938	0.941	0.946
0.3	6	60	0.297	0.940	0.938	0.942	0.949	0.941	0.939	0.944	0.946
0.4	6	60	0.396	0.941	0.939	0.943	0.948	0.942	0.940	0.944	0.946
0.5	6	60	0.495	0.940	0.938	0.943	0.948	0.943	0.942	0.946	0.946
0.6	6	60	0.595	0.941	0.940	0.944	0.948	0.944	0.942	0.946	0.947
0.7	6	60	0.695	0.943	0.942	0.946	0.949	0.944	0.942	0.946	0.946
0.8	6	60	0.796	0.946	0.944	0.948	0.949	0.945	0.944	0.948	0.947
0.9	6	60	0.897	0.947	0.945	0.949	0.949	0.947	0.946	0.949	0.948
0.1	7	60	0.100	0.937	0.935	0.939	0.949	0.938	0.936	0.940	0.946
0.2	7	60	0.199	0.937	0.936	0.939	0.948	0.938	0.937	0.940	0.945
0.3	7	60	0.298	0.937	0.936	0.940	0.947	0.938	0.937	0.940	0.945
0.4	7	60	0.397	0.938	0.936	0.940	0.946	0.939	0.938	0.941	0.945
0.5	7	60	0.496	0.940	0.938	0.942	0.947	0.942	0.939	0.943	0.945
0.6	7	60	0.595	0.941	0.940	0.944	0.948	0.943	0.941	0.945	0.945
0.7	7	60	0.695	0.945	0.943	0.946	0.949	0.944	0.943	0.946	0.945
0.8	7	60	0.796	0.945	0.944	0.947	0.948	0.945	0.943	0.947	0.945
0.9	7	60	0.897	0.947	0.945	0.949	0.949	0.946	0.944	0.948	0.946
0.1	8	60	0.100	0.936	0.935	0.938	0.947	0.937	0.935	0.939	0.945
0.2	8	60	0.199	0.937	0.935	0.938	0.948	0.938	0.936	0.939	0.945
0.3	8	60	0.298	0.939	0.937	0.941	0.948	0.939	0.937	0.940	0.945
0.4	8	60	0.397	0.940	0.938	0.942	0.947	0.940	0.939	0.942	0.946

0.5	8	60	0.496	0.940	0.939	0.942	0.947	0.942	0.940	0.943	0.946
0.6	8	60	0.595	0.941	0.940	0.943	0.947	0.942	0.941	0.944	0.946
0.7	8	60	0.695	0.942	0.941	0.944	0.947	0.944	0.942	0.945	0.946
0.8	8	60	0.796	0.945	0.943	0.946	0.948	0.945	0.943	0.946	0.946
0.9	8	60	0.897	0.947	0.946	0.949	0.948	0.945	0.943	0.947	0.946
0.1	9	60	0.100	0.936	0.935	0.938	0.948	0.937	0.935	0.938	0.946
0.2	9	60	0.199	0.936	0.935	0.938	0.948	0.937	0.935	0.939	0.945
0.3	9	60	0.298	0.937	0.935	0.939	0.947	0.937	0.936	0.939	0.945
0.4	9	60	0.396	0.938	0.936	0.940	0.946	0.939	0.938	0.941	0.945
0.5	9	60	0.496	0.940	0.938	0.941	0.947	0.940	0.938	0.942	0.945
0.6	9	60	0.595	0.941	0.940	0.943	0.947	0.941	0.939	0.943	0.945
0.7	9	60	0.695	0.943	0.942	0.945	0.948	0.942	0.940	0.944	0.945
0.8	9	60	0.796	0.945	0.943	0.946	0.948	0.944	0.942	0.946	0.946
0.9	9	60	0.897	0.946	0.945	0.948	0.947	0.945	0.944	0.946	0.946
0.1	10	60	0.100	0.937	0.935	0.939	0.949	0.938	0.936	0.939	0.947
0.2	10	60	0.199	0.938	0.937	0.939	0.949	0.939	0.937	0.940	0.946
0.3	10	60	0.298	0.939	0.937	0.940	0.949	0.940	0.938	0.941	0.945
0.4	10	60	0.397	0.940	0.938	0.941	0.949	0.941	0.939	0.942	0.946
0.5	10	60	0.496	0.941	0.939	0.943	0.948	0.942	0.940	0.943	0.946
0.6	10	60	0.595	0.943	0.941	0.944	0.948	0.943	0.941	0.944	0.946
0.7	10	60	0.695	0.944	0.943	0.946	0.948	0.943	0.942	0.945	0.946
0.8	10	60	0.796	0.945	0.943	0.947	0.948	0.944	0.942	0.946	0.946
0.9	10	60	0.897	0.946	0.944	0.948	0.948	0.945	0.943	0.946	0.946
0.1	2	70	0.099	0.939	0.939	0.951	0.950	0.948	0.947	0.959	0.948
0.2	2	70	0.197	0.940	0.940	0.952	0.951	0.948	0.947	0.960	0.948
0.3	2	70	0.296	0.941	0.940	0.952	0.950	0.948	0.948	0.960	0.948
0.4	2	70	0.395	0.941	0.940	0.952	0.951	0.949	0.948	0.959	0.949
0.5	2	70	0.495	0.941	0.940	0.952	0.950	0.949	0.948	0.960	0.949
0.6	2	70	0.595	0.941	0.941	0.952	0.951	0.949	0.948	0.960	0.949
0.7	2	70	0.695	0.941	0.940	0.952	0.951	0.949	0.948	0.961	0.949
0.8	2	70	0.796	0.942	0.941	0.951	0.952	0.950	0.949	0.960	0.950
0.9	2	70	0.898	0.942	0.942	0.951	0.951	0.950	0.949	0.961	0.950
0.1	3	70	0.099	0.943	0.942	0.949	0.951	0.946	0.945	0.951	0.949
0.2	3	70	0.198	0.944	0.943	0.950	0.952	0.947	0.946	0.952	0.951
0.3	3	70	0.297	0.943	0.942	0.949	0.951	0.947	0.946	0.953	0.949
0.4	3	70	0.396	0.944	0.943	0.948	0.950	0.946	0.945	0.952	0.948
0.5	3	70	0.496	0.944	0.943	0.949	0.950	0.947	0.946	0.952	0.948
0.6	3	70	0.595	0.944	0.943	0.949	0.950	0.947	0.946	0.952	0.948
0.7	3	70	0.695	0.945	0.944	0.950	0.950	0.948	0.946	0.953	0.948
0.8	3	70	0.796	0.946	0.945	0.951	0.951	0.948	0.947	0.953	0.948
0.9	3	70	0.898	0.946	0.945	0.951	0.950	0.948	0.947	0.954	0.948
0.1	4	70	0.099	0.940	0.938	0.943	0.950	0.941	0.940	0.945	0.948
0.2	4	70	0.198	0.942	0.941	0.946	0.949	0.943	0.942	0.947	0.949
0.3	4	70	0.297	0.943	0.941	0.947	0.950	0.945	0.944	0.948	0.949
0.4	4	70	0.396	0.943	0.941	0.947	0.950	0.945	0.944	0.948	0.948
0.5	4	70	0.496	0.944	0.942	0.948	0.950	0.945	0.944	0.949	0.947
0.6	4	70	0.595	0.944	0.943	0.948	0.950	0.946	0.944	0.949	0.948
0.7	4	70	0.695	0.945	0.944	0.949	0.950	0.946	0.945	0.949	0.948
0.8	4	70	0.796	0.946	0.945	0.950	0.950	0.947	0.946	0.950	0.948
0.9	4	70	0.898	0.948	0.947	0.951	0.948	0.946	0.945	0.950	0.947

0.1	5	70	0.099	0.937	0.936	0.940	0.948	0.939	0.937	0.941	0.946
0.2	5	70	0.198	0.938	0.937	0.941	0.949	0.940	0.939	0.942	0.945
0.3	5	70	0.297	0.941	0.939	0.943	0.948	0.942	0.941	0.945	0.946
0.4	5	70	0.396	0.942	0.941	0.945	0.949	0.943	0.942	0.946	0.947
0.5	5	70	0.496	0.942	0.941	0.945	0.949	0.944	0.942	0.946	0.947
0.6	5	70	0.595	0.944	0.943	0.947	0.949	0.944	0.943	0.946	0.947
0.7	5	70	0.695	0.947	0.945	0.950	0.950	0.945	0.944	0.948	0.947
0.8	5	70	0.796	0.948	0.947	0.951	0.950	0.946	0.945	0.948	0.947
0.9	5	70	0.898	0.950	0.948	0.952	0.949	0.946	0.945	0.949	0.947
0.1	6	70	0.099	0.940	0.938	0.941	0.947	0.940	0.939	0.942	0.946
0.2	6	70	0.199	0.941	0.940	0.943	0.948	0.941	0.940	0.944	0.947
0.3	6	70	0.298	0.942	0.940	0.944	0.949	0.943	0.941	0.944	0.947
0.4	6	70	0.397	0.942	0.941	0.944	0.949	0.944	0.943	0.946	0.947
0.5	6	70	0.496	0.943	0.941	0.945	0.949	0.944	0.943	0.946	0.947
0.6	6	70	0.596	0.945	0.943	0.947	0.949	0.945	0.943	0.947	0.947
0.7	6	70	0.696	0.945	0.944	0.947	0.949	0.945	0.944	0.948	0.948
0.8	6	70	0.796	0.947	0.945	0.949	0.950	0.947	0.945	0.949	0.948
0.9	6	70	0.898	0.949	0.947	0.951	0.949	0.946	0.944	0.948	0.947
0.1	7	70	0.099	0.937	0.936	0.938	0.949	0.937	0.936	0.938	0.947
0.2	7	70	0.199	0.939	0.938	0.940	0.949	0.940	0.939	0.941	0.946
0.3	7	70	0.298	0.940	0.939	0.942	0.948	0.941	0.940	0.943	0.947
0.4	7	70	0.397	0.941	0.940	0.943	0.948	0.942	0.940	0.943	0.947
0.5	7	70	0.496	0.943	0.941	0.945	0.949	0.942	0.940	0.944	0.946
0.6	7	70	0.596	0.943	0.941	0.945	0.948	0.944	0.943	0.946	0.946
0.7	7	70	0.696	0.945	0.943	0.947	0.949	0.944	0.943	0.946	0.947
0.8	7	70	0.796	0.947	0.945	0.949	0.949	0.946	0.945	0.947	0.947
0.9	7	70	0.898	0.949	0.947	0.950	0.949	0.946	0.944	0.947	0.946
0.1	8	70	0.099	0.938	0.937	0.939	0.949	0.938	0.936	0.940	0.947
0.2	8	70	0.199	0.939	0.938	0.940	0.948	0.939	0.937	0.941	0.946
0.3	8	70	0.298	0.940	0.939	0.941	0.949	0.941	0.940	0.942	0.946
0.4	8	70	0.397	0.942	0.940	0.943	0.949	0.942	0.941	0.944	0.946
0.5	8	70	0.496	0.942	0.941	0.944	0.949	0.943	0.942	0.944	0.946
0.6	8	70	0.596	0.944	0.942	0.946	0.949	0.944	0.943	0.945	0.947
0.7	8	70	0.696	0.945	0.944	0.947	0.949	0.944	0.943	0.946	0.947
0.8	8	70	0.796	0.947	0.946	0.948	0.949	0.945	0.944	0.947	0.947
0.9	8	70	0.898	0.947	0.946	0.948	0.949	0.947	0.945	0.948	0.948
0.1	9	70	0.100	0.938	0.937	0.939	0.951	0.938	0.937	0.939	0.948
0.2	9	70	0.199	0.940	0.938	0.941	0.950	0.940	0.939	0.942	0.947
0.3	9	70	0.298	0.941	0.939	0.943	0.949	0.942	0.940	0.943	0.947
0.4	9	70	0.397	0.942	0.940	0.943	0.950	0.942	0.941	0.943	0.947
0.5	9	70	0.496	0.944	0.942	0.945	0.950	0.943	0.941	0.943	0.947
0.6	9	70	0.596	0.945	0.944	0.946	0.950	0.944	0.942	0.945	0.946
0.7	9	70	0.696	0.946	0.944	0.947	0.949	0.945	0.943	0.946	0.947
0.8	9	70	0.796	0.946	0.945	0.948	0.949	0.946	0.944	0.947	0.947
0.9	9	70	0.898	0.947	0.945	0.948	0.948	0.947	0.945	0.948	0.947
0.1	10	70	0.100	0.940	0.939	0.941	0.952	0.941	0.939	0.942	0.950
0.2	10	70	0.199	0.941	0.939	0.941	0.950	0.940	0.939	0.942	0.948
0.3	10	70	0.298	0.941	0.939	0.942	0.950	0.942	0.941	0.943	0.947
0.4	10	70	0.397	0.943	0.942	0.944	0.950	0.943	0.941	0.944	0.948
0.5	10	70	0.496	0.944	0.943	0.946	0.949	0.943	0.942	0.944	0.948

0.6	10	70	0.596	0.944	0.942	0.945	0.948	0.944	0.943	0.945	0.946
0.7	10	70	0.696	0.944	0.943	0.945	0.947	0.945	0.943	0.946	0.946
0.8	10	70	0.796	0.945	0.944	0.946	0.947	0.946	0.944	0.947	0.946
0.9	10	70	0.898	0.947	0.946	0.948	0.947	0.946	0.944	0.947	0.946
0.1	2	80	0.098	0.941	0.940	0.951	0.950	0.949	0.948	0.957	0.949
0.2	2	80	0.197	0.941	0.940	0.951	0.950	0.949	0.948	0.958	0.949
0.3	2	80	0.296	0.942	0.941	0.952	0.951	0.949	0.948	0.959	0.949
0.4	2	80	0.396	0.943	0.942	0.954	0.952	0.950	0.949	0.960	0.950
0.5	2	80	0.495	0.944	0.943	0.954	0.953	0.951	0.950	0.961	0.951
0.6	2	80	0.595	0.944	0.943	0.953	0.953	0.952	0.951	0.961	0.952
0.7	2	80	0.695	0.943	0.942	0.953	0.953	0.951	0.950	0.961	0.951
0.8	2	80	0.796	0.944	0.943	0.952	0.952	0.950	0.950	0.961	0.950
0.9	2	80	0.898	0.943	0.942	0.952	0.951	0.949	0.949	0.960	0.949
0.1	3	80	0.099	0.945	0.944	0.950	0.952	0.947	0.946	0.952	0.951
0.2	3	80	0.198	0.945	0.945	0.950	0.952	0.948	0.947	0.952	0.950
0.3	3	80	0.297	0.946	0.944	0.950	0.951	0.948	0.947	0.953	0.951
0.4	3	80	0.397	0.945	0.944	0.950	0.951	0.948	0.947	0.953	0.950
0.5	3	80	0.496	0.945	0.945	0.950	0.951	0.948	0.948	0.953	0.950
0.6	3	80	0.596	0.947	0.946	0.951	0.951	0.948	0.947	0.953	0.949
0.7	3	80	0.696	0.947	0.946	0.952	0.951	0.948	0.947	0.953	0.949
0.8	3	80	0.797	0.947	0.946	0.951	0.951	0.948	0.948	0.954	0.949
0.9	3	80	0.898	0.946	0.946	0.951	0.951	0.949	0.948	0.954	0.949
0.1	4	80	0.099	0.944	0.943	0.947	0.952	0.946	0.944	0.949	0.950
0.2	4	80	0.198	0.943	0.942	0.946	0.951	0.945	0.943	0.948	0.949
0.3	4	80	0.297	0.943	0.942	0.946	0.949	0.945	0.944	0.948	0.948
0.4	4	80	0.397	0.944	0.943	0.947	0.951	0.946	0.944	0.949	0.949
0.5	4	80	0.496	0.944	0.943	0.947	0.950	0.946	0.945	0.949	0.948
0.6	4	80	0.596	0.945	0.944	0.948	0.949	0.946	0.944	0.949	0.948
0.7	4	80	0.696	0.946	0.945	0.950	0.949	0.945	0.944	0.949	0.947
0.8	4	80	0.797	0.946	0.945	0.949	0.949	0.946	0.945	0.949	0.947
0.9	4	80	0.898	0.947	0.946	0.950	0.949	0.948	0.946	0.950	0.948
0.1	5	80	0.100	0.944	0.943	0.947	0.951	0.945	0.944	0.947	0.950
0.2	5	80	0.199	0.944	0.943	0.946	0.951	0.946	0.945	0.948	0.949
0.3	5	80	0.298	0.945	0.944	0.947	0.952	0.946	0.945	0.949	0.950
0.4	5	80	0.397	0.945	0.944	0.948	0.951	0.946	0.945	0.949	0.950
0.5	5	80	0.496	0.945	0.944	0.948	0.951	0.946	0.945	0.949	0.949
0.6	5	80	0.596	0.946	0.944	0.948	0.950	0.946	0.945	0.948	0.948
0.7	5	80	0.696	0.946	0.945	0.949	0.949	0.946	0.945	0.949	0.948
0.8	5	80	0.797	0.947	0.946	0.950	0.950	0.947	0.946	0.949	0.948
0.9	5	80	0.898	0.948	0.947	0.950	0.950	0.947	0.946	0.949	0.947
0.1	6	80	0.099	0.944	0.943	0.946	0.952	0.944	0.943	0.946	0.950
0.2	6	80	0.199	0.945	0.943	0.946	0.952	0.946	0.945	0.947	0.951
0.3	6	80	0.298	0.945	0.944	0.947	0.952	0.946	0.944	0.948	0.951
0.4	6	80	0.397	0.946	0.944	0.947	0.951	0.947	0.945	0.948	0.950
0.5	6	80	0.496	0.945	0.943	0.946	0.950	0.947	0.946	0.949	0.949
0.6	6	80	0.596	0.945	0.944	0.948	0.950	0.946	0.945	0.948	0.949
0.7	6	80	0.696	0.947	0.945	0.948	0.950	0.946	0.945	0.948	0.948
0.8	6	80	0.797	0.948	0.946	0.950	0.951	0.946	0.945	0.948	0.947
0.9	6	80	0.898	0.949	0.947	0.950	0.950	0.947	0.946	0.950	0.948
0.1	7	80	0.099	0.943	0.941	0.944	0.951	0.943	0.942	0.945	0.949

0.2	7	80	0.199	0.944	0.942	0.946	0.951	0.944	0.943	0.946	0.950
0.3	7	80	0.298	0.943	0.942	0.945	0.950	0.944	0.943	0.945	0.949
0.4	7	80	0.397	0.943	0.942	0.945	0.950	0.944	0.943	0.945	0.948
0.5	7	80	0.496	0.944	0.943	0.946	0.950	0.945	0.943	0.946	0.948
0.6	7	80	0.596	0.946	0.944	0.947	0.950	0.945	0.944	0.947	0.948
0.7	7	80	0.696	0.946	0.945	0.948	0.950	0.947	0.945	0.948	0.948
0.8	7	80	0.797	0.946	0.945	0.948	0.949	0.947	0.946	0.949	0.948
0.9	7	80	0.898	0.947	0.946	0.949	0.949	0.947	0.946	0.949	0.948
0.1	8	80	0.099	0.941	0.940	0.942	0.951	0.942	0.940	0.942	0.948
0.2	8	80	0.199	0.942	0.940	0.943	0.950	0.942	0.940	0.943	0.948
0.3	8	80	0.298	0.943	0.941	0.944	0.950	0.943	0.942	0.944	0.947
0.4	8	80	0.397	0.943	0.942	0.945	0.949	0.944	0.943	0.945	0.948
0.5	8	80	0.496	0.944	0.942	0.946	0.948	0.945	0.944	0.946	0.948
0.6	8	80	0.596	0.944	0.943	0.946	0.948	0.946	0.945	0.947	0.948
0.7	8	80	0.696	0.945	0.944	0.947	0.949	0.947	0.945	0.948	0.948
0.8	8	80	0.797	0.946	0.945	0.947	0.949	0.946	0.945	0.948	0.948
0.9	8	80	0.898	0.947	0.946	0.948	0.948	0.946	0.945	0.948	0.948
0.1	9	80	0.099	0.941	0.939	0.942	0.949	0.941	0.940	0.942	0.947
0.2	9	80	0.199	0.942	0.941	0.943	0.948	0.943	0.941	0.943	0.947
0.3	9	80	0.298	0.941	0.940	0.943	0.950	0.943	0.941	0.944	0.947
0.4	9	80	0.397	0.943	0.941	0.944	0.949	0.942	0.941	0.944	0.948
0.5	9	80	0.496	0.944	0.943	0.945	0.949	0.943	0.942	0.944	0.948
0.6	9	80	0.596	0.945	0.943	0.946	0.949	0.945	0.943	0.946	0.947
0.7	9	80	0.696	0.945	0.944	0.947	0.949	0.945	0.944	0.946	0.947
0.8	9	80	0.797	0.947	0.945	0.948	0.949	0.945	0.944	0.947	0.947
0.9	9	80	0.898	0.948	0.947	0.949	0.949	0.946	0.945	0.948	0.947
0.1	10	80	0.100	0.942	0.940	0.943	0.950	0.942	0.941	0.943	0.949
0.2	10	80	0.199	0.941	0.940	0.942	0.950	0.943	0.941	0.943	0.947
0.3	10	80	0.298	0.942	0.941	0.943	0.950	0.943	0.942	0.944	0.947
0.4	10	80	0.397	0.943	0.942	0.944	0.950	0.943	0.941	0.944	0.947
0.5	10	80	0.497	0.945	0.943	0.945	0.950	0.944	0.942	0.944	0.948
0.6	10	80	0.596	0.945	0.944	0.945	0.949	0.945	0.944	0.945	0.947
0.7	10	80	0.696	0.945	0.944	0.947	0.949	0.945	0.944	0.946	0.947
0.8	10	80	0.797	0.947	0.945	0.947	0.949	0.946	0.945	0.947	0.948
0.9	10	80	0.898	0.948	0.947	0.949	0.949	0.946	0.945	0.947	0.947
0.1	2	90	0.099	0.940	0.939	0.950	0.949	0.947	0.946	0.956	0.947
0.2	2	90	0.198	0.941	0.940	0.950	0.948	0.947	0.946	0.956	0.947
0.3	2	90	0.297	0.941	0.940	0.950	0.949	0.948	0.947	0.956	0.948
0.4	2	90	0.397	0.941	0.941	0.950	0.949	0.948	0.947	0.956	0.948
0.5	2	90	0.496	0.941	0.941	0.950	0.949	0.948	0.947	0.956	0.948
0.6	2	90	0.596	0.942	0.941	0.951	0.949	0.948	0.947	0.955	0.948
0.7	2	90	0.696	0.944	0.943	0.951	0.949	0.948	0.947	0.956	0.948
0.8	2	90	0.797	0.944	0.943	0.951	0.949	0.948	0.947	0.957	0.948
0.9	2	90	0.898	0.943	0.943	0.951	0.951	0.949	0.949	0.957	0.949
0.1	3	90	0.100	0.941	0.940	0.946	0.948	0.944	0.943	0.948	0.947
0.2	3	90	0.199	0.941	0.940	0.945	0.947	0.944	0.943	0.948	0.946
0.3	3	90	0.298	0.942	0.941	0.946	0.948	0.944	0.943	0.949	0.947
0.4	3	90	0.397	0.944	0.943	0.948	0.949	0.946	0.945	0.950	0.948
0.5	3	90	0.497	0.943	0.942	0.947	0.949	0.946	0.945	0.951	0.948
0.6	3	90	0.596	0.943	0.943	0.946	0.948	0.945	0.944	0.950	0.946

0.7	3	90	0.697	0.944	0.943	0.948	0.948	0.945	0.944	0.950	0.946
0.8	3	90	0.797	0.945	0.945	0.949	0.949	0.946	0.945	0.950	0.947
0.9	3	90	0.898	0.945	0.944	0.949	0.949	0.947	0.946	0.952	0.948
0.1	4	90	0.100	0.942	0.941	0.944	0.948	0.943	0.942	0.946	0.947
0.2	4	90	0.199	0.942	0.941	0.945	0.949	0.944	0.943	0.946	0.947
0.3	4	90	0.298	0.944	0.943	0.947	0.950	0.945	0.945	0.948	0.948
0.4	4	90	0.397	0.945	0.944	0.948	0.950	0.947	0.946	0.949	0.949
0.5	4	90	0.497	0.946	0.945	0.948	0.950	0.948	0.946	0.950	0.949
0.6	4	90	0.597	0.946	0.945	0.949	0.950	0.948	0.948	0.951	0.950
0.7	4	90	0.697	0.947	0.946	0.948	0.950	0.948	0.946	0.951	0.949
0.8	4	90	0.797	0.947	0.946	0.949	0.950	0.948	0.947	0.950	0.948
0.9	4	90	0.898	0.946	0.945	0.949	0.949	0.948	0.947	0.951	0.948
0.1	5	90	0.100	0.941	0.940	0.943	0.950	0.942	0.941	0.944	0.947
0.2	5	90	0.199	0.943	0.942	0.946	0.950	0.944	0.943	0.946	0.949
0.3	5	90	0.298	0.944	0.943	0.946	0.950	0.945	0.944	0.947	0.948
0.4	5	90	0.398	0.944	0.943	0.946	0.950	0.946	0.944	0.948	0.948
0.5	5	90	0.497	0.944	0.944	0.947	0.949	0.946	0.945	0.948	0.948
0.6	5	90	0.597	0.945	0.945	0.947	0.949	0.946	0.945	0.948	0.948
0.7	5	90	0.697	0.946	0.945	0.948	0.949	0.947	0.946	0.949	0.948
0.8	5	90	0.797	0.946	0.945	0.948	0.949	0.948	0.947	0.950	0.948
0.9	5	90	0.898	0.946	0.945	0.948	0.949	0.947	0.946	0.950	0.948
0.1	6	90	0.100	0.943	0.942	0.944	0.951	0.944	0.943	0.945	0.949
0.2	6	90	0.199	0.944	0.943	0.946	0.950	0.945	0.944	0.946	0.949
0.3	6	90	0.298	0.945	0.943	0.946	0.950	0.945	0.944	0.947	0.949
0.4	6	90	0.398	0.945	0.944	0.947	0.950	0.946	0.945	0.947	0.949
0.5	6	90	0.497	0.946	0.944	0.947	0.950	0.946	0.945	0.948	0.949
0.6	6	90	0.597	0.946	0.945	0.947	0.949	0.946	0.945	0.948	0.948
0.7	6	90	0.697	0.945	0.944	0.947	0.948	0.946	0.945	0.948	0.948
0.8	6	90	0.797	0.945	0.945	0.947	0.948	0.947	0.946	0.948	0.948
0.9	6	90	0.898	0.946	0.945	0.948	0.948	0.947	0.945	0.948	0.947
0.1	7	90	0.100	0.941	0.940	0.943	0.950	0.942	0.941	0.943	0.948
0.2	7	90	0.199	0.943	0.942	0.944	0.949	0.943	0.942	0.944	0.947
0.3	7	90	0.298	0.943	0.942	0.944	0.949	0.943	0.942	0.944	0.947
0.4	7	90	0.398	0.944	0.943	0.946	0.949	0.944	0.943	0.946	0.948
0.5	7	90	0.497	0.944	0.944	0.946	0.950	0.946	0.945	0.947	0.948
0.6	7	90	0.597	0.945	0.944	0.946	0.948	0.946	0.945	0.948	0.948
0.7	7	90	0.697	0.945	0.944	0.946	0.948	0.947	0.946	0.948	0.947
0.8	7	90	0.797	0.945	0.944	0.946	0.948	0.947	0.945	0.948	0.948
0.9	7	90	0.898	0.946	0.945	0.948	0.948	0.947	0.946	0.948	0.947
0.1	8	90	0.100	0.940	0.939	0.941	0.949	0.940	0.939	0.941	0.946
0.2	8	90	0.199	0.943	0.941	0.944	0.949	0.943	0.941	0.944	0.948
0.3	8	90	0.298	0.943	0.941	0.944	0.949	0.943	0.942	0.944	0.947
0.4	8	90	0.398	0.943	0.942	0.944	0.949	0.944	0.943	0.946	0.947
0.5	8	90	0.497	0.945	0.943	0.946	0.949	0.945	0.944	0.946	0.948
0.6	8	90	0.597	0.945	0.944	0.946	0.949	0.946	0.944	0.947	0.949
0.7	8	90	0.697	0.945	0.945	0.946	0.949	0.946	0.945	0.948	0.947
0.8	8	90	0.797	0.946	0.944	0.947	0.949	0.947	0.946	0.948	0.948
0.9	8	90	0.898	0.946	0.945	0.947	0.948	0.947	0.946	0.949	0.947
0.1	9	90	0.100	0.942	0.941	0.943	0.949	0.942	0.941	0.943	0.948
0.2	9	90	0.199	0.943	0.942	0.944	0.950	0.943	0.942	0.944	0.948

0.3	9	90	0.298	0.943	0.941	0.944	0.949	0.943	0.942	0.944	0.947
0.4	9	90	0.398	0.944	0.942	0.945	0.948	0.945	0.943	0.945	0.948
0.5	9	90	0.497	0.945	0.943	0.946	0.949	0.946	0.944	0.947	0.948
0.6	9	90	0.597	0.945	0.944	0.946	0.949	0.946	0.945	0.947	0.948
0.7	9	90	0.697	0.947	0.945	0.947	0.949	0.946	0.945	0.947	0.948
0.8	9	90	0.797	0.946	0.945	0.947	0.949	0.947	0.946	0.948	0.948
0.9	9	90	0.898	0.947	0.946	0.948	0.948	0.948	0.946	0.949	0.948
0.1	10	90	0.100	0.943	0.942	0.944	0.950	0.943	0.942	0.944	0.949
0.2	10	90	0.199	0.944	0.942	0.945	0.950	0.944	0.942	0.945	0.949
0.3	10	90	0.298	0.944	0.943	0.945	0.949	0.944	0.943	0.945	0.948
0.4	10	90	0.398	0.944	0.943	0.944	0.949	0.944	0.943	0.945	0.947
0.5	10	90	0.497	0.944	0.943	0.945	0.949	0.945	0.944	0.945	0.947
0.6	10	90	0.597	0.945	0.944	0.946	0.950	0.945	0.944	0.946	0.948
0.7	10	90	0.697	0.946	0.945	0.947	0.949	0.947	0.946	0.948	0.948
0.8	10	90	0.797	0.946	0.945	0.946	0.948	0.948	0.947	0.949	0.949
0.9	10	90	0.898	0.946	0.945	0.947	0.948	0.948	0.947	0.949	0.949
0.1	2	100	0.098	0.943	0.942	0.951	0.949	0.948	0.948	0.956	0.948
0.2	2	100	0.198	0.943	0.942	0.951	0.950	0.949	0.948	0.957	0.949
0.3	2	100	0.297	0.942	0.941	0.951	0.950	0.949	0.948	0.956	0.949
0.4	2	100	0.396	0.943	0.942	0.951	0.949	0.948	0.947	0.956	0.948
0.5	2	100	0.496	0.943	0.942	0.951	0.948	0.947	0.947	0.955	0.947
0.6	2	100	0.596	0.943	0.942	0.950	0.948	0.946	0.946	0.955	0.946
0.7	2	100	0.696	0.942	0.942	0.950	0.949	0.948	0.947	0.956	0.948
0.8	2	100	0.797	0.942	0.941	0.950	0.950	0.949	0.948	0.956	0.949
0.9	2	100	0.898	0.941	0.940	0.950	0.950	0.949	0.948	0.956	0.949
0.1	3	100	0.100	0.940	0.939	0.945	0.947	0.943	0.942	0.948	0.946
0.2	3	100	0.199	0.941	0.940	0.945	0.948	0.943	0.943	0.948	0.946
0.3	3	100	0.298	0.943	0.942	0.946	0.948	0.945	0.944	0.948	0.947
0.4	3	100	0.398	0.942	0.941	0.946	0.947	0.944	0.943	0.948	0.946
0.5	3	100	0.497	0.942	0.941	0.946	0.947	0.943	0.942	0.948	0.944
0.6	3	100	0.597	0.943	0.942	0.947	0.947	0.945	0.944	0.948	0.945
0.7	3	100	0.697	0.942	0.942	0.947	0.948	0.945	0.945	0.949	0.946
0.8	3	100	0.797	0.944	0.943	0.947	0.947	0.945	0.944	0.949	0.946
0.9	3	100	0.898	0.944	0.943	0.948	0.947	0.946	0.945	0.950	0.946
0.1	4	100	0.100	0.941	0.940	0.943	0.948	0.942	0.942	0.945	0.946
0.2	4	100	0.199	0.942	0.940	0.944	0.947	0.943	0.942	0.945	0.946
0.3	4	100	0.298	0.942	0.941	0.944	0.948	0.943	0.942	0.946	0.946
0.4	4	100	0.398	0.941	0.941	0.944	0.946	0.944	0.943	0.946	0.945
0.5	4	100	0.497	0.942	0.941	0.945	0.946	0.944	0.943	0.946	0.946
0.6	4	100	0.597	0.942	0.942	0.945	0.946	0.944	0.943	0.946	0.946
0.7	4	100	0.697	0.944	0.943	0.946	0.946	0.944	0.943	0.947	0.945
0.8	4	100	0.797	0.945	0.944	0.947	0.946	0.944	0.944	0.947	0.945
0.9	4	100	0.898	0.944	0.944	0.946	0.947	0.946	0.945	0.948	0.946
0.1	5	100	0.100	0.942	0.941	0.944	0.949	0.943	0.942	0.945	0.948
0.2	5	100	0.199	0.941	0.941	0.943	0.947	0.942	0.941	0.944	0.947
0.3	5	100	0.298	0.942	0.941	0.944	0.947	0.943	0.942	0.945	0.945
0.4	5	100	0.398	0.942	0.941	0.944	0.947	0.943	0.942	0.945	0.945
0.5	5	100	0.497	0.944	0.942	0.945	0.947	0.944	0.943	0.945	0.947
0.6	5	100	0.597	0.945	0.944	0.946	0.948	0.945	0.944	0.947	0.947
0.7	5	100	0.697	0.945	0.944	0.947	0.948	0.945	0.944	0.947	0.947

0.8	5	100	0.797	0.945	0.944	0.947	0.948	0.946	0.945	0.948	0.947
0.9	5	100	0.898	0.946	0.945	0.948	0.947	0.947	0.946	0.949	0.947
0.1	6	100	0.100	0.943	0.942	0.944	0.949	0.943	0.942	0.945	0.949
0.2	6	100	0.199	0.943	0.942	0.944	0.949	0.943	0.943	0.945	0.948
0.3	6	100	0.298	0.943	0.943	0.944	0.949	0.944	0.943	0.945	0.947
0.4	6	100	0.398	0.945	0.944	0.946	0.949	0.944	0.943	0.945	0.948
0.5	6	100	0.497	0.945	0.944	0.946	0.949	0.945	0.944	0.947	0.948
0.6	6	100	0.597	0.946	0.944	0.947	0.949	0.945	0.944	0.947	0.947
0.7	6	100	0.697	0.946	0.945	0.947	0.948	0.946	0.945	0.947	0.947
0.8	6	100	0.797	0.947	0.945	0.948	0.948	0.946	0.945	0.948	0.947
0.9	6	100	0.898	0.947	0.946	0.949	0.948	0.946	0.945	0.948	0.947
0.1	7	100	0.100	0.942	0.941	0.943	0.950	0.942	0.941	0.943	0.949
0.2	7	100	0.199	0.942	0.941	0.943	0.950	0.942	0.941	0.943	0.948
0.3	7	100	0.298	0.943	0.942	0.944	0.950	0.942	0.941	0.944	0.948
0.4	7	100	0.398	0.944	0.943	0.946	0.949	0.943	0.942	0.945	0.948
0.5	7	100	0.497	0.945	0.944	0.946	0.949	0.945	0.944	0.946	0.947
0.6	7	100	0.597	0.946	0.945	0.947	0.949	0.945	0.945	0.946	0.947
0.7	7	100	0.697	0.946	0.945	0.947	0.949	0.946	0.945	0.947	0.947
0.8	7	100	0.797	0.946	0.945	0.947	0.948	0.947	0.946	0.948	0.947
0.9	7	100	0.898	0.946	0.946	0.948	0.948	0.947	0.946	0.948	0.947
0.1	8	100	0.100	0.942	0.941	0.943	0.949	0.943	0.942	0.943	0.948
0.2	8	100	0.199	0.942	0.941	0.943	0.949	0.943	0.942	0.944	0.946
0.3	8	100	0.298	0.942	0.941	0.943	0.949	0.944	0.942	0.945	0.947
0.4	8	100	0.398	0.943	0.942	0.944	0.949	0.944	0.943	0.945	0.948
0.5	8	100	0.497	0.945	0.943	0.946	0.948	0.945	0.944	0.946	0.947
0.6	8	100	0.597	0.945	0.944	0.946	0.948	0.945	0.945	0.946	0.948
0.7	8	100	0.697	0.944	0.944	0.945	0.948	0.946	0.946	0.947	0.947
0.8	8	100	0.797	0.946	0.945	0.947	0.947	0.946	0.945	0.948	0.947
0.9	8	100	0.898	0.946	0.945	0.947	0.948	0.946	0.946	0.947	0.947
0.1	9	100	0.100	0.940	0.939	0.941	0.949	0.940	0.939	0.941	0.947
0.2	9	100	0.199	0.942	0.940	0.943	0.948	0.941	0.940	0.942	0.946
0.3	9	100	0.298	0.942	0.941	0.943	0.949	0.942	0.942	0.943	0.947
0.4	9	100	0.398	0.944	0.943	0.945	0.949	0.944	0.943	0.945	0.947
0.5	9	100	0.497	0.945	0.944	0.946	0.948	0.945	0.944	0.946	0.948
0.6	9	100	0.597	0.945	0.944	0.946	0.949	0.945	0.944	0.946	0.946
0.7	9	100	0.697	0.946	0.945	0.947	0.949	0.945	0.944	0.946	0.947
0.8	9	100	0.797	0.947	0.946	0.948	0.948	0.946	0.945	0.947	0.947
0.9	9	100	0.898	0.947	0.946	0.948	0.948	0.948	0.947	0.948	0.948
0.1	10	100	0.100	0.940	0.939	0.941	0.947	0.941	0.940	0.941	0.946
0.2	10	100	0.199	0.940	0.939	0.941	0.947	0.941	0.939	0.942	0.945
0.3	10	100	0.298	0.941	0.940	0.943	0.948	0.942	0.940	0.943	0.946
0.4	10	100	0.398	0.943	0.942	0.944	0.948	0.943	0.942	0.944	0.946
0.5	10	100	0.497	0.944	0.943	0.945	0.948	0.944	0.943	0.944	0.947
0.6	10	100	0.597	0.945	0.943	0.945	0.948	0.944	0.943	0.945	0.947
0.7	10	100	0.697	0.946	0.944	0.946	0.948	0.945	0.944	0.946	0.947
0.8	10	100	0.797	0.946	0.945	0.947	0.948	0.947	0.946	0.947	0.947
0.9	10	100	0.898	0.947	0.946	0.948	0.948	0.947	0.946	0.948	0.948

0.2 Supplementary Table 2

The table shows the average width obtained from 25,000 simulations for the eight confidence interval methods under different scenarios mentioned in Section 3 of the main paper.

ρ	k	n	$E[\hat{\rho}]$	$Wald_S$	$Wald_F$	$Wald_{Ze}$	F	Z_S	Z_F	Z_{Ze}	ZF_ρ
0.1	2	20	0.095	0.837	0.827	1.013	0.814	0.794	0.784	0.938	0.794
0.2	2	20	0.190	0.815	0.805	0.987	0.796	0.776	0.767	0.918	0.776
0.3	2	20	0.286	0.778	0.768	0.942	0.764	0.745	0.736	0.884	0.745
0.4	2	20	0.383	0.725	0.716	0.878	0.719	0.701	0.693	0.835	0.701
0.5	2	20	0.481	0.656	0.648	0.794	0.659	0.642	0.634	0.769	0.642
0.6	2	20	0.580	0.569	0.562	0.688	0.581	0.566	0.559	0.682	0.566
0.7	2	20	0.681	0.462	0.456	0.559	0.483	0.470	0.464	0.572	0.470
0.8	2	20	0.784	0.334	0.330	0.405	0.359	0.349	0.344	0.430	0.349
0.9	2	20	0.890	0.182	0.179	0.220	0.203	0.197	0.194	0.246	0.197
0.1	3	20	0.095	0.535	0.526	0.583	0.531	0.523	0.514	0.567	0.515
0.2	3	20	0.192	0.554	0.545	0.605	0.545	0.542	0.533	0.588	0.531
0.3	3	20	0.288	0.556	0.547	0.607	0.543	0.544	0.535	0.591	0.533
0.4	3	20	0.385	0.540	0.531	0.589	0.526	0.531	0.522	0.577	0.519
0.5	3	20	0.483	0.505	0.497	0.551	0.493	0.500	0.492	0.544	0.489
0.6	3	20	0.581	0.451	0.444	0.492	0.443	0.451	0.444	0.492	0.442
0.7	3	20	0.682	0.377	0.370	0.411	0.374	0.382	0.375	0.417	0.375
0.8	3	20	0.785	0.279	0.274	0.304	0.281	0.288	0.283	0.316	0.284
0.9	3	20	0.891	0.155	0.152	0.169	0.160	0.164	0.161	0.181	0.163
0.1	4	20	0.097	0.413	0.405	0.436	0.420	0.407	0.399	0.429	0.402
0.2	4	20	0.193	0.450	0.442	0.476	0.450	0.443	0.435	0.468	0.435
0.3	4	20	0.290	0.468	0.459	0.495	0.462	0.461	0.453	0.487	0.450
0.4	4	20	0.386	0.467	0.458	0.494	0.457	0.461	0.453	0.487	0.450
0.5	4	20	0.484	0.447	0.438	0.472	0.435	0.443	0.435	0.468	0.432
0.6	4	20	0.582	0.406	0.398	0.429	0.396	0.406	0.398	0.429	0.396
0.7	4	20	0.683	0.344	0.337	0.363	0.337	0.348	0.341	0.368	0.340
0.8	4	20	0.785	0.258	0.253	0.272	0.256	0.265	0.260	0.281	0.261
0.9	4	20	0.891	0.145	0.142	0.153	0.146	0.153	0.149	0.162	0.151
0.1	5	20	0.097	0.346	0.339	0.360	0.358	0.342	0.335	0.356	0.340
0.2	5	20	0.194	0.393	0.385	0.410	0.398	0.389	0.381	0.404	0.382
0.3	5	20	0.291	0.421	0.412	0.438	0.418	0.415	0.407	0.432	0.406
0.4	5	20	0.387	0.428	0.419	0.446	0.420	0.423	0.415	0.441	0.412
0.5	5	20	0.485	0.415	0.407	0.432	0.405	0.413	0.404	0.429	0.401
0.6	5	20	0.583	0.382	0.374	0.398	0.371	0.382	0.374	0.398	0.372
0.7	5	20	0.683	0.326	0.319	0.340	0.318	0.330	0.323	0.344	0.322
0.8	5	20	0.785	0.246	0.241	0.257	0.243	0.253	0.248	0.264	0.249
0.9	5	20	0.891	0.139	0.137	0.145	0.140	0.146	0.143	0.153	0.145
0.1	6	20	0.098	0.304	0.297	0.314	0.319	0.301	0.295	0.311	0.300
0.2	6	20	0.195	0.358	0.350	0.369	0.366	0.354	0.347	0.365	0.349
0.3	6	20	0.291	0.391	0.383	0.404	0.391	0.387	0.379	0.399	0.378
0.4	6	20	0.388	0.403	0.395	0.417	0.398	0.400	0.391	0.412	0.389
0.5	6	20	0.485	0.395	0.387	0.408	0.386	0.393	0.385	0.406	0.382
0.6	6	20	0.584	0.366	0.359	0.378	0.356	0.367	0.359	0.379	0.357
0.7	6	20	0.684	0.315	0.308	0.325	0.307	0.319	0.312	0.329	0.311

0.8	6	20	0.786	0.239	0.234	0.247	0.235	0.246	0.240	0.254	0.241
0.9	6	20	0.891	0.136	0.133	0.140	0.135	0.143	0.139	0.148	0.141
0.1	7	20	0.098	0.274	0.268	0.282	0.291	0.273	0.267	0.280	0.273
0.2	7	20	0.195	0.333	0.326	0.342	0.343	0.330	0.323	0.339	0.326
0.3	7	20	0.292	0.370	0.362	0.380	0.373	0.367	0.359	0.376	0.359
0.4	7	20	0.389	0.386	0.378	0.397	0.382	0.383	0.375	0.393	0.373
0.5	7	20	0.486	0.382	0.374	0.392	0.374	0.380	0.372	0.390	0.369
0.6	7	20	0.584	0.356	0.348	0.366	0.346	0.356	0.349	0.366	0.346
0.7	7	20	0.684	0.308	0.301	0.316	0.299	0.311	0.304	0.319	0.303
0.8	7	20	0.786	0.235	0.229	0.241	0.229	0.241	0.235	0.247	0.236
0.9	7	20	0.891	0.134	0.131	0.137	0.133	0.140	0.137	0.144	0.138
0.1	8	20	0.098	0.253	0.247	0.259	0.270	0.251	0.246	0.257	0.252
0.2	8	20	0.195	0.315	0.308	0.322	0.326	0.312	0.306	0.319	0.309
0.3	8	20	0.292	0.355	0.347	0.363	0.359	0.352	0.344	0.360	0.345
0.4	8	20	0.389	0.374	0.366	0.383	0.371	0.371	0.363	0.379	0.362
0.5	8	20	0.486	0.372	0.364	0.381	0.365	0.370	0.362	0.379	0.360
0.6	8	20	0.584	0.348	0.341	0.356	0.339	0.349	0.341	0.357	0.339
0.7	8	20	0.684	0.302	0.295	0.309	0.294	0.305	0.298	0.313	0.298
0.8	8	20	0.786	0.231	0.226	0.236	0.226	0.237	0.231	0.243	0.232
0.9	8	20	0.891	0.132	0.129	0.135	0.131	0.138	0.135	0.142	0.137
0.1	9	20	0.098	0.236	0.231	0.241	0.255	0.235	0.230	0.240	0.237
0.2	9	20	0.196	0.301	0.294	0.307	0.314	0.299	0.292	0.305	0.296
0.3	9	20	0.292	0.344	0.336	0.351	0.349	0.341	0.333	0.347	0.334
0.4	9	20	0.389	0.365	0.357	0.372	0.363	0.362	0.354	0.369	0.353
0.5	9	20	0.486	0.365	0.356	0.372	0.358	0.363	0.355	0.370	0.353
0.6	9	20	0.584	0.343	0.335	0.350	0.334	0.343	0.335	0.350	0.333
0.7	9	20	0.684	0.298	0.291	0.304	0.289	0.301	0.294	0.307	0.293
0.8	9	20	0.786	0.229	0.223	0.233	0.223	0.234	0.229	0.239	0.229
0.9	9	20	0.891	0.131	0.128	0.134	0.129	0.137	0.134	0.140	0.135
0.1	10	20	0.098	0.223	0.218	0.227	0.242	0.222	0.217	0.226	0.224
0.2	10	20	0.196	0.290	0.284	0.295	0.303	0.288	0.282	0.293	0.286
0.3	10	20	0.292	0.335	0.327	0.341	0.340	0.332	0.325	0.338	0.326
0.4	10	20	0.389	0.357	0.349	0.364	0.356	0.355	0.347	0.361	0.346
0.5	10	20	0.486	0.359	0.351	0.365	0.352	0.357	0.349	0.363	0.347
0.6	10	20	0.584	0.338	0.331	0.344	0.329	0.339	0.331	0.345	0.329
0.7	10	20	0.684	0.295	0.288	0.300	0.286	0.298	0.291	0.303	0.290
0.8	10	20	0.786	0.227	0.221	0.231	0.221	0.232	0.226	0.236	0.227
0.9	10	20	0.891	0.130	0.127	0.132	0.128	0.136	0.132	0.138	0.134
0.1	2	30	0.096	0.692	0.686	0.783	0.678	0.667	0.661	0.746	0.667
0.2	2	30	0.193	0.673	0.667	0.761	0.662	0.650	0.645	0.728	0.650
0.3	2	30	0.291	0.641	0.635	0.725	0.633	0.622	0.617	0.698	0.622
0.4	2	30	0.389	0.596	0.591	0.674	0.593	0.582	0.577	0.654	0.582
0.5	2	30	0.487	0.536	0.532	0.607	0.539	0.529	0.525	0.596	0.529
0.6	2	30	0.587	0.463	0.459	0.523	0.470	0.462	0.458	0.522	0.462
0.7	2	30	0.688	0.374	0.370	0.423	0.386	0.379	0.375	0.430	0.379
0.8	2	30	0.790	0.268	0.266	0.303	0.282	0.277	0.274	0.316	0.277
0.9	2	30	0.894	0.144	0.143	0.163	0.155	0.152	0.151	0.175	0.152
0.1	3	30	0.097	0.440	0.435	0.466	0.438	0.433	0.428	0.458	0.428
0.2	3	30	0.195	0.456	0.451	0.483	0.450	0.449	0.444	0.474	0.443
0.3	3	30	0.292	0.457	0.452	0.484	0.450	0.450	0.445	0.476	0.444

0.4	3	30	0.390	0.443	0.438	0.469	0.435	0.438	0.433	0.463	0.431
0.5	3	30	0.489	0.413	0.408	0.437	0.406	0.410	0.405	0.434	0.404
0.6	3	30	0.588	0.367	0.363	0.388	0.363	0.367	0.363	0.389	0.362
0.7	3	30	0.688	0.304	0.301	0.322	0.303	0.307	0.304	0.326	0.304
0.8	3	30	0.790	0.223	0.221	0.236	0.225	0.229	0.226	0.243	0.227
0.9	3	30	0.894	0.123	0.121	0.130	0.126	0.128	0.126	0.136	0.127
0.1	4	30	0.098	0.339	0.335	0.352	0.343	0.336	0.331	0.348	0.333
0.2	4	30	0.196	0.370	0.365	0.384	0.370	0.366	0.361	0.379	0.361
0.3	4	30	0.294	0.384	0.380	0.399	0.381	0.380	0.376	0.394	0.374
0.4	4	30	0.392	0.383	0.378	0.397	0.377	0.380	0.375	0.393	0.373
0.5	4	30	0.490	0.365	0.360	0.378	0.358	0.363	0.358	0.376	0.357
0.6	4	30	0.589	0.330	0.326	0.342	0.324	0.330	0.326	0.343	0.325
0.7	4	30	0.689	0.277	0.274	0.288	0.274	0.280	0.276	0.291	0.276
0.8	4	30	0.791	0.206	0.204	0.214	0.205	0.210	0.208	0.219	0.208
0.9	4	30	0.894	0.114	0.113	0.119	0.116	0.119	0.117	0.124	0.118
0.1	5	30	0.099	0.284	0.280	0.291	0.291	0.282	0.278	0.289	0.280
0.2	5	30	0.197	0.323	0.319	0.332	0.326	0.320	0.316	0.329	0.317
0.3	5	30	0.294	0.345	0.341	0.355	0.344	0.342	0.338	0.352	0.337
0.4	5	30	0.392	0.351	0.346	0.360	0.346	0.348	0.343	0.357	0.342
0.5	5	30	0.490	0.339	0.335	0.348	0.333	0.338	0.333	0.347	0.331
0.6	5	30	0.589	0.310	0.306	0.319	0.305	0.311	0.306	0.319	0.305
0.7	5	30	0.689	0.263	0.260	0.271	0.259	0.266	0.262	0.273	0.261
0.8	5	30	0.791	0.197	0.195	0.203	0.195	0.201	0.198	0.207	0.199
0.9	5	30	0.894	0.110	0.109	0.113	0.110	0.114	0.112	0.117	0.113
0.1	6	30	0.099	0.249	0.245	0.254	0.257	0.247	0.244	0.253	0.247
0.2	6	30	0.197	0.293	0.289	0.300	0.298	0.291	0.287	0.298	0.289
0.3	6	30	0.295	0.321	0.316	0.328	0.321	0.318	0.314	0.325	0.314
0.4	6	30	0.393	0.330	0.326	0.338	0.327	0.328	0.324	0.335	0.323
0.5	6	30	0.491	0.323	0.319	0.330	0.318	0.322	0.317	0.329	0.316
0.6	6	30	0.590	0.298	0.294	0.304	0.292	0.298	0.294	0.305	0.293
0.7	6	30	0.690	0.255	0.251	0.260	0.250	0.257	0.253	0.262	0.252
0.8	6	30	0.791	0.192	0.189	0.196	0.189	0.195	0.192	0.200	0.193
0.9	6	30	0.894	0.108	0.106	0.110	0.107	0.111	0.110	0.114	0.110
0.1	7	30	0.099	0.224	0.221	0.228	0.234	0.223	0.220	0.227	0.224
0.2	7	30	0.197	0.273	0.269	0.278	0.278	0.271	0.267	0.276	0.269
0.3	7	30	0.295	0.304	0.299	0.309	0.305	0.302	0.297	0.307	0.297
0.4	7	30	0.393	0.317	0.312	0.322	0.314	0.315	0.310	0.320	0.309
0.5	7	30	0.491	0.312	0.308	0.318	0.307	0.311	0.306	0.316	0.305
0.6	7	30	0.590	0.290	0.285	0.295	0.284	0.290	0.286	0.295	0.284
0.7	7	30	0.690	0.249	0.245	0.253	0.244	0.251	0.247	0.255	0.246
0.8	7	30	0.791	0.188	0.185	0.191	0.185	0.191	0.188	0.195	0.189
0.9	7	30	0.894	0.106	0.104	0.108	0.105	0.109	0.108	0.111	0.108
0.1	8	30	0.099	0.207	0.204	0.210	0.216	0.206	0.203	0.209	0.206
0.2	8	30	0.197	0.258	0.254	0.262	0.264	0.257	0.253	0.261	0.255
0.3	8	30	0.295	0.291	0.287	0.296	0.293	0.289	0.285	0.294	0.286
0.4	8	30	0.393	0.307	0.302	0.311	0.305	0.305	0.300	0.309	0.300
0.5	8	30	0.491	0.304	0.300	0.309	0.300	0.303	0.299	0.308	0.297
0.6	8	30	0.590	0.284	0.279	0.288	0.278	0.284	0.280	0.288	0.278
0.7	8	30	0.690	0.244	0.241	0.248	0.240	0.246	0.242	0.250	0.242
0.8	8	30	0.791	0.185	0.182	0.188	0.182	0.188	0.186	0.191	0.186

0.9	8	30	0.894	0.105	0.103	0.106	0.104	0.108	0.106	0.110	0.107
0.1	9	30	0.099	0.193	0.190	0.196	0.203	0.193	0.190	0.195	0.193
0.2	9	30	0.197	0.247	0.243	0.250	0.254	0.246	0.242	0.249	0.244
0.3	9	30	0.295	0.282	0.278	0.285	0.284	0.280	0.276	0.284	0.277
0.4	9	30	0.393	0.299	0.294	0.303	0.298	0.297	0.293	0.301	0.292
0.5	9	30	0.491	0.298	0.293	0.302	0.294	0.297	0.293	0.301	0.291
0.6	9	30	0.590	0.279	0.275	0.283	0.274	0.279	0.275	0.283	0.274
0.7	9	30	0.690	0.241	0.237	0.244	0.236	0.243	0.239	0.246	0.239
0.8	9	30	0.791	0.183	0.180	0.186	0.180	0.186	0.183	0.189	0.184
0.9	9	30	0.894	0.104	0.102	0.105	0.103	0.107	0.105	0.108	0.106
0.1	10	30	0.099	0.182	0.180	0.185	0.193	0.182	0.179	0.184	0.183
0.2	10	30	0.197	0.238	0.234	0.240	0.245	0.237	0.233	0.239	0.235
0.3	10	30	0.295	0.274	0.270	0.278	0.277	0.273	0.269	0.276	0.269
0.4	10	30	0.393	0.293	0.288	0.296	0.292	0.291	0.287	0.295	0.286
0.5	10	30	0.491	0.293	0.289	0.297	0.290	0.292	0.288	0.296	0.287
0.6	10	30	0.590	0.275	0.271	0.278	0.270	0.275	0.271	0.279	0.270
0.7	10	30	0.690	0.238	0.235	0.241	0.234	0.240	0.236	0.243	0.236
0.8	10	30	0.791	0.182	0.179	0.184	0.178	0.185	0.182	0.187	0.182
0.9	10	30	0.894	0.103	0.101	0.104	0.102	0.106	0.104	0.107	0.105
0.1	2	40	0.097	0.603	0.599	0.661	0.594	0.586	0.582	0.638	0.586
0.2	2	40	0.195	0.586	0.582	0.642	0.578	0.571	0.567	0.622	0.571
0.3	2	40	0.293	0.558	0.554	0.611	0.552	0.545	0.542	0.594	0.545
0.4	2	40	0.391	0.517	0.514	0.567	0.515	0.508	0.505	0.555	0.508
0.5	2	40	0.490	0.465	0.462	0.509	0.467	0.460	0.458	0.503	0.460
0.6	2	40	0.590	0.400	0.397	0.438	0.405	0.400	0.397	0.438	0.400
0.7	2	40	0.691	0.322	0.320	0.352	0.330	0.325	0.323	0.357	0.325
0.8	2	40	0.793	0.230	0.228	0.252	0.239	0.236	0.234	0.259	0.236
0.9	2	40	0.896	0.123	0.122	0.135	0.130	0.128	0.127	0.142	0.128
0.1	3	40	0.098	0.382	0.379	0.399	0.381	0.378	0.375	0.394	0.375
0.2	3	40	0.196	0.396	0.393	0.414	0.393	0.392	0.388	0.408	0.388
0.3	3	40	0.294	0.397	0.394	0.414	0.392	0.393	0.389	0.409	0.388
0.4	3	40	0.393	0.384	0.381	0.401	0.379	0.381	0.378	0.397	0.376
0.5	3	40	0.492	0.358	0.355	0.373	0.353	0.356	0.353	0.371	0.352
0.6	3	40	0.591	0.317	0.314	0.331	0.314	0.317	0.315	0.331	0.314
0.7	3	40	0.692	0.262	0.260	0.273	0.261	0.264	0.262	0.276	0.262
0.8	3	40	0.793	0.191	0.190	0.200	0.193	0.195	0.193	0.204	0.194
0.9	3	40	0.896	0.105	0.104	0.109	0.107	0.108	0.107	0.113	0.108
0.1	4	40	0.098	0.294	0.291	0.302	0.296	0.292	0.289	0.300	0.290
0.2	4	40	0.196	0.321	0.318	0.330	0.321	0.318	0.315	0.327	0.315
0.3	4	40	0.295	0.334	0.331	0.343	0.331	0.331	0.328	0.340	0.327
0.4	4	40	0.393	0.332	0.329	0.342	0.328	0.330	0.327	0.339	0.326
0.5	4	40	0.492	0.316	0.313	0.325	0.312	0.315	0.312	0.324	0.311
0.6	4	40	0.591	0.285	0.283	0.293	0.282	0.286	0.283	0.294	0.282
0.7	4	40	0.692	0.239	0.237	0.246	0.237	0.241	0.239	0.248	0.238
0.8	4	40	0.793	0.177	0.175	0.182	0.177	0.180	0.178	0.185	0.178
0.9	4	40	0.896	0.098	0.097	0.101	0.098	0.101	0.100	0.103	0.100
0.1	5	40	0.098	0.246	0.243	0.251	0.250	0.244	0.242	0.249	0.243
0.2	5	40	0.197	0.280	0.277	0.286	0.282	0.278	0.276	0.284	0.276
0.3	5	40	0.295	0.300	0.297	0.306	0.299	0.298	0.295	0.304	0.294
0.4	5	40	0.393	0.304	0.301	0.310	0.301	0.303	0.300	0.309	0.298

0.5	5	40	0.492	0.294	0.291	0.300	0.290	0.293	0.290	0.299	0.289
0.6	5	40	0.592	0.268	0.266	0.274	0.265	0.269	0.266	0.274	0.265
0.7	5	40	0.692	0.227	0.225	0.232	0.224	0.229	0.226	0.233	0.226
0.8	5	40	0.793	0.169	0.168	0.173	0.168	0.172	0.170	0.176	0.170
0.9	5	40	0.896	0.094	0.093	0.096	0.094	0.097	0.096	0.099	0.096
0.1	6	40	0.099	0.215	0.213	0.219	0.221	0.214	0.212	0.218	0.214
0.2	6	40	0.197	0.254	0.252	0.259	0.257	0.253	0.251	0.257	0.251
0.3	6	40	0.296	0.278	0.275	0.283	0.278	0.277	0.274	0.281	0.274
0.4	6	40	0.394	0.287	0.284	0.291	0.285	0.285	0.282	0.290	0.281
0.5	6	40	0.493	0.280	0.277	0.284	0.277	0.279	0.276	0.284	0.275
0.6	6	40	0.592	0.258	0.255	0.262	0.254	0.258	0.255	0.262	0.254
0.7	6	40	0.692	0.219	0.217	0.223	0.217	0.221	0.219	0.224	0.218
0.8	6	40	0.793	0.165	0.163	0.167	0.163	0.167	0.165	0.170	0.165
0.9	6	40	0.896	0.092	0.091	0.093	0.092	0.094	0.093	0.096	0.094
0.1	7	40	0.099	0.194	0.192	0.197	0.200	0.194	0.192	0.196	0.194
0.2	7	40	0.197	0.237	0.234	0.240	0.240	0.236	0.233	0.239	0.234
0.3	7	40	0.296	0.263	0.261	0.267	0.264	0.262	0.259	0.266	0.259
0.4	7	40	0.394	0.275	0.272	0.278	0.273	0.273	0.270	0.277	0.270
0.5	7	40	0.493	0.270	0.267	0.274	0.267	0.270	0.267	0.273	0.266
0.6	7	40	0.592	0.250	0.248	0.254	0.247	0.251	0.248	0.254	0.247
0.7	7	40	0.692	0.214	0.212	0.217	0.211	0.216	0.213	0.219	0.213
0.8	7	40	0.793	0.161	0.160	0.164	0.160	0.164	0.162	0.166	0.162
0.9	7	40	0.896	0.090	0.089	0.092	0.090	0.093	0.092	0.094	0.092
0.1	8	40	0.099	0.179	0.177	0.181	0.185	0.178	0.176	0.180	0.179
0.2	8	40	0.197	0.224	0.221	0.226	0.228	0.223	0.220	0.225	0.222
0.3	8	40	0.296	0.253	0.250	0.255	0.254	0.251	0.249	0.254	0.249
0.4	8	40	0.394	0.266	0.263	0.269	0.265	0.265	0.262	0.268	0.261
0.5	8	40	0.493	0.263	0.261	0.266	0.261	0.263	0.260	0.266	0.259
0.6	8	40	0.592	0.245	0.242	0.248	0.242	0.245	0.243	0.248	0.242
0.7	8	40	0.692	0.211	0.208	0.213	0.208	0.212	0.210	0.214	0.209
0.8	8	40	0.793	0.159	0.157	0.161	0.157	0.161	0.159	0.163	0.160
0.9	8	40	0.896	0.089	0.088	0.090	0.089	0.091	0.090	0.093	0.091
0.1	9	40	0.099	0.167	0.165	0.169	0.174	0.167	0.165	0.168	0.167
0.2	9	40	0.198	0.214	0.211	0.216	0.218	0.213	0.211	0.215	0.212
0.3	9	40	0.296	0.244	0.242	0.247	0.246	0.243	0.241	0.246	0.241
0.4	9	40	0.394	0.259	0.256	0.262	0.258	0.258	0.255	0.261	0.255
0.5	9	40	0.493	0.258	0.255	0.261	0.256	0.258	0.255	0.260	0.254
0.6	9	40	0.592	0.241	0.238	0.244	0.238	0.241	0.239	0.244	0.238
0.7	9	40	0.692	0.208	0.205	0.210	0.205	0.209	0.207	0.211	0.206
0.8	9	40	0.793	0.157	0.155	0.159	0.155	0.159	0.157	0.161	0.158
0.9	9	40	0.896	0.089	0.088	0.089	0.088	0.091	0.089	0.091	0.090
0.1	10	40	0.099	0.158	0.156	0.159	0.164	0.158	0.156	0.159	0.158
0.2	10	40	0.198	0.206	0.204	0.208	0.211	0.205	0.203	0.207	0.204
0.3	10	40	0.296	0.238	0.235	0.240	0.240	0.237	0.234	0.239	0.235
0.4	10	40	0.394	0.254	0.251	0.256	0.253	0.253	0.250	0.255	0.250
0.5	10	40	0.493	0.254	0.251	0.256	0.252	0.253	0.251	0.256	0.250
0.6	10	40	0.592	0.238	0.235	0.240	0.235	0.238	0.235	0.240	0.235
0.7	10	40	0.692	0.206	0.203	0.207	0.203	0.207	0.204	0.209	0.204
0.8	10	40	0.793	0.156	0.154	0.157	0.154	0.158	0.156	0.159	0.156
0.9	10	40	0.896	0.088	0.087	0.089	0.087	0.090	0.089	0.091	0.089

0.1	2	50	0.098	0.541	0.538	0.582	0.534	0.528	0.526	0.566	0.528
0.2	2	50	0.196	0.526	0.523	0.565	0.520	0.514	0.512	0.551	0.514
0.3	2	50	0.294	0.500	0.497	0.537	0.496	0.491	0.488	0.526	0.491
0.4	2	50	0.393	0.463	0.461	0.498	0.462	0.457	0.455	0.490	0.457
0.5	2	50	0.492	0.416	0.414	0.447	0.417	0.413	0.411	0.443	0.413
0.6	2	50	0.592	0.357	0.355	0.384	0.361	0.357	0.355	0.384	0.357
0.7	2	50	0.693	0.287	0.285	0.308	0.293	0.290	0.288	0.312	0.290
0.8	2	50	0.794	0.204	0.203	0.220	0.211	0.209	0.208	0.225	0.209
0.9	2	50	0.896	0.109	0.108	0.117	0.114	0.113	0.112	0.122	0.113
0.1	3	50	0.099	0.343	0.340	0.355	0.342	0.340	0.337	0.351	0.337
0.2	3	50	0.197	0.355	0.353	0.368	0.353	0.352	0.350	0.364	0.349
0.3	3	50	0.296	0.356	0.353	0.368	0.352	0.353	0.350	0.364	0.349
0.4	3	50	0.394	0.344	0.342	0.356	0.340	0.342	0.339	0.353	0.338
0.5	3	50	0.493	0.320	0.318	0.331	0.317	0.319	0.316	0.329	0.316
0.6	3	50	0.593	0.283	0.281	0.293	0.281	0.283	0.281	0.293	0.281
0.7	3	50	0.693	0.233	0.232	0.242	0.233	0.235	0.234	0.243	0.234
0.8	3	50	0.794	0.170	0.169	0.176	0.171	0.173	0.172	0.179	0.172
0.9	3	50	0.897	0.093	0.092	0.096	0.094	0.095	0.094	0.099	0.095
0.1	4	50	0.099	0.263	0.261	0.269	0.265	0.262	0.260	0.268	0.261
0.2	4	50	0.197	0.288	0.286	0.294	0.287	0.286	0.284	0.292	0.284
0.3	4	50	0.296	0.299	0.297	0.306	0.297	0.297	0.295	0.304	0.294
0.4	4	50	0.395	0.297	0.295	0.304	0.295	0.296	0.294	0.302	0.293
0.5	4	50	0.494	0.283	0.281	0.289	0.280	0.282	0.280	0.288	0.279
0.6	4	50	0.593	0.255	0.253	0.261	0.252	0.255	0.253	0.261	0.253
0.7	4	50	0.693	0.213	0.212	0.218	0.212	0.215	0.213	0.219	0.213
0.8	4	50	0.794	0.157	0.156	0.161	0.157	0.160	0.158	0.163	0.159
0.9	4	50	0.897	0.087	0.086	0.089	0.087	0.089	0.088	0.091	0.088
0.1	5	50	0.099	0.220	0.218	0.224	0.223	0.219	0.217	0.223	0.219
0.2	5	50	0.198	0.251	0.249	0.255	0.252	0.250	0.248	0.254	0.248
0.3	5	50	0.296	0.268	0.266	0.273	0.268	0.267	0.265	0.271	0.264
0.4	5	50	0.395	0.272	0.270	0.277	0.270	0.271	0.269	0.276	0.268
0.5	5	50	0.494	0.263	0.261	0.267	0.260	0.262	0.260	0.266	0.259
0.6	5	50	0.593	0.240	0.238	0.244	0.237	0.240	0.238	0.244	0.237
0.7	5	50	0.694	0.202	0.201	0.206	0.201	0.204	0.202	0.207	0.202
0.8	5	50	0.795	0.151	0.149	0.153	0.150	0.152	0.151	0.155	0.151
0.9	5	50	0.897	0.083	0.083	0.085	0.084	0.085	0.085	0.087	0.085
0.1	6	50	0.099	0.193	0.191	0.195	0.197	0.192	0.191	0.195	0.192
0.2	6	50	0.198	0.228	0.226	0.231	0.230	0.227	0.225	0.230	0.226
0.3	6	50	0.297	0.249	0.247	0.252	0.249	0.248	0.246	0.251	0.246
0.4	6	50	0.395	0.257	0.254	0.260	0.255	0.256	0.254	0.259	0.253
0.5	6	50	0.494	0.250	0.248	0.254	0.248	0.250	0.248	0.253	0.247
0.6	6	50	0.594	0.230	0.228	0.233	0.228	0.230	0.228	0.233	0.228
0.7	6	50	0.694	0.196	0.194	0.198	0.194	0.197	0.195	0.199	0.195
0.8	6	50	0.795	0.146	0.145	0.148	0.145	0.148	0.147	0.150	0.147
0.9	6	50	0.897	0.082	0.081	0.083	0.081	0.083	0.082	0.084	0.083
0.1	7	50	0.099	0.174	0.172	0.176	0.178	0.173	0.172	0.175	0.174
0.2	7	50	0.198	0.212	0.210	0.214	0.215	0.211	0.209	0.213	0.210
0.3	7	50	0.297	0.236	0.234	0.238	0.236	0.235	0.233	0.237	0.233
0.4	7	50	0.395	0.246	0.244	0.248	0.245	0.245	0.243	0.248	0.242
0.5	7	50	0.494	0.242	0.240	0.244	0.240	0.241	0.239	0.244	0.239

0.6	7	50	0.594	0.224	0.222	0.226	0.221	0.224	0.222	0.226	0.221
0.7	7	50	0.694	0.191	0.189	0.193	0.189	0.192	0.190	0.194	0.190
0.8	7	50	0.795	0.144	0.142	0.145	0.142	0.145	0.144	0.147	0.144
0.9	7	50	0.897	0.080	0.079	0.081	0.080	0.082	0.081	0.083	0.081
0.1	8	50	0.099	0.160	0.159	0.162	0.165	0.160	0.158	0.161	0.160
0.2	8	50	0.198	0.200	0.199	0.202	0.203	0.200	0.198	0.201	0.199
0.3	8	50	0.297	0.226	0.224	0.228	0.227	0.225	0.223	0.227	0.224
0.4	8	50	0.395	0.238	0.236	0.240	0.237	0.237	0.235	0.239	0.235
0.5	8	50	0.494	0.236	0.234	0.238	0.234	0.235	0.233	0.237	0.232
0.6	8	50	0.594	0.219	0.217	0.221	0.217	0.219	0.217	0.221	0.217
0.7	8	50	0.694	0.188	0.186	0.190	0.186	0.189	0.187	0.190	0.187
0.8	8	50	0.795	0.141	0.140	0.143	0.140	0.143	0.142	0.144	0.142
0.9	8	50	0.897	0.079	0.079	0.080	0.079	0.081	0.080	0.081	0.080
0.1	9	50	0.099	0.150	0.148	0.151	0.154	0.149	0.148	0.150	0.150
0.2	9	50	0.198	0.191	0.190	0.193	0.195	0.191	0.189	0.192	0.190
0.3	9	50	0.297	0.219	0.217	0.221	0.220	0.218	0.216	0.220	0.216
0.4	9	50	0.396	0.232	0.230	0.234	0.231	0.231	0.229	0.233	0.229
0.5	9	50	0.494	0.231	0.229	0.233	0.229	0.230	0.228	0.232	0.228
0.6	9	50	0.594	0.215	0.214	0.217	0.213	0.216	0.214	0.217	0.213
0.7	9	50	0.694	0.185	0.184	0.187	0.183	0.186	0.184	0.188	0.184
0.8	9	50	0.795	0.140	0.139	0.141	0.139	0.141	0.140	0.142	0.140
0.9	9	50	0.897	0.078	0.078	0.079	0.078	0.080	0.079	0.081	0.080
0.1	10	50	0.099	0.141	0.140	0.142	0.146	0.141	0.140	0.142	0.142
0.2	10	50	0.198	0.184	0.183	0.186	0.188	0.184	0.182	0.185	0.183
0.3	10	50	0.297	0.213	0.211	0.215	0.215	0.212	0.210	0.214	0.211
0.4	10	50	0.396	0.227	0.225	0.229	0.227	0.227	0.225	0.228	0.224
0.5	10	50	0.494	0.227	0.225	0.229	0.225	0.227	0.225	0.228	0.224
0.6	10	50	0.594	0.213	0.211	0.214	0.210	0.213	0.211	0.214	0.210
0.7	10	50	0.694	0.183	0.182	0.185	0.181	0.184	0.182	0.185	0.182
0.8	10	50	0.795	0.139	0.137	0.140	0.137	0.140	0.139	0.141	0.139
0.9	10	50	0.897	0.078	0.077	0.078	0.077	0.079	0.079	0.080	0.079
0.1	2	60	0.099	0.495	0.493	0.526	0.490	0.485	0.483	0.514	0.485
0.2	2	60	0.198	0.481	0.479	0.510	0.476	0.472	0.470	0.500	0.472
0.3	2	60	0.296	0.457	0.455	0.485	0.454	0.450	0.448	0.477	0.450
0.4	2	60	0.395	0.423	0.421	0.449	0.422	0.418	0.416	0.443	0.418
0.5	2	60	0.495	0.379	0.377	0.402	0.380	0.377	0.375	0.400	0.377
0.6	2	60	0.594	0.325	0.324	0.345	0.328	0.325	0.324	0.345	0.325
0.7	2	60	0.695	0.261	0.260	0.277	0.265	0.263	0.262	0.279	0.263
0.8	2	60	0.796	0.185	0.185	0.197	0.191	0.189	0.188	0.201	0.189
0.9	2	60	0.897	0.099	0.098	0.105	0.103	0.102	0.101	0.108	0.102
0.1	3	60	0.099	0.313	0.312	0.322	0.312	0.311	0.309	0.320	0.309
0.2	3	60	0.198	0.325	0.323	0.334	0.323	0.322	0.320	0.331	0.320
0.3	3	60	0.297	0.325	0.323	0.334	0.322	0.323	0.321	0.332	0.320
0.4	3	60	0.396	0.314	0.313	0.323	0.311	0.312	0.311	0.321	0.310
0.5	3	60	0.495	0.292	0.290	0.300	0.290	0.291	0.289	0.299	0.289
0.6	3	60	0.595	0.258	0.257	0.266	0.257	0.258	0.257	0.266	0.257
0.7	3	60	0.695	0.213	0.211	0.219	0.212	0.214	0.213	0.220	0.213
0.8	3	60	0.796	0.155	0.154	0.159	0.155	0.157	0.156	0.161	0.156
0.9	3	60	0.897	0.084	0.084	0.086	0.085	0.086	0.085	0.088	0.086
0.1	4	60	0.099	0.241	0.239	0.245	0.242	0.239	0.238	0.244	0.238

0.2	4	60	0.198	0.263	0.261	0.268	0.263	0.262	0.260	0.266	0.260
0.3	4	60	0.297	0.273	0.272	0.278	0.272	0.272	0.270	0.277	0.270
0.4	4	60	0.396	0.272	0.270	0.277	0.270	0.271	0.269	0.275	0.268
0.5	4	60	0.495	0.258	0.257	0.263	0.256	0.258	0.256	0.262	0.255
0.6	4	60	0.595	0.232	0.231	0.237	0.230	0.233	0.231	0.237	0.231
0.7	4	60	0.695	0.194	0.193	0.198	0.193	0.195	0.194	0.199	0.194
0.8	4	60	0.795	0.143	0.142	0.146	0.143	0.145	0.144	0.147	0.144
0.9	4	60	0.897	0.079	0.078	0.080	0.079	0.080	0.080	0.082	0.080
0.1	5	60	0.099	0.201	0.200	0.204	0.203	0.200	0.199	0.203	0.200
0.2	5	60	0.198	0.229	0.228	0.232	0.230	0.228	0.227	0.231	0.227
0.3	5	60	0.297	0.245	0.244	0.249	0.245	0.244	0.243	0.247	0.242
0.4	5	60	0.396	0.249	0.247	0.252	0.247	0.248	0.246	0.251	0.246
0.5	5	60	0.495	0.240	0.238	0.243	0.238	0.239	0.238	0.243	0.237
0.6	5	60	0.595	0.219	0.217	0.222	0.217	0.219	0.217	0.222	0.217
0.7	5	60	0.695	0.184	0.183	0.187	0.183	0.185	0.184	0.188	0.184
0.8	5	60	0.795	0.137	0.136	0.139	0.136	0.138	0.137	0.140	0.138
0.9	5	60	0.897	0.076	0.075	0.077	0.076	0.077	0.077	0.078	0.077
0.1	6	60	0.099	0.176	0.175	0.178	0.179	0.176	0.174	0.177	0.175
0.2	6	60	0.198	0.208	0.207	0.210	0.210	0.208	0.206	0.210	0.206
0.3	6	60	0.297	0.228	0.226	0.230	0.228	0.227	0.225	0.229	0.225
0.4	6	60	0.396	0.234	0.233	0.237	0.233	0.234	0.232	0.236	0.232
0.5	6	60	0.495	0.229	0.227	0.231	0.227	0.228	0.227	0.231	0.226
0.6	6	60	0.595	0.210	0.208	0.212	0.208	0.210	0.209	0.212	0.208
0.7	6	60	0.695	0.178	0.177	0.180	0.177	0.179	0.178	0.181	0.178
0.8	6	60	0.796	0.133	0.132	0.135	0.132	0.134	0.133	0.136	0.134
0.9	6	60	0.897	0.074	0.073	0.075	0.074	0.075	0.075	0.076	0.075
0.1	7	60	0.100	0.159	0.158	0.160	0.162	0.159	0.157	0.160	0.159
0.2	7	60	0.199	0.194	0.192	0.195	0.196	0.193	0.192	0.195	0.192
0.3	7	60	0.298	0.216	0.214	0.217	0.216	0.215	0.213	0.217	0.213
0.4	7	60	0.397	0.225	0.223	0.227	0.224	0.224	0.222	0.226	0.222
0.5	7	60	0.496	0.221	0.219	0.223	0.219	0.220	0.219	0.222	0.218
0.6	7	60	0.595	0.204	0.203	0.206	0.202	0.204	0.203	0.206	0.202
0.7	7	60	0.695	0.174	0.173	0.176	0.172	0.175	0.173	0.176	0.173
0.8	7	60	0.796	0.130	0.130	0.132	0.130	0.132	0.131	0.133	0.131
0.9	7	60	0.897	0.073	0.072	0.073	0.073	0.074	0.073	0.075	0.074
0.1	8	60	0.100	0.146	0.145	0.147	0.150	0.146	0.145	0.147	0.146
0.2	8	60	0.199	0.183	0.182	0.184	0.185	0.183	0.181	0.184	0.182
0.3	8	60	0.298	0.207	0.205	0.208	0.207	0.206	0.205	0.208	0.205
0.4	8	60	0.397	0.217	0.216	0.219	0.217	0.217	0.215	0.218	0.215
0.5	8	60	0.496	0.215	0.214	0.217	0.214	0.215	0.213	0.216	0.213
0.6	8	60	0.595	0.200	0.198	0.201	0.198	0.200	0.198	0.201	0.198
0.7	8	60	0.695	0.171	0.170	0.172	0.169	0.172	0.170	0.173	0.170
0.8	8	60	0.796	0.129	0.128	0.130	0.128	0.130	0.129	0.131	0.129
0.9	8	60	0.897	0.072	0.071	0.072	0.072	0.073	0.072	0.074	0.073
0.1	9	60	0.100	0.137	0.136	0.138	0.140	0.136	0.135	0.137	0.137
0.2	9	60	0.199	0.175	0.174	0.176	0.177	0.175	0.173	0.176	0.174
0.3	9	60	0.298	0.200	0.198	0.201	0.201	0.199	0.198	0.201	0.198
0.4	9	60	0.396	0.212	0.210	0.213	0.211	0.211	0.210	0.213	0.209
0.5	9	60	0.496	0.211	0.209	0.212	0.209	0.210	0.209	0.212	0.208
0.6	9	60	0.595	0.196	0.195	0.198	0.195	0.197	0.195	0.198	0.195

0.7	9	60	0.695	0.169	0.167	0.170	0.167	0.169	0.168	0.171	0.168
0.8	9	60	0.796	0.127	0.126	0.128	0.126	0.128	0.127	0.129	0.127
0.9	9	60	0.897	0.071	0.071	0.072	0.071	0.072	0.072	0.073	0.072
0.1	10	60	0.100	0.129	0.128	0.130	0.133	0.129	0.128	0.130	0.129
0.2	10	60	0.199	0.169	0.167	0.170	0.171	0.168	0.167	0.169	0.168
0.3	10	60	0.298	0.195	0.193	0.196	0.196	0.194	0.193	0.195	0.193
0.4	10	60	0.397	0.208	0.206	0.209	0.207	0.207	0.206	0.208	0.205
0.5	10	60	0.496	0.207	0.206	0.209	0.206	0.207	0.206	0.208	0.205
0.6	10	60	0.595	0.194	0.192	0.195	0.192	0.194	0.193	0.195	0.192
0.7	10	60	0.695	0.167	0.166	0.168	0.165	0.168	0.166	0.169	0.166
0.8	10	60	0.796	0.126	0.125	0.127	0.125	0.127	0.126	0.128	0.126
0.9	10	60	0.897	0.071	0.070	0.071	0.070	0.072	0.071	0.072	0.071
0.1	2	70	0.099	0.459	0.457	0.483	0.455	0.451	0.450	0.474	0.451
0.2	2	70	0.197	0.446	0.444	0.469	0.442	0.439	0.437	0.461	0.439
0.3	2	70	0.296	0.423	0.422	0.446	0.421	0.418	0.416	0.439	0.418
0.4	2	70	0.395	0.392	0.390	0.412	0.391	0.388	0.387	0.408	0.388
0.5	2	70	0.495	0.351	0.350	0.370	0.352	0.349	0.348	0.368	0.349
0.6	2	70	0.595	0.301	0.300	0.317	0.304	0.301	0.300	0.317	0.301
0.7	2	70	0.695	0.241	0.240	0.254	0.245	0.243	0.242	0.256	0.243
0.8	2	70	0.796	0.171	0.171	0.180	0.175	0.174	0.173	0.183	0.174
0.9	2	70	0.898	0.091	0.091	0.096	0.094	0.093	0.093	0.099	0.093
0.1	3	70	0.099	0.290	0.289	0.298	0.290	0.288	0.287	0.295	0.287
0.2	3	70	0.198	0.301	0.300	0.308	0.299	0.299	0.298	0.306	0.297
0.3	3	70	0.297	0.301	0.300	0.309	0.299	0.299	0.298	0.307	0.297
0.4	3	70	0.396	0.291	0.290	0.298	0.289	0.290	0.288	0.297	0.288
0.5	3	70	0.496	0.270	0.269	0.277	0.268	0.270	0.268	0.276	0.268
0.6	3	70	0.595	0.239	0.238	0.245	0.238	0.239	0.238	0.245	0.238
0.7	3	70	0.695	0.197	0.196	0.201	0.196	0.198	0.197	0.202	0.197
0.8	3	70	0.796	0.143	0.142	0.146	0.143	0.144	0.144	0.148	0.144
0.9	3	70	0.898	0.078	0.077	0.079	0.078	0.079	0.079	0.081	0.079
0.1	4	70	0.099	0.223	0.222	0.226	0.224	0.222	0.221	0.225	0.221
0.2	4	70	0.198	0.244	0.242	0.247	0.243	0.242	0.241	0.246	0.241
0.3	4	70	0.297	0.253	0.252	0.257	0.252	0.252	0.251	0.256	0.250
0.4	4	70	0.396	0.252	0.250	0.256	0.250	0.251	0.249	0.255	0.249
0.5	4	70	0.496	0.239	0.238	0.243	0.237	0.239	0.237	0.242	0.237
0.6	4	70	0.595	0.215	0.214	0.218	0.213	0.215	0.214	0.219	0.214
0.7	4	70	0.695	0.180	0.179	0.182	0.179	0.180	0.179	0.183	0.179
0.8	4	70	0.796	0.132	0.131	0.134	0.132	0.133	0.133	0.136	0.133
0.9	4	70	0.898	0.073	0.072	0.074	0.073	0.074	0.073	0.075	0.074
0.1	5	70	0.099	0.186	0.185	0.188	0.188	0.186	0.184	0.188	0.185
0.2	5	70	0.198	0.212	0.211	0.215	0.213	0.212	0.210	0.214	0.211
0.3	5	70	0.297	0.227	0.226	0.230	0.227	0.226	0.225	0.229	0.225
0.4	5	70	0.396	0.230	0.229	0.233	0.229	0.230	0.228	0.232	0.228
0.5	5	70	0.496	0.222	0.221	0.225	0.221	0.222	0.221	0.224	0.220
0.6	5	70	0.595	0.202	0.201	0.205	0.201	0.202	0.201	0.205	0.201
0.7	5	70	0.695	0.171	0.170	0.172	0.169	0.171	0.170	0.173	0.170
0.8	5	70	0.796	0.127	0.126	0.128	0.126	0.128	0.127	0.129	0.127
0.9	5	70	0.898	0.070	0.069	0.071	0.070	0.071	0.070	0.072	0.071
0.1	6	70	0.099	0.163	0.162	0.164	0.165	0.163	0.162	0.164	0.163
0.2	6	70	0.199	0.193	0.192	0.195	0.194	0.192	0.191	0.194	0.191

0.3	6	70	0.298	0.211	0.210	0.213	0.211	0.210	0.209	0.212	0.209
0.4	6	70	0.397	0.217	0.216	0.219	0.216	0.217	0.215	0.219	0.215
0.5	6	70	0.496	0.212	0.210	0.214	0.210	0.211	0.210	0.213	0.210
0.6	6	70	0.596	0.194	0.193	0.196	0.193	0.194	0.193	0.196	0.193
0.7	6	70	0.696	0.165	0.164	0.166	0.164	0.165	0.164	0.167	0.164
0.8	6	70	0.796	0.123	0.122	0.124	0.122	0.124	0.123	0.125	0.123
0.9	6	70	0.898	0.068	0.068	0.069	0.068	0.069	0.069	0.070	0.069
0.1	7	70	0.099	0.147	0.146	0.148	0.150	0.147	0.146	0.148	0.147
0.2	7	70	0.199	0.179	0.178	0.181	0.181	0.179	0.178	0.180	0.178
0.3	7	70	0.298	0.200	0.198	0.201	0.200	0.199	0.198	0.201	0.198
0.4	7	70	0.397	0.208	0.207	0.210	0.207	0.207	0.206	0.209	0.206
0.5	7	70	0.496	0.204	0.203	0.206	0.203	0.204	0.203	0.206	0.202
0.6	7	70	0.596	0.189	0.188	0.190	0.187	0.189	0.188	0.190	0.187
0.7	7	70	0.696	0.161	0.160	0.162	0.160	0.162	0.161	0.163	0.160
0.8	7	70	0.796	0.121	0.120	0.121	0.120	0.121	0.121	0.122	0.121
0.9	7	70	0.898	0.067	0.067	0.068	0.067	0.068	0.068	0.069	0.068
0.1	8	70	0.099	0.135	0.134	0.136	0.138	0.135	0.134	0.136	0.135
0.2	8	70	0.199	0.169	0.168	0.171	0.171	0.169	0.168	0.170	0.169
0.3	8	70	0.298	0.191	0.190	0.193	0.192	0.191	0.190	0.192	0.190
0.4	8	70	0.397	0.201	0.200	0.203	0.201	0.201	0.200	0.202	0.199
0.5	8	70	0.496	0.199	0.198	0.200	0.198	0.199	0.198	0.200	0.197
0.6	8	70	0.596	0.185	0.184	0.186	0.183	0.185	0.184	0.186	0.183
0.7	8	70	0.696	0.158	0.157	0.159	0.157	0.159	0.158	0.160	0.158
0.8	8	70	0.796	0.119	0.118	0.120	0.118	0.120	0.119	0.120	0.119
0.9	8	70	0.898	0.066	0.066	0.067	0.066	0.067	0.067	0.068	0.067
0.1	9	70	0.100	0.126	0.126	0.127	0.129	0.126	0.125	0.127	0.127
0.2	9	70	0.199	0.162	0.161	0.163	0.164	0.162	0.161	0.163	0.161
0.3	9	70	0.298	0.185	0.184	0.186	0.186	0.185	0.184	0.186	0.184
0.4	9	70	0.397	0.196	0.195	0.197	0.196	0.196	0.195	0.197	0.194
0.5	9	70	0.496	0.195	0.194	0.196	0.194	0.195	0.194	0.196	0.193
0.6	9	70	0.596	0.182	0.181	0.183	0.180	0.182	0.181	0.183	0.180
0.7	9	70	0.696	0.156	0.155	0.157	0.155	0.157	0.156	0.157	0.155
0.8	9	70	0.796	0.117	0.117	0.118	0.117	0.118	0.118	0.119	0.118
0.9	9	70	0.898	0.066	0.065	0.066	0.065	0.067	0.066	0.067	0.066
0.1	10	70	0.100	0.119	0.119	0.120	0.122	0.119	0.119	0.120	0.120
0.2	10	70	0.199	0.156	0.155	0.157	0.158	0.156	0.155	0.157	0.155
0.3	10	70	0.298	0.180	0.179	0.181	0.181	0.180	0.179	0.181	0.179
0.4	10	70	0.397	0.192	0.191	0.193	0.192	0.192	0.191	0.193	0.190
0.5	10	70	0.496	0.192	0.191	0.193	0.191	0.192	0.191	0.193	0.190
0.6	10	70	0.596	0.179	0.178	0.180	0.178	0.180	0.178	0.180	0.178
0.7	10	70	0.696	0.154	0.153	0.155	0.153	0.155	0.154	0.156	0.154
0.8	10	70	0.796	0.116	0.116	0.117	0.116	0.117	0.116	0.118	0.117
0.9	10	70	0.898	0.065	0.065	0.065	0.065	0.066	0.066	0.066	0.066
0.1	2	80	0.098	0.430	0.429	0.450	0.427	0.424	0.422	0.442	0.424
0.2	2	80	0.197	0.418	0.416	0.437	0.415	0.412	0.411	0.430	0.412
0.3	2	80	0.296	0.397	0.395	0.415	0.395	0.392	0.391	0.409	0.392
0.4	2	80	0.396	0.367	0.366	0.384	0.366	0.364	0.363	0.380	0.364
0.5	2	80	0.495	0.329	0.328	0.344	0.330	0.327	0.326	0.342	0.327
0.6	2	80	0.595	0.282	0.281	0.295	0.284	0.282	0.281	0.295	0.282
0.7	2	80	0.695	0.226	0.225	0.236	0.229	0.227	0.226	0.238	0.227

0.8	2	80	0.796	0.160	0.160	0.167	0.163	0.162	0.162	0.170	0.162
0.9	2	80	0.898	0.085	0.085	0.089	0.088	0.087	0.087	0.091	0.087
0.1	3	80	0.099	0.272	0.271	0.278	0.271	0.270	0.269	0.276	0.269
0.2	3	80	0.198	0.282	0.281	0.288	0.280	0.280	0.279	0.286	0.279
0.3	3	80	0.297	0.282	0.281	0.288	0.280	0.280	0.279	0.286	0.279
0.4	3	80	0.397	0.272	0.271	0.278	0.271	0.271	0.270	0.277	0.270
0.5	3	80	0.496	0.253	0.252	0.258	0.251	0.252	0.251	0.258	0.251
0.6	3	80	0.596	0.223	0.223	0.228	0.223	0.224	0.223	0.228	0.222
0.7	3	80	0.696	0.184	0.183	0.188	0.184	0.185	0.184	0.189	0.184
0.8	3	80	0.797	0.133	0.133	0.136	0.134	0.135	0.134	0.138	0.134
0.9	3	80	0.898	0.072	0.072	0.074	0.073	0.074	0.073	0.075	0.073
0.1	4	80	0.099	0.209	0.208	0.211	0.210	0.208	0.207	0.211	0.207
0.2	4	80	0.198	0.228	0.227	0.231	0.228	0.227	0.226	0.230	0.226
0.3	4	80	0.297	0.237	0.236	0.240	0.236	0.236	0.235	0.239	0.235
0.4	4	80	0.397	0.236	0.234	0.239	0.234	0.235	0.234	0.238	0.233
0.5	4	80	0.496	0.224	0.223	0.227	0.222	0.223	0.222	0.226	0.222
0.6	4	80	0.596	0.201	0.200	0.204	0.200	0.201	0.200	0.204	0.200
0.7	4	80	0.696	0.168	0.167	0.170	0.167	0.168	0.168	0.171	0.168
0.8	4	80	0.797	0.123	0.123	0.125	0.123	0.124	0.124	0.126	0.124
0.9	4	80	0.898	0.068	0.067	0.069	0.068	0.069	0.068	0.070	0.068
0.1	5	80	0.100	0.174	0.173	0.176	0.176	0.174	0.173	0.176	0.174
0.2	5	80	0.199	0.199	0.198	0.201	0.199	0.198	0.197	0.200	0.197
0.3	5	80	0.298	0.213	0.212	0.215	0.212	0.212	0.211	0.214	0.211
0.4	5	80	0.397	0.216	0.215	0.218	0.215	0.215	0.214	0.217	0.214
0.5	5	80	0.496	0.208	0.207	0.210	0.206	0.208	0.206	0.210	0.206
0.6	5	80	0.596	0.189	0.188	0.191	0.188	0.189	0.188	0.191	0.188
0.7	5	80	0.696	0.159	0.158	0.161	0.158	0.160	0.159	0.161	0.159
0.8	5	80	0.797	0.118	0.117	0.119	0.118	0.119	0.118	0.120	0.118
0.9	5	80	0.898	0.065	0.065	0.066	0.065	0.066	0.066	0.067	0.066
0.1	6	80	0.099	0.152	0.152	0.154	0.154	0.152	0.151	0.153	0.152
0.2	6	80	0.199	0.180	0.180	0.182	0.181	0.180	0.179	0.181	0.179
0.3	6	80	0.298	0.197	0.196	0.199	0.197	0.197	0.196	0.198	0.196
0.4	6	80	0.397	0.203	0.202	0.205	0.202	0.203	0.202	0.204	0.201
0.5	6	80	0.496	0.198	0.197	0.200	0.197	0.198	0.197	0.199	0.196
0.6	6	80	0.596	0.182	0.181	0.183	0.180	0.182	0.181	0.183	0.180
0.7	6	80	0.696	0.154	0.153	0.155	0.153	0.155	0.154	0.156	0.154
0.8	6	80	0.797	0.115	0.114	0.116	0.114	0.116	0.115	0.117	0.115
0.9	6	80	0.898	0.064	0.063	0.064	0.064	0.064	0.064	0.065	0.064
0.1	7	80	0.099	0.138	0.137	0.138	0.140	0.137	0.137	0.138	0.137
0.2	7	80	0.199	0.168	0.167	0.169	0.169	0.167	0.167	0.169	0.167
0.3	7	80	0.298	0.187	0.186	0.188	0.187	0.186	0.185	0.188	0.185
0.4	7	80	0.397	0.195	0.194	0.196	0.194	0.194	0.193	0.195	0.193
0.5	7	80	0.496	0.191	0.190	0.193	0.190	0.191	0.190	0.192	0.190
0.6	7	80	0.596	0.177	0.176	0.178	0.175	0.177	0.176	0.178	0.175
0.7	7	80	0.696	0.150	0.150	0.151	0.149	0.151	0.150	0.152	0.150
0.8	7	80	0.797	0.113	0.112	0.113	0.112	0.113	0.113	0.114	0.113
0.9	7	80	0.898	0.063	0.062	0.063	0.062	0.063	0.063	0.064	0.063
0.1	8	80	0.099	0.127	0.126	0.127	0.129	0.126	0.126	0.127	0.127
0.2	8	80	0.199	0.159	0.158	0.159	0.160	0.158	0.157	0.159	0.158
0.3	8	80	0.298	0.179	0.178	0.180	0.180	0.179	0.178	0.180	0.178

0.4	8	80	0.397	0.188	0.187	0.189	0.188	0.188	0.187	0.189	0.187
0.5	8	80	0.496	0.186	0.185	0.187	0.185	0.186	0.185	0.187	0.185
0.6	8	80	0.596	0.173	0.172	0.174	0.172	0.173	0.172	0.174	0.172
0.7	8	80	0.696	0.148	0.147	0.149	0.147	0.148	0.147	0.149	0.147
0.8	8	80	0.797	0.111	0.110	0.112	0.110	0.112	0.111	0.112	0.111
0.9	8	80	0.898	0.062	0.061	0.062	0.062	0.063	0.062	0.063	0.062
0.1	9	80	0.099	0.118	0.118	0.119	0.121	0.118	0.117	0.119	0.118
0.2	9	80	0.199	0.152	0.151	0.152	0.153	0.151	0.150	0.152	0.151
0.3	9	80	0.298	0.173	0.172	0.174	0.174	0.173	0.172	0.174	0.172
0.4	9	80	0.397	0.184	0.183	0.185	0.183	0.183	0.182	0.184	0.182
0.5	9	80	0.496	0.183	0.182	0.183	0.182	0.182	0.181	0.183	0.181
0.6	9	80	0.596	0.170	0.169	0.171	0.169	0.170	0.169	0.171	0.169
0.7	9	80	0.696	0.146	0.145	0.147	0.145	0.146	0.145	0.147	0.145
0.8	9	80	0.797	0.110	0.109	0.110	0.109	0.110	0.110	0.111	0.110
0.9	9	80	0.898	0.061	0.061	0.062	0.061	0.062	0.062	0.062	0.062
0.1	10	80	0.100	0.112	0.111	0.112	0.114	0.112	0.111	0.112	0.112
0.2	10	80	0.199	0.146	0.145	0.147	0.148	0.146	0.145	0.146	0.145
0.3	10	80	0.298	0.169	0.168	0.169	0.169	0.168	0.167	0.169	0.168
0.4	10	80	0.397	0.180	0.179	0.181	0.180	0.180	0.179	0.180	0.178
0.5	10	80	0.497	0.180	0.179	0.180	0.179	0.179	0.178	0.180	0.178
0.6	10	80	0.596	0.168	0.167	0.169	0.167	0.168	0.167	0.169	0.167
0.7	10	80	0.696	0.144	0.143	0.145	0.143	0.145	0.144	0.145	0.144
0.8	10	80	0.797	0.109	0.108	0.109	0.108	0.109	0.109	0.110	0.109
0.9	10	80	0.898	0.061	0.060	0.061	0.061	0.061	0.061	0.062	0.061
0.1	2	90	0.099	0.406	0.405	0.422	0.403	0.400	0.399	0.416	0.400
0.2	2	90	0.198	0.394	0.393	0.410	0.391	0.389	0.388	0.404	0.389
0.3	2	90	0.297	0.374	0.373	0.389	0.372	0.370	0.369	0.385	0.370
0.4	2	90	0.397	0.346	0.345	0.360	0.345	0.343	0.342	0.357	0.343
0.5	2	90	0.496	0.310	0.309	0.322	0.310	0.309	0.308	0.321	0.309
0.6	2	90	0.596	0.265	0.264	0.276	0.267	0.265	0.265	0.276	0.265
0.7	2	90	0.696	0.212	0.212	0.221	0.215	0.213	0.213	0.222	0.213
0.8	2	90	0.797	0.151	0.150	0.157	0.153	0.152	0.152	0.159	0.152
0.9	2	90	0.898	0.080	0.080	0.083	0.082	0.082	0.081	0.085	0.082
0.1	3	90	0.100	0.256	0.255	0.261	0.256	0.255	0.254	0.260	0.254
0.2	3	90	0.199	0.266	0.265	0.271	0.265	0.264	0.263	0.269	0.263
0.3	3	90	0.298	0.266	0.265	0.271	0.264	0.265	0.264	0.270	0.263
0.4	3	90	0.397	0.257	0.256	0.262	0.255	0.256	0.255	0.261	0.255
0.5	3	90	0.497	0.238	0.238	0.243	0.237	0.238	0.237	0.242	0.237
0.6	3	90	0.596	0.210	0.210	0.214	0.210	0.211	0.210	0.215	0.210
0.7	3	90	0.697	0.173	0.172	0.176	0.173	0.174	0.173	0.177	0.173
0.8	3	90	0.797	0.126	0.125	0.128	0.126	0.127	0.126	0.129	0.126
0.9	3	90	0.898	0.068	0.068	0.069	0.069	0.069	0.069	0.070	0.069
0.1	4	90	0.100	0.197	0.196	0.199	0.198	0.196	0.195	0.199	0.196
0.2	4	90	0.199	0.215	0.214	0.218	0.215	0.214	0.213	0.217	0.213
0.3	4	90	0.298	0.224	0.223	0.226	0.223	0.223	0.222	0.225	0.222
0.4	4	90	0.397	0.222	0.221	0.225	0.221	0.221	0.221	0.224	0.220
0.5	4	90	0.497	0.211	0.210	0.213	0.209	0.210	0.210	0.213	0.209
0.6	4	90	0.597	0.189	0.189	0.192	0.188	0.190	0.189	0.192	0.188
0.7	4	90	0.697	0.158	0.157	0.160	0.157	0.159	0.158	0.160	0.158
0.8	4	90	0.797	0.116	0.116	0.118	0.116	0.117	0.116	0.118	0.117

0.9	4	90	0.898	0.064	0.063	0.064	0.064	0.064	0.064	0.065	0.064
0.1	5	90	0.100	0.164	0.164	0.166	0.166	0.164	0.163	0.165	0.164
0.2	5	90	0.199	0.188	0.187	0.189	0.188	0.187	0.186	0.189	0.186
0.3	5	90	0.298	0.201	0.200	0.202	0.200	0.200	0.199	0.202	0.199
0.4	5	90	0.398	0.203	0.202	0.205	0.202	0.203	0.202	0.205	0.202
0.5	5	90	0.497	0.196	0.195	0.198	0.195	0.196	0.195	0.197	0.194
0.6	5	90	0.597	0.178	0.177	0.180	0.177	0.178	0.177	0.180	0.177
0.7	5	90	0.697	0.150	0.149	0.151	0.149	0.150	0.150	0.152	0.150
0.8	5	90	0.797	0.111	0.111	0.112	0.111	0.112	0.111	0.113	0.111
0.9	5	90	0.898	0.061	0.061	0.062	0.061	0.062	0.062	0.063	0.062
0.1	6	90	0.100	0.144	0.143	0.145	0.146	0.144	0.143	0.145	0.144
0.2	6	90	0.199	0.170	0.169	0.171	0.171	0.170	0.169	0.171	0.169
0.3	6	90	0.298	0.186	0.185	0.187	0.186	0.186	0.185	0.187	0.185
0.4	6	90	0.398	0.192	0.191	0.193	0.191	0.191	0.190	0.193	0.190
0.5	6	90	0.497	0.187	0.186	0.188	0.186	0.186	0.186	0.188	0.185
0.6	6	90	0.597	0.171	0.170	0.172	0.170	0.171	0.170	0.172	0.170
0.7	6	90	0.697	0.145	0.144	0.146	0.144	0.145	0.145	0.146	0.145
0.8	6	90	0.797	0.108	0.107	0.109	0.108	0.109	0.108	0.109	0.108
0.9	6	90	0.898	0.060	0.060	0.060	0.060	0.060	0.060	0.061	0.060
0.1	7	90	0.100	0.130	0.129	0.131	0.132	0.130	0.129	0.130	0.130
0.2	7	90	0.199	0.158	0.158	0.159	0.159	0.158	0.157	0.159	0.158
0.3	7	90	0.298	0.176	0.175	0.177	0.177	0.176	0.175	0.177	0.175
0.4	7	90	0.398	0.184	0.183	0.185	0.183	0.183	0.182	0.184	0.182
0.5	7	90	0.497	0.180	0.179	0.181	0.179	0.180	0.179	0.181	0.179
0.6	7	90	0.597	0.166	0.166	0.167	0.165	0.166	0.166	0.167	0.165
0.7	7	90	0.697	0.142	0.141	0.142	0.141	0.142	0.141	0.143	0.141
0.8	7	90	0.797	0.106	0.105	0.106	0.105	0.107	0.106	0.107	0.106
0.9	7	90	0.898	0.059	0.059	0.059	0.059	0.059	0.059	0.060	0.059
0.1	8	90	0.100	0.119	0.119	0.120	0.121	0.119	0.119	0.120	0.119
0.2	8	90	0.199	0.150	0.149	0.150	0.151	0.149	0.149	0.150	0.149
0.3	8	90	0.298	0.169	0.168	0.170	0.169	0.169	0.168	0.170	0.168
0.4	8	90	0.398	0.178	0.177	0.179	0.177	0.177	0.176	0.178	0.176
0.5	8	90	0.497	0.176	0.175	0.177	0.175	0.175	0.175	0.176	0.174
0.6	8	90	0.597	0.163	0.162	0.164	0.162	0.163	0.162	0.164	0.162
0.7	8	90	0.697	0.139	0.138	0.140	0.138	0.140	0.139	0.140	0.139
0.8	8	90	0.797	0.104	0.104	0.105	0.104	0.105	0.104	0.105	0.105
0.9	8	90	0.898	0.058	0.058	0.058	0.058	0.059	0.058	0.059	0.059
0.1	9	90	0.100	0.112	0.111	0.112	0.113	0.111	0.111	0.112	0.112
0.2	9	90	0.199	0.143	0.142	0.144	0.144	0.143	0.142	0.143	0.142
0.3	9	90	0.298	0.164	0.163	0.164	0.164	0.163	0.162	0.164	0.162
0.4	9	90	0.398	0.173	0.172	0.174	0.173	0.173	0.172	0.174	0.172
0.5	9	90	0.497	0.172	0.171	0.173	0.171	0.172	0.171	0.173	0.171
0.6	9	90	0.597	0.160	0.159	0.161	0.159	0.160	0.159	0.161	0.159
0.7	9	90	0.697	0.137	0.137	0.138	0.136	0.138	0.137	0.138	0.137
0.8	9	90	0.797	0.103	0.103	0.104	0.103	0.104	0.103	0.104	0.103
0.9	9	90	0.898	0.058	0.057	0.058	0.057	0.058	0.058	0.058	0.058
0.1	10	90	0.100	0.105	0.105	0.106	0.107	0.105	0.105	0.106	0.105
0.2	10	90	0.199	0.138	0.137	0.138	0.139	0.138	0.137	0.138	0.137
0.3	10	90	0.298	0.159	0.158	0.160	0.160	0.159	0.158	0.159	0.158
0.4	10	90	0.398	0.170	0.169	0.170	0.170	0.169	0.169	0.170	0.168

0.5	10	90	0.497	0.169	0.169	0.170	0.169	0.169	0.168	0.170	0.168
0.6	10	90	0.597	0.158	0.157	0.159	0.157	0.158	0.157	0.159	0.157
0.7	10	90	0.697	0.136	0.135	0.136	0.135	0.136	0.135	0.137	0.135
0.8	10	90	0.797	0.102	0.102	0.103	0.102	0.103	0.102	0.103	0.102
0.9	10	90	0.898	0.057	0.057	0.057	0.057	0.058	0.057	0.058	0.058
0.1	2	100	0.098	0.385	0.384	0.399	0.383	0.381	0.380	0.394	0.381
0.2	2	100	0.198	0.374	0.373	0.388	0.372	0.370	0.369	0.383	0.370
0.3	2	100	0.297	0.355	0.354	0.368	0.354	0.352	0.351	0.364	0.352
0.4	2	100	0.396	0.328	0.328	0.340	0.328	0.326	0.325	0.338	0.326
0.5	2	100	0.496	0.294	0.293	0.305	0.295	0.293	0.292	0.304	0.293
0.6	2	100	0.596	0.252	0.251	0.261	0.253	0.252	0.251	0.261	0.252
0.7	2	100	0.696	0.201	0.201	0.209	0.204	0.202	0.202	0.210	0.202
0.8	2	100	0.797	0.143	0.142	0.148	0.145	0.144	0.144	0.150	0.144
0.9	2	100	0.898	0.076	0.076	0.078	0.078	0.077	0.077	0.080	0.077
0.1	3	100	0.100	0.243	0.243	0.248	0.243	0.242	0.241	0.246	0.241
0.2	3	100	0.199	0.252	0.251	0.257	0.251	0.251	0.250	0.255	0.250
0.3	3	100	0.298	0.252	0.252	0.257	0.251	0.251	0.250	0.256	0.250
0.4	3	100	0.398	0.244	0.243	0.248	0.242	0.243	0.242	0.247	0.242
0.5	3	100	0.497	0.226	0.225	0.230	0.225	0.226	0.225	0.230	0.225
0.6	3	100	0.597	0.200	0.199	0.203	0.199	0.200	0.199	0.203	0.199
0.7	3	100	0.697	0.164	0.163	0.167	0.164	0.165	0.164	0.167	0.164
0.8	3	100	0.797	0.119	0.119	0.121	0.119	0.120	0.119	0.122	0.120
0.9	3	100	0.898	0.064	0.064	0.066	0.065	0.065	0.065	0.066	0.065
0.1	4	100	0.100	0.187	0.186	0.189	0.187	0.186	0.186	0.188	0.186
0.2	4	100	0.199	0.204	0.203	0.206	0.204	0.203	0.203	0.206	0.203
0.3	4	100	0.298	0.212	0.211	0.214	0.211	0.211	0.211	0.214	0.210
0.4	4	100	0.398	0.211	0.210	0.213	0.210	0.210	0.209	0.212	0.209
0.5	4	100	0.497	0.200	0.199	0.202	0.199	0.200	0.199	0.202	0.199
0.6	4	100	0.597	0.180	0.179	0.182	0.179	0.180	0.179	0.182	0.179
0.7	4	100	0.697	0.150	0.149	0.151	0.149	0.150	0.150	0.152	0.150
0.8	4	100	0.797	0.110	0.110	0.111	0.110	0.111	0.110	0.112	0.110
0.9	4	100	0.898	0.060	0.060	0.061	0.060	0.061	0.061	0.062	0.061
0.1	5	100	0.100	0.156	0.155	0.157	0.157	0.156	0.155	0.157	0.155
0.2	5	100	0.199	0.178	0.177	0.179	0.178	0.178	0.177	0.179	0.177
0.3	5	100	0.298	0.190	0.190	0.192	0.190	0.190	0.189	0.191	0.189
0.4	5	100	0.398	0.193	0.192	0.195	0.192	0.193	0.192	0.194	0.191
0.5	5	100	0.497	0.186	0.185	0.187	0.185	0.186	0.185	0.187	0.185
0.6	5	100	0.597	0.169	0.168	0.170	0.168	0.169	0.168	0.170	0.168
0.7	5	100	0.697	0.142	0.142	0.143	0.142	0.143	0.142	0.144	0.142
0.8	5	100	0.797	0.105	0.105	0.106	0.105	0.106	0.106	0.107	0.106
0.9	5	100	0.898	0.058	0.058	0.058	0.058	0.059	0.058	0.059	0.059
0.1	6	100	0.100	0.136	0.136	0.137	0.138	0.136	0.136	0.137	0.136
0.2	6	100	0.199	0.162	0.161	0.163	0.162	0.161	0.161	0.162	0.161
0.3	6	100	0.298	0.177	0.176	0.178	0.177	0.176	0.176	0.177	0.175
0.4	6	100	0.398	0.182	0.181	0.183	0.181	0.181	0.181	0.183	0.180
0.5	6	100	0.497	0.177	0.176	0.178	0.176	0.177	0.176	0.178	0.176
0.6	6	100	0.597	0.162	0.162	0.163	0.161	0.162	0.162	0.163	0.162
0.7	6	100	0.697	0.138	0.137	0.138	0.137	0.138	0.137	0.139	0.137
0.8	6	100	0.797	0.102	0.102	0.103	0.102	0.103	0.103	0.104	0.103
0.9	6	100	0.898	0.057	0.056	0.057	0.057	0.057	0.057	0.058	0.057

0.1	7	100	0.100	0.123	0.123	0.124	0.125	0.123	0.122	0.124	0.123
0.2	7	100	0.199	0.150	0.150	0.151	0.151	0.150	0.149	0.151	0.150
0.3	7	100	0.298	0.167	0.167	0.168	0.167	0.167	0.166	0.168	0.166
0.4	7	100	0.398	0.174	0.173	0.175	0.174	0.174	0.173	0.175	0.173
0.5	7	100	0.497	0.171	0.170	0.172	0.170	0.171	0.170	0.172	0.170
0.6	7	100	0.597	0.158	0.157	0.159	0.157	0.158	0.157	0.159	0.157
0.7	7	100	0.697	0.134	0.134	0.135	0.134	0.135	0.134	0.135	0.134
0.8	7	100	0.797	0.100	0.100	0.101	0.100	0.101	0.100	0.101	0.101
0.9	7	100	0.898	0.056	0.055	0.056	0.056	0.056	0.056	0.057	0.056
0.1	8	100	0.100	0.113	0.113	0.114	0.115	0.113	0.113	0.114	0.113
0.2	8	100	0.199	0.142	0.141	0.143	0.143	0.142	0.141	0.142	0.141
0.3	8	100	0.298	0.160	0.160	0.161	0.161	0.160	0.159	0.161	0.159
0.4	8	100	0.398	0.169	0.168	0.169	0.168	0.168	0.168	0.169	0.167
0.5	8	100	0.497	0.167	0.166	0.167	0.166	0.166	0.166	0.167	0.165
0.6	8	100	0.597	0.154	0.154	0.155	0.154	0.155	0.154	0.155	0.154
0.7	8	100	0.697	0.132	0.131	0.133	0.131	0.132	0.132	0.133	0.132
0.8	8	100	0.797	0.099	0.098	0.099	0.098	0.099	0.099	0.100	0.099
0.9	8	100	0.898	0.055	0.055	0.055	0.055	0.056	0.055	0.056	0.055
0.1	9	100	0.100	0.106	0.105	0.106	0.107	0.106	0.105	0.106	0.106
0.2	9	100	0.199	0.136	0.135	0.136	0.137	0.135	0.135	0.136	0.135
0.3	9	100	0.298	0.155	0.154	0.156	0.156	0.155	0.154	0.155	0.154
0.4	9	100	0.398	0.164	0.164	0.165	0.164	0.164	0.163	0.165	0.163
0.5	9	100	0.497	0.163	0.163	0.164	0.163	0.163	0.162	0.164	0.162
0.6	9	100	0.597	0.152	0.151	0.153	0.151	0.152	0.151	0.153	0.151
0.7	9	100	0.697	0.130	0.130	0.131	0.129	0.130	0.130	0.131	0.130
0.8	9	100	0.797	0.098	0.097	0.098	0.097	0.098	0.098	0.099	0.098
0.9	9	100	0.898	0.055	0.054	0.055	0.054	0.055	0.055	0.055	0.055
0.1	10	100	0.100	0.100	0.099	0.100	0.102	0.100	0.099	0.100	0.100
0.2	10	100	0.199	0.131	0.130	0.131	0.132	0.130	0.130	0.131	0.130
0.3	10	100	0.298	0.151	0.150	0.152	0.152	0.151	0.150	0.151	0.150
0.4	10	100	0.398	0.161	0.160	0.162	0.161	0.161	0.160	0.161	0.160
0.5	10	100	0.497	0.161	0.160	0.161	0.160	0.161	0.160	0.161	0.160
0.6	10	100	0.597	0.150	0.149	0.150	0.149	0.150	0.149	0.151	0.149
0.7	10	100	0.697	0.129	0.128	0.129	0.128	0.129	0.128	0.130	0.128
0.8	10	100	0.797	0.097	0.096	0.097	0.096	0.097	0.097	0.098	0.097
0.9	10	100	0.898	0.054	0.054	0.054	0.054	0.055	0.054	0.055	0.054

0.3 Supplementary Table 3

This table shows the result of analysis of the previous two tables. The fourth to the twelfth column indicates when the 95% coverage probabilities of the eight confidence interval methods show acceptable coverage (i.e.,between 0.947 to 0.953) indicated by Y (acceptable) or N (not-acceptable). The last column of the table (W_{min}) shows the confidence interval method which has the minimum average width under the condition that the coverage probability of that confidence interval method is acceptable.

ρ	k	n	$Wald_S$	$Wald_F$	$Wald_{Ze}$	F	Z_S	Z_F	Z_{Ze}	ZF_ρ	W_{min}
0.1	2	20	N	N	N	Y	N	N	N	N	F

0.2	2	20	N	N	N	Y	N	N	N	N	F
0.3	2	20	N	N	N	Y	N	N	N	N	F
0.4	2	20	N	N	N	Y	Y	N	N	Y	Z_S,ZF_ρ
0.5	2	20	N	N	N	Y	Y	N	N	Y	Z_S,ZF_ρ
0.6	2	20	N	N	Y	Y	Y	N	N	Y	Z_S,ZF_ρ
0.7	2	20	N	N	Y	Y	Y	N	N	Y	Z_S,ZF_ρ
0.8	2	20	N	N	Y	Y	Y	N	N	Y	Z_S,ZF_ρ
0.9	2	20	N	N	Y	Y	Y	N	N	Y	Z_S,ZF_ρ
0.1	3	20	N	N	N	Y	N	N	Y	N	F
0.2	3	20	N	N	N	Y	N	N	Y	N	F
0.3	3	20	N	N	N	Y	N	N	Y	N	F
0.4	3	20	N	N	N	Y	N	N	Y	N	F
0.5	3	20	N	N	N	Y	N	N	N	N	F
0.6	3	20	N	N	Y	Y	N	N	N	N	F
0.7	3	20	N	N	Y	Y	N	N	N	N	F
0.8	3	20	N	N	Y	Y	N	N	N	N	F
0.9	3	20	N	N	Y	Y	N	N	N	N	F
0.1	4	20	N	N	N	Y	N	N	N	N	F
0.2	4	20	N	N	N	Y	N	N	N	N	F
0.3	4	20	N	N	N	Y	N	N	N	N	F
0.4	4	20	N	N	N	Y	N	N	N	N	F
0.5	4	20	N	N	N	Y	N	N	Y	N	F
0.6	4	20	N	N	Y	Y	N	N	Y	N	F
0.7	4	20	N	N	Y	Y	N	N	Y	N	F
0.8	4	20	N	N	Y	Y	N	N	Y	N	F
0.9	4	20	N	N	Y	Y	N	N	N	N	F
0.1	5	20	N	N	N	Y	N	N	N	N	F
0.2	5	20	N	N	N	Y	N	N	N	N	F
0.3	5	20	N	N	N	Y	N	N	N	N	F
0.4	5	20	N	N	N	Y	N	N	N	N	F
0.5	5	20	N	N	N	Y	N	N	N	N	F
0.6	5	20	N	N	N	Y	N	N	N	N	F
0.7	5	20	N	N	Y	Y	N	N	Y	N	F
0.8	5	20	N	N	Y	Y	N	N	Y	N	F
0.9	5	20	N	N	Y	Y	N	N	Y	N	F
0.1	6	20	N	N	N	Y	N	N	N	N	F
0.2	6	20	N	N	N	Y	N	N	N	N	F
0.3	6	20	N	N	N	Y	N	N	N	N	F
0.4	6	20	N	N	N	Y	N	N	N	Y	ZF_ρ
0.5	6	20	N	N	N	Y	N	N	N	Y	ZF_ρ
0.6	6	20	N	N	N	Y	N	N	N	N	F
0.7	6	20	N	N	N	Y	N	N	N	N	F
0.8	6	20	N	N	Y	Y	N	N	Y	N	F
0.9	6	20	N	N	Y	Y	N	N	Y	N	F
0.1	7	20	N	N	N	Y	N	N	N	N	F
0.2	7	20	N	N	N	Y	N	N	N	N	F
0.3	7	20	N	N	N	Y	N	N	N	N	F
0.4	7	20	N	N	N	Y	N	N	N	N	F
0.5	7	20	N	N	N	Y	N	N	N	N	F
0.6	7	20	N	N	N	Y	N	N	N	N	F

0.7	7	20	N	N	N	Y	N	N	N	N	F
0.8	7	20	N	N	Y	Y	N	N	N	N	F
0.9	7	20	N	N	Y	Y	N	N	Y	N	F
0.1	8	20	N	N	N	Y	N	N	N	N	F
0.2	8	20	N	N	N	Y	N	N	N	N	F
0.3	8	20	N	N	N	Y	N	N	N	N	F
0.4	8	20	N	N	N	Y	N	N	N	N	F
0.5	8	20	N	N	N	Y	N	N	N	N	F
0.6	8	20	N	N	N	Y	N	N	N	N	F
0.7	8	20	N	N	N	Y	N	N	N	N	F
0.8	8	20	N	N	N	Y	N	N	N	N	F
0.9	8	20	Y	N	Y	Y	N	N	Y	N	F
0.1	9	20	N	N	N	Y	N	N	N	N	F
0.2	9	20	N	N	N	Y	N	N	N	N	F
0.3	9	20	N	N	N	Y	N	N	N	N	F
0.4	9	20	N	N	N	Y	N	N	N	N	F
0.5	9	20	N	N	N	Y	N	N	N	N	F
0.6	9	20	N	N	N	Y	N	N	N	N	F
0.7	9	20	N	N	N	Y	N	N	N	N	F
0.8	9	20	N	N	N	Y	N	N	N	N	F
0.9	9	20	Y	N	Y	Y	N	N	N	N	F
0.1	10	20	N	N	N	Y	N	N	N	N	F
0.2	10	20	N	N	N	Y	N	N	N	N	F
0.3	10	20	N	N	N	Y	N	N	N	N	F
0.4	10	20	N	N	N	Y	N	N	N	N	F
0.5	10	20	N	N	N	Y	N	N	N	N	F
0.6	10	20	N	N	N	Y	N	N	N	N	F
0.7	10	20	N	N	N	Y	N	N	N	N	F
0.8	10	20	N	N	N	Y	N	N	N	N	F
0.9	10	20	Y	N	Y	Y	N	N	N	N	F
0.1	2	30	N	N	Y	Y	Y	N	N	Y	Z_S,ZF_ρ
0.2	2	30	N	N	Y	Y	N	N	N	N	F
0.3	2	30	N	N	Y	Y	N	N	N	N	F
0.4	2	30	N	N	Y	Y	Y	N	N	Y	Z_S,ZF_ρ
0.5	2	30	N	N	Y	Y	Y	N	N	Y	Z_S,ZF_ρ
0.6	2	30	N	N	Y	Y	Y	N	N	Y	Z_S,ZF_ρ
0.7	2	30	N	N	Y	Y	Y	N	N	Y	Z_S,ZF_ρ
0.8	2	30	N	N	Y	Y	Y	N	N	Y	Z_S,ZF_ρ
0.9	2	30	N	N	Y	Y	Y	N	N	Y	Z_S,ZF_ρ
0.1	3	30	N	N	N	Y	N	N	Y	Y	ZF_ρ
0.2	3	30	N	N	N	Y	N	N	Y	Y	ZF_ρ
0.3	3	30	N	N	Y	Y	N	N	Y	Y	ZF_ρ
0.4	3	30	N	N	Y	Y	N	N	Y	Y	ZF_ρ
0.5	3	30	N	N	Y	Y	N	N	N	Y	ZF_ρ
0.6	3	30	N	N	Y	Y	N	N	N	Y	ZF_ρ
0.7	3	30	N	N	Y	Y	N	N	N	Y	F
0.8	3	30	N	N	Y	Y	Y	N	N	Y	F
0.9	3	30	N	N	Y	Y	N	N	N	Y	F
0.1	4	30	N	N	N	Y	N	N	N	Y	ZF_ρ
0.2	4	30	N	N	N	Y	N	N	N	Y	ZF_ρ

0.3	4	30	N	N	N	Y	N	N	N	Y	ZF_ρ
0.4	4	30	N	N	N	Y	N	N	Y	Y	ZF_ρ
0.5	4	30	N	N	N	Y	N	N	Y	Y	ZF_ρ
0.6	4	30	N	N	Y	Y	N	N	Y	Y	F
0.7	4	30	N	N	Y	Y	N	N	Y	N	F
0.8	4	30	N	N	Y	Y	N	N	Y	N	F
0.9	4	30	N	N	Y	Y	N	N	Y	N	F
0.1	5	30	N	N	N	Y	N	N	N	Y	ZF_ρ
0.2	5	30	N	N	N	Y	N	N	N	Y	ZF_ρ
0.3	5	30	N	N	N	Y	N	N	N	Y	ZF_ρ
0.4	5	30	N	N	N	Y	N	N	N	Y	ZF_ρ
0.5	5	30	N	N	N	Y	N	N	N	Y	ZF_ρ
0.6	5	30	N	N	N	Y	N	N	N	N	F
0.7	5	30	N	N	Y	Y	N	N	Y	N	F
0.8	5	30	N	N	Y	Y	N	N	Y	N	F
0.9	5	30	Y	N	Y	Y	N	N	Y	N	$Wald_S$
0.1	6	30	N	N	N	Y	N	N	N	N	F
0.2	6	30	N	N	N	Y	N	N	N	Y	ZF_ρ
0.3	6	30	N	N	N	Y	N	N	N	Y	ZF_ρ
0.4	6	30	N	N	N	Y	N	N	N	N	F
0.5	6	30	N	N	N	Y	N	N	N	N	F
0.6	6	30	N	N	N	Y	N	N	N	N	F
0.7	6	30	N	N	N	Y	N	N	N	N	F
0.8	6	30	N	N	Y	Y	N	N	Y	N	F
0.9	6	30	Y	N	Y	Y	N	N	Y	N	F
0.1	7	30	N	N	N	Y	N	N	N	N	F
0.2	7	30	N	N	N	Y	N	N	N	N	F
0.3	7	30	N	N	N	Y	N	N	N	N	F
0.4	7	30	N	N	N	Y	N	N	N	N	F
0.5	7	30	N	N	N	Y	N	N	N	N	F
0.6	7	30	N	N	N	Y	N	N	N	N	F
0.7	7	30	N	N	N	Y	N	N	N	N	F
0.8	7	30	N	N	Y	Y	N	N	Y	N	F
0.9	7	30	Y	N	Y	Y	N	N	Y	N	F
0.1	8	30	N	N	N	Y	N	N	N	N	F
0.2	8	30	N	N	N	Y	N	N	N	N	F
0.3	8	30	N	N	N	Y	N	N	N	N	F
0.4	8	30	N	N	N	Y	N	N	N	N	F
0.5	8	30	N	N	N	Y	N	N	N	N	F
0.6	8	30	N	N	N	Y	N	N	N	N	F
0.7	8	30	N	N	N	Y	N	N	N	N	F
0.8	8	30	N	N	Y	Y	N	N	N	N	F
0.9	8	30	Y	N	Y	Y	N	N	Y	N	F
0.1	9	30	N	N	N	Y	N	N	N	N	F
0.2	9	30	N	N	N	Y	N	N	N	N	F
0.3	9	30	N	N	N	Y	N	N	N	N	F
0.4	9	30	N	N	N	Y	N	N	N	N	F
0.5	9	30	N	N	N	Y	N	N	N	N	F
0.6	9	30	N	N	N	Y	N	N	N	N	F
0.7	9	30	N	N	N	Y	N	N	N	N	F

0.8	9	30	N	N	Y	Y	N	N	N	N	F
0.9	9	30	Y	N	Y	Y	N	N	N	N	F
0.1	10	30	N	N	N	Y	N	N	N	N	F
0.2	10	30	N	N	N	Y	N	N	N	N	F
0.3	10	30	N	N	N	Y	N	N	N	N	F
0.4	10	30	N	N	N	Y	N	N	N	N	F
0.5	10	30	N	N	N	Y	N	N	N	N	F
0.6	10	30	N	N	N	Y	N	N	N	N	F
0.7	10	30	N	N	N	Y	N	N	N	N	F
0.8	10	30	N	N	Y	Y	N	N	N	N	F
0.9	10	30	Y	N	Y	Y	N	N	N	N	F
0.1	2	40	N	N	Y	Y	Y	Y	N	Y	Z_F
0.2	2	40	N	N	Y	Y	Y	Y	N	Y	Z_F
0.3	2	40	N	N	Y	Y	Y	Y	N	Y	Z_F
0.4	2	40	N	N	Y	Y	Y	Y	N	Y	Z_F
0.5	2	40	N	N	Y	Y	Y	Y	N	Y	Z_F
0.6	2	40	N	N	Y	Y	Y	Y	N	Y	Z_F
0.7	2	40	N	N	Y	Y	Y	Y	N	Y	Z_F
0.8	2	40	N	N	Y	Y	Y	Y	N	Y	Z_F
0.9	2	40	N	N	Y	Y	Y	Y	N	Y	Z_F
0.1	3	40	N	N	N	Y	N	N	Y	Y	ZF_ρ
0.2	3	40	N	N	Y	Y	N	N	Y	Y	ZF_ρ
0.3	3	40	N	N	Y	Y	N	N	Y	Y	ZF_ρ
0.4	3	40	N	N	Y	Y	N	N	Y	Y	ZF_ρ
0.5	3	40	N	N	Y	Y	N	N	Y	Y	ZF_ρ
0.6	3	40	N	N	Y	Y	N	N	N	Y	ZF_ρ
0.7	3	40	N	N	Y	Y	Y	N	N	Y	F
0.8	3	40	N	N	Y	Y	Y	N	N	Y	F
0.9	3	40	N	N	Y	Y	Y	N	N	Y	F
0.1	4	40	N	N	N	Y	N	N	N	Y	ZF_ρ
0.2	4	40	N	N	N	Y	N	N	N	Y	ZF_ρ
0.3	4	40	N	N	N	Y	N	N	N	Y	ZF_ρ
0.4	4	40	N	N	N	Y	N	N	N	N	F
0.5	4	40	N	N	Y	Y	N	N	Y	Y	ZF_ρ
0.6	4	40	N	N	Y	Y	N	N	Y	Y	F
0.7	4	40	N	N	Y	Y	N	N	Y	Y	F
0.8	4	40	N	N	Y	Y	Y	N	Y	Y	F
0.9	4	40	Y	N	Y	Y	Y	N	Y	Y	$Wald_S$
0.1	5	40	N	N	N	Y	N	N	N	N	F
0.2	5	40	N	N	N	Y	N	N	N	N	F
0.3	5	40	N	N	N	Y	N	N	N	Y	ZF_ρ
0.4	5	40	N	N	N	Y	N	N	N	Y	ZF_ρ
0.5	5	40	N	N	N	Y	N	N	Y	Y	ZF_ρ
0.6	5	40	N	N	Y	Y	N	N	Y	Y	F
0.7	5	40	N	N	Y	Y	N	N	Y	Y	F
0.8	5	40	Y	N	Y	Y	N	N	Y	Y	F
0.9	5	40	Y	Y	Y	Y	Y	N	Y	Y	$Wald_F$
0.1	6	40	N	N	N	Y	N	N	N	Y	ZF_ρ
0.2	6	40	N	N	N	Y	N	N	N	Y	ZF_ρ
0.3	6	40	N	N	N	Y	N	N	N	Y	ZF_ρ

0.4	6	40	N	N	N	Y	N	N	N	Y	ZF_ρ
0.5	6	40	N	N	N	Y	N	N	Y	Y	ZF_ρ
0.6	6	40	N	N	Y	Y	N	N	Y	Y	F
0.7	6	40	N	N	Y	Y	N	N	Y	Y	F
0.8	6	40	Y	N	Y	Y	Y	N	Y	Y	F
0.9	6	40	Y	Y	Y	Y	Y	N	Y	Y	$Wald_F$
0.1	7	40	N	N	N	Y	N	N	N	Y	ZF_ρ
0.2	7	40	N	N	N	Y	N	N	N	Y	ZF_ρ
0.3	7	40	N	N	N	Y	N	N	N	Y	ZF_ρ
0.4	7	40	N	N	N	Y	N	N	N	Y	ZF_ρ
0.5	7	40	N	N	N	Y	N	N	N	Y	ZF_ρ
0.6	7	40	N	N	N	Y	N	N	N	Y	F
0.7	7	40	N	N	Y	Y	N	N	Y	Y	F
0.8	7	40	Y	N	Y	Y	N	N	Y	Y	F
0.9	7	40	Y	Y	Y	Y	Y	N	Y	Y	$Wald_F$
0.1	8	40	N	N	N	Y	N	N	N	N	F
0.2	8	40	N	N	N	Y	N	N	N	N	F
0.3	8	40	N	N	N	Y	N	N	N	N	F
0.4	8	40	N	N	N	Y	N	N	N	Y	ZF_ρ
0.5	8	40	N	N	N	Y	N	N	N	Y	ZF_ρ
0.6	8	40	N	N	N	Y	N	N	N	Y	F
0.7	8	40	N	N	Y	Y	N	N	N	Y	F
0.8	8	40	Y	Y	Y	Y	N	N	Y	Y	F
0.9	8	40	Y	Y	Y	Y	N	N	Y	Y	$Wald_F$
0.1	9	40	N	N	N	Y	N	N	N	N	F
0.2	9	40	N	N	N	Y	N	N	N	Y	ZF_ρ
0.3	9	40	N	N	N	Y	N	N	N	Y	ZF_ρ
0.4	9	40	N	N	N	Y	N	N	N	Y	ZF_ρ
0.5	9	40	N	N	N	Y	N	N	N	Y	ZF_ρ
0.6	9	40	N	N	N	Y	N	N	N	Y	F
0.7	9	40	Y	N	Y	Y	N	N	N	Y	F
0.8	9	40	Y	Y	Y	Y	N	N	Y	Y	F
0.9	9	40	Y	Y	Y	Y	N	N	Y	Y	$Wald_F$
0.1	10	40	N	N	N	Y	N	N	N	Y	ZF_ρ
0.2	10	40	N	N	N	Y	N	N	N	Y	ZF_ρ
0.3	10	40	N	N	N	Y	N	N	N	Y	ZF_ρ
0.4	10	40	N	N	N	Y	N	N	N	Y	ZF_ρ
0.5	10	40	N	N	N	Y	N	N	N	Y	ZF_ρ
0.6	10	40	N	N	N	Y	N	N	N	Y	ZF_ρ
0.7	10	40	Y	N	Y	Y	N	N	N	Y	F
0.8	10	40	Y	Y	Y	Y	N	N	Y	Y	F
0.9	10	40	Y	Y	Y	Y	N	N	Y	Y	$Wald_F$
0.1	2	50	N	N	Y	Y	Y	Y	N	Y	Z_F
0.2	2	50	N	N	Y	Y	Y	Y	N	Y	Z_F
0.3	2	50	N	N	Y	Y	Y	N	N	Y	Z_S,ZF_ρ
0.4	2	50	N	N	Y	Y	Y	Y	N	Y	Z_F
0.5	2	50	N	N	Y	Y	Y	Y	N	Y	Z_F
0.6	2	50	N	N	Y	Y	Y	Y	N	Y	Z_F
0.7	2	50	N	N	Y	Y	Y	Y	N	Y	Z_F
0.8	2	50	N	N	Y	Y	Y	Y	N	Y	Z_F

0.9	2	50	N	N	Y	Y	Y	Y	N	Y	Z_F
0.1	3	50	N	N	Y	Y	N	N	Y	Y	ZF_ρ
0.2	3	50	N	N	Y	Y	N	N	Y	Y	ZF_ρ
0.3	3	50	N	N	Y	Y	N	N	Y	Y	ZF_ρ
0.4	3	50	N	N	Y	Y	N	N	Y	Y	ZF_ρ
0.5	3	50	N	N	Y	Y	N	N	Y	Y	ZF_ρ
0.6	3	50	N	N	Y	Y	Y	N	Y	Y	ZF_ρ
0.7	3	50	N	N	Y	Y	Y	N	Y	Y	F
0.8	3	50	N	N	Y	Y	Y	Y	N	Y	F
0.9	3	50	Y	N	Y	Y	Y	Y	N	Y	$Wald_S$
0.1	4	50	N	N	N	Y	N	N	Y	Y	ZF_ρ
0.2	4	50	N	N	N	Y	N	N	Y	Y	ZF_ρ
0.3	4	50	N	N	N	Y	N	N	Y	Y	ZF_ρ
0.4	4	50	N	N	Y	Y	N	N	Y	Y	ZF_ρ
0.5	4	50	N	N	Y	Y	N	N	Y	Y	ZF_ρ
0.6	4	50	N	N	Y	Y	N	N	Y	Y	F
0.7	4	50	N	N	Y	Y	N	N	Y	Y	F
0.8	4	50	N	N	Y	Y	Y	N	Y	Y	F
0.9	4	50	Y	Y	Y	Y	Y	N	Y	Y	$Wald_F$
0.1	5	50	N	N	N	Y	N	N	N	Y	ZF_ρ
0.2	5	50	N	N	N	Y	N	N	N	Y	ZF_ρ
0.3	5	50	N	N	N	Y	N	N	N	Y	ZF_ρ
0.4	5	50	N	N	N	Y	N	N	Y	Y	ZF_ρ
0.5	5	50	N	N	N	Y	N	N	Y	Y	ZF_ρ
0.6	5	50	N	N	Y	Y	N	N	Y	Y	F
0.7	5	50	N	N	Y	Y	N	N	Y	Y	F
0.8	5	50	Y	N	Y	Y	N	N	Y	Y	F
0.9	5	50	Y	Y	Y	Y	Y	N	Y	Y	$Wald_F$
0.1	6	50	N	N	N	Y	N	N	N	Y	ZF_ρ
0.2	6	50	N	N	N	Y	N	N	N	Y	ZF_ρ
0.3	6	50	N	N	N	Y	N	N	N	Y	ZF_ρ
0.4	6	50	N	N	N	Y	N	N	N	Y	ZF_ρ
0.5	6	50	N	N	N	Y	N	N	N	Y	ZF_ρ
0.6	6	50	N	N	N	Y	N	N	Y	Y	F
0.7	6	50	N	N	N	Y	N	N	Y	Y	F
0.8	6	50	N	N	Y	Y	N	N	Y	Y	F
0.9	6	50	Y	Y	Y	Y	Y	N	Y	Y	$Wald_F$
0.1	7	50	N	N	N	Y	N	N	N	N	F
0.2	7	50	N	N	N	Y	N	N	N	N	F
0.3	7	50	N	N	N	Y	N	N	N	Y	ZF_ρ
0.4	7	50	N	N	N	Y	N	N	N	Y	ZF_ρ
0.5	7	50	N	N	N	Y	N	N	N	N	F
0.6	7	50	N	N	N	Y	N	N	N	Y	F
0.7	7	50	N	N	Y	Y	N	N	Y	Y	F
0.8	7	50	N	N	Y	Y	N	N	Y	Y	F
0.9	7	50	Y	Y	Y	Y	N	N	Y	Y	$Wald_F$
0.1	8	50	N	N	N	Y	N	N	N	Y	ZF_ρ
0.2	8	50	N	N	N	Y	N	N	N	Y	ZF_ρ
0.3	8	50	N	N	N	Y	N	N	N	Y	ZF_ρ
0.4	8	50	N	N	N	Y	N	N	N	Y	ZF_ρ

0.5	8	50	N	N	N	Y	N	N	N	Y	ZF_ρ
0.6	8	50	N	N	N	Y	N	N	N	Y	F
0.7	8	50	Y	N	Y	Y	N	N	Y	Y	F
0.8	8	50	Y	N	Y	Y	N	N	Y	Y	F
0.9	8	50	Y	Y	Y	Y	Y	N	Y	Y	$Wald_F$
0.1	9	50	N	N	N	Y	N	N	N	Y	ZF_ρ
0.2	9	50	N	N	N	Y	N	N	N	Y	ZF_ρ
0.3	9	50	N	N	N	Y	N	N	N	Y	ZF_ρ
0.4	9	50	N	N	N	Y	N	N	N	Y	ZF_ρ
0.5	9	50	N	N	N	Y	N	N	N	N	F
0.6	9	50	N	N	N	Y	N	N	N	Y	F
0.7	9	50	Y	N	Y	Y	N	N	N	Y	F
0.8	9	50	Y	N	Y	Y	N	N	Y	Y	F
0.9	9	50	Y	Y	Y	Y	Y	N	Y	Y	$Wald_F$
0.1	10	50	N	N	N	Y	N	N	N	Y	ZF_ρ
0.2	10	50	N	N	N	Y	N	N	N	Y	ZF_ρ
0.3	10	50	N	N	N	Y	N	N	N	Y	ZF_ρ
0.4	10	50	N	N	N	Y	N	N	N	Y	ZF_ρ
0.5	10	50	N	N	N	Y	N	N	N	Y	ZF_ρ
0.6	10	50	N	N	N	Y	N	N	N	Y	F
0.7	10	50	N	N	Y	Y	N	N	N	Y	F
0.8	10	50	Y	N	Y	Y	N	N	Y	Y	F
0.9	10	50	Y	Y	Y	Y	Y	N	Y	Y	$Wald_F$
0.1	2	60	N	N	Y	Y	Y	Y	N	Y	Z_F
0.2	2	60	N	N	Y	Y	Y	Y	N	Y	Z_F
0.3	2	60	N	N	Y	Y	Y	Y	N	Y	Z_F
0.4	2	60	N	N	Y	Y	Y	Y	N	Y	Z_F
0.5	2	60	N	N	Y	Y	Y	Y	N	Y	Z_F
0.6	2	60	N	N	Y	Y	Y	Y	N	Y	Z_F
0.7	2	60	N	N	Y	Y	Y	Y	N	Y	Z_F
0.8	2	60	N	N	Y	Y	Y	Y	N	Y	Z_F
0.9	2	60	N	N	Y	Y	Y	Y	N	Y	Z_F
0.1	3	60	N	N	Y	Y	N	N	Y	Y	ZF_ρ
0.2	3	60	N	N	Y	Y	N	N	Y	Y	ZF_ρ
0.3	3	60	N	N	Y	Y	N	N	Y	Y	ZF_ρ
0.4	3	60	N	N	Y	Y	N	N	Y	Y	ZF_ρ
0.5	3	60	N	N	Y	Y	N	N	Y	Y	ZF_ρ
0.6	3	60	N	N	Y	Y	Y	Y	Y	Y	ZF_ρ
0.7	3	60	N	N	Y	Y	Y	Y	Y	Y	F
0.8	3	60	N	N	Y	Y	Y	Y	Y	Y	F
0.9	3	60	N	N	Y	Y	Y	Y	Y	Y	F
0.1	4	60	N	N	N	Y	N	N	N	Y	ZF_ρ
0.2	4	60	N	N	N	Y	N	N	N	Y	ZF_ρ
0.3	4	60	N	N	N	Y	N	N	Y	Y	ZF_ρ
0.4	4	60	N	N	N	Y	N	N	Y	Y	ZF_ρ
0.5	4	60	N	N	Y	Y	N	N	Y	Y	ZF_ρ
0.6	4	60	N	N	Y	Y	N	N	Y	Y	F
0.7	4	60	N	N	Y	Y	Y	N	Y	Y	F
0.8	4	60	Y	N	Y	Y	Y	N	Y	Y	F
0.9	4	60	Y	Y	Y	Y	Y	N	Y	Y	$Wald_F$

0.1	5	60	N	N	N	Y	N	N	N	N	F
0.2	5	60	N	N	N	Y	N	N	N	N	F
0.3	5	60	N	N	N	Y	N	N	N	N	F
0.4	5	60	N	N	N	Y	N	N	N	Y	ZF_ρ
0.5	5	60	N	N	N	Y	N	N	Y	Y	ZF_ρ
0.6	5	60	N	N	N	Y	N	N	Y	Y	F
0.7	5	60	N	N	Y	Y	N	N	Y	Y	F
0.8	5	60	N	N	Y	Y	N	N	Y	Y	F
0.9	5	60	Y	N	Y	Y	N	N	Y	Y	$Wald_S$
0.1	6	60	N	N	N	Y	N	N	N	Y	ZF_ρ
0.2	6	60	N	N	N	Y	N	N	N	Y	ZF_ρ
0.3	6	60	N	N	N	Y	N	N	N	Y	ZF_ρ
0.4	6	60	N	N	N	Y	N	N	N	Y	ZF_ρ
0.5	6	60	N	N	N	Y	N	N	Y	Y	ZF_ρ
0.6	6	60	N	N	N	Y	N	N	Y	Y	F
0.7	6	60	N	N	Y	Y	N	N	Y	Y	F
0.8	6	60	Y	N	Y	Y	N	N	Y	Y	F
0.9	6	60	Y	N	Y	Y	Y	Y	Y	Y	F
0.1	7	60	N	N	N	Y	N	N	N	Y	ZF_ρ
0.2	7	60	N	N	N	Y	N	N	N	N	F
0.3	7	60	N	N	N	Y	N	N	N	N	F
0.4	7	60	N	N	N	Y	N	N	N	N	F
0.5	7	60	N	N	N	Y	N	N	N	N	F
0.6	7	60	N	N	N	Y	N	N	N	N	F
0.7	7	60	N	N	Y	Y	N	N	Y	N	F
0.8	7	60	N	N	Y	Y	N	N	Y	N	F
0.9	7	60	Y	N	Y	Y	Y	N	Y	Y	F
0.1	8	60	N	N	N	Y	N	N	N	N	F
0.2	8	60	N	N	N	Y	N	N	N	N	F
0.3	8	60	N	N	N	Y	N	N	N	N	F
0.4	8	60	N	N	N	Y	N	N	N	Y	ZF_ρ
0.5	8	60	N	N	N	Y	N	N	N	Y	ZF_ρ
0.6	8	60	N	N	N	Y	N	N	N	Y	F
0.7	8	60	N	N	N	Y	N	N	N	Y	F
0.8	8	60	N	N	Y	Y	N	N	Y	Y	F
0.9	8	60	Y	Y	Y	Y	N	N	Y	Y	$Wald_F$
0.1	9	60	N	N	N	Y	N	N	N	Y	ZF_ρ
0.2	9	60	N	N	N	Y	N	N	N	N	F
0.3	9	60	N	N	N	Y	N	N	N	N	F
0.4	9	60	N	N	N	Y	N	N	N	N	F
0.5	9	60	N	N	N	Y	N	N	N	N	F
0.6	9	60	N	N	N	Y	N	N	N	N	F
0.7	9	60	N	N	N	Y	N	N	N	N	F
0.8	9	60	N	N	Y	Y	N	N	Y	Y	F
0.9	9	60	Y	N	Y	Y	N	N	Y	Y	F
0.1	10	60	N	N	N	Y	N	N	N	Y	ZF_ρ
0.2	10	60	N	N	N	Y	N	N	N	Y	ZF_ρ
0.3	10	60	N	N	N	Y	N	N	N	N	F
0.4	10	60	N	N	N	Y	N	N	N	Y	ZF_ρ
0.5	10	60	N	N	N	Y	N	N	N	Y	ZF_ρ

0.6	10	60	N	N	N	Y	N	N	N	Y	F
0.7	10	60	N	N	Y	Y	N	N	N	Y	F
0.8	10	60	N	N	Y	Y	N	N	Y	Y	F
0.9	10	60	Y	N	Y	Y	N	N	Y	Y	F
0.1	2	70	N	N	Y	Y	Y	Y	N	Y	Z_F
0.2	2	70	N	N	Y	Y	Y	Y	N	Y	Z_F
0.3	2	70	N	N	Y	Y	Y	Y	N	Y	Z_F
0.4	2	70	N	N	Y	Y	Y	Y	N	Y	Z_F
0.5	2	70	N	N	Y	Y	Y	Y	N	Y	Z_F
0.6	2	70	N	N	Y	Y	Y	Y	N	Y	Z_F
0.7	2	70	N	N	Y	Y	Y	Y	N	Y	Z_F
0.8	2	70	N	N	Y	Y	Y	Y	N	Y	Z_F
0.9	2	70	N	N	Y	Y	Y	Y	N	Y	Z_F
0.1	3	70	N	N	Y	Y	Y	N	Y	Y	ZF_ρ
0.2	3	70	N	N	Y	Y	Y	Y	Y	Y	ZF_ρ
0.3	3	70	N	N	Y	Y	Y	Y	Y	Y	ZF_ρ
0.4	3	70	N	N	Y	Y	Y	N	Y	Y	ZF_ρ
0.5	3	70	N	N	Y	Y	Y	Y	Y	Y	ZF_ρ
0.6	3	70	N	N	Y	Y	Y	Y	Y	Y	ZF_ρ
0.7	3	70	N	N	Y	Y	Y	Y	Y	Y	F
0.8	3	70	Y	N	Y	Y	Y	Y	Y	Y	$Wald_S$
0.9	3	70	Y	N	Y	Y	Y	Y	Y	Y	$Wald_S$
0.1	4	70	N	N	N	Y	N	N	N	Y	ZF_ρ
0.2	4	70	N	N	Y	Y	N	N	Y	Y	ZF_ρ
0.3	4	70	N	N	Y	Y	N	N	Y	Y	ZF_ρ
0.4	4	70	N	N	Y	Y	N	N	Y	Y	ZF_ρ
0.5	4	70	N	N	Y	Y	N	N	Y	Y	ZF_ρ
0.6	4	70	N	N	Y	Y	Y	N	Y	Y	F
0.7	4	70	N	N	Y	Y	Y	N	Y	Y	F
0.8	4	70	Y	N	Y	Y	Y	Y	Y	Y	F
0.9	4	70	Y	Y	Y	Y	Y	N	Y	Y	$Wald_F$
0.1	5	70	N	N	N	Y	N	N	N	Y	ZF_ρ
0.2	5	70	N	N	N	Y	N	N	N	N	F
0.3	5	70	N	N	N	Y	N	N	N	Y	ZF_ρ
0.4	5	70	N	N	N	Y	N	N	Y	Y	ZF_ρ
0.5	5	70	N	N	N	Y	N	N	Y	Y	ZF_ρ
0.6	5	70	N	N	Y	Y	N	N	Y	Y	F
0.7	5	70	Y	N	Y	Y	N	N	Y	Y	F
0.8	5	70	Y	Y	Y	Y	Y	N	Y	Y	$Wald_F$
0.9	5	70	Y	Y	Y	Y	Y	N	Y	Y	$Wald_F$
0.1	6	70	N	N	N	Y	N	N	N	Y	ZF_ρ
0.2	6	70	N	N	N	Y	N	N	N	Y	ZF_ρ
0.3	6	70	N	N	N	Y	N	N	N	Y	ZF_ρ
0.4	6	70	N	N	N	Y	N	N	Y	Y	ZF_ρ
0.5	6	70	N	N	N	Y	N	N	Y	Y	ZF_ρ
0.6	6	70	N	N	Y	Y	N	N	Y	Y	F
0.7	6	70	N	N	Y	Y	N	N	Y	Y	F
0.8	6	70	Y	N	Y	Y	Y	N	Y	Y	F
0.9	6	70	Y	Y	Y	Y	Y	N	Y	Y	$Wald_F$
0.1	7	70	N	N	N	Y	N	N	N	Y	ZF_ρ

0.2	7	70	N	N	N	Y	N	N	N	Y	ZF_ρ
0.3	7	70	N	N	N	Y	N	N	N	Y	ZF_ρ
0.4	7	70	N	N	N	Y	N	N	N	Y	ZF_ρ
0.5	7	70	N	N	N	Y	N	N	N	Y	ZF_ρ
0.6	7	70	N	N	N	Y	N	N	Y	Y	F
0.7	7	70	N	N	Y	Y	N	N	Y	Y	F
0.8	7	70	Y	N	Y	Y	Y	N	Y	Y	F
0.9	7	70	Y	Y	Y	Y	Y	N	Y	Y	$Wald_F$
0.1	8	70	N	N	N	Y	N	N	N	Y	ZF_ρ
0.2	8	70	N	N	N	Y	N	N	N	Y	ZF_ρ
0.3	8	70	N	N	N	Y	N	N	N	Y	ZF_ρ
0.4	8	70	N	N	N	Y	N	N	N	Y	ZF_ρ
0.5	8	70	N	N	N	Y	N	N	N	Y	ZF_ρ
0.6	8	70	N	N	Y	Y	N	N	N	Y	F
0.7	8	70	N	N	Y	Y	N	N	Y	Y	F
0.8	8	70	Y	Y	Y	Y	N	N	Y	Y	F
0.9	8	70	Y	Y	Y	Y	Y	N	Y	Y	$Wald_F$
0.1	9	70	N	N	N	Y	N	N	N	Y	ZF_ρ
0.2	9	70	N	N	N	Y	N	N	N	Y	ZF_ρ
0.3	9	70	N	N	N	Y	N	N	N	Y	ZF_ρ
0.4	9	70	N	N	N	Y	N	N	N	Y	ZF_ρ
0.5	9	70	N	N	N	Y	N	N	N	Y	ZF_ρ
0.6	9	70	N	N	Y	Y	N	N	N	Y	F
0.7	9	70	Y	N	Y	Y	N	N	Y	Y	F
0.8	9	70	Y	N	Y	Y	Y	N	Y	Y	F
0.9	9	70	Y	N	Y	Y	Y	N	Y	Y	F
0.1	10	70	N	N	N	Y	N	N	N	Y	ZF_ρ
0.2	10	70	N	N	N	Y	N	N	N	Y	ZF_ρ
0.3	10	70	N	N	N	Y	N	N	N	Y	ZF_ρ
0.4	10	70	N	N	N	Y	N	N	N	Y	ZF_ρ
0.5	10	70	N	N	Y	Y	N	N	N	Y	ZF_ρ
0.6	10	70	N	N	N	Y	N	N	N	Y	F
0.7	10	70	N	N	N	Y	N	N	Y	Y	F
0.8	10	70	N	N	Y	Y	Y	N	Y	Y	F
0.9	10	70	Y	Y	Y	Y	Y	N	Y	Y	$Wald_F$
0.1	2	80	N	N	Y	Y	Y	Y	N	Y	Z_F
0.2	2	80	N	N	Y	Y	Y	Y	N	Y	Z_F
0.3	2	80	N	N	Y	Y	Y	Y	N	Y	Z_F
0.4	2	80	N	N	Y	Y	Y	Y	N	Y	Z_F
0.5	2	80	N	N	Y	Y	Y	Y	N	Y	Z_F
0.6	2	80	N	N	Y	Y	Y	Y	N	Y	Z_F
0.7	2	80	N	N	Y	Y	Y	Y	N	Y	Z_F
0.8	2	80	N	N	Y	Y	Y	Y	N	Y	Z_F
0.9	2	80	N	N	Y	Y	Y	Y	N	Y	Z_F
0.1	3	80	N	N	Y	Y	Y	Y	Y	Y	ZF_ρ
0.2	3	80	N	N	Y	Y	Y	Y	Y	Y	ZF_ρ
0.3	3	80	Y	N	Y	Y	Y	Y	Y	Y	ZF_ρ
0.4	3	80	N	N	Y	Y	Y	Y	Y	Y	ZF_ρ
0.5	3	80	N	N	Y	Y	Y	Y	Y	Y	ZF_ρ
0.6	3	80	Y	Y	Y	Y	Y	Y	Y	Y	ZF_ρ

0.7	3	80	Y	Y	Y	Y	Y	Y	Y	Y	$Wald_F$
0.8	3	80	Y	Y	Y	Y	Y	Y	Y	Y	$Wald_F$
0.9	3	80	Y	Y	Y	Y	Y	Y	Y	Y	$Wald_F$
0.1	4	80	N	N	Y	Y	Y	N	Y	Y	ZF_ρ
0.2	4	80	N	N	Y	Y	N	N	Y	Y	ZF_ρ
0.3	4	80	N	N	Y	Y	N	N	Y	Y	ZF_ρ
0.4	4	80	N	N	Y	Y	Y	N	Y	Y	ZF_ρ
0.5	4	80	N	N	Y	Y	Y	N	Y	Y	ZF_ρ
0.6	4	80	N	N	Y	Y	Y	N	Y	Y	F
0.7	4	80	Y	N	Y	Y	N	N	Y	Y	F
0.8	4	80	Y	N	Y	Y	Y	N	Y	Y	F
0.9	4	80	Y	Y	Y	Y	Y	Y	Y	Y	$Wald_F$
0.1	5	80	N	N	Y	Y	N	N	Y	Y	ZF_ρ
0.2	5	80	N	N	Y	Y	Y	N	Y	Y	ZF_ρ
0.3	5	80	N	N	Y	Y	Y	N	Y	Y	ZF_ρ
0.4	5	80	N	N	Y	Y	Y	N	Y	Y	ZF_ρ
0.5	5	80	N	N	Y	Y	Y	N	Y	Y	ZF_ρ
0.6	5	80	Y	N	Y	Y	Y	N	Y	Y	F
0.7	5	80	Y	N	Y	Y	Y	N	Y	Y	F
0.8	5	80	Y	Y	Y	Y	Y	Y	Y	Y	$Wald_F$
0.9	5	80	Y	Y	Y	Y	Y	Y	Y	Y	$Wald_F$
0.1	6	80	N	N	Y	Y	N	N	Y	Y	ZF_ρ
0.2	6	80	N	N	Y	Y	Y	N	Y	Y	ZF_ρ
0.3	6	80	N	N	Y	Y	Y	N	Y	Y	ZF_ρ
0.4	6	80	Y	N	Y	Y	Y	N	Y	Y	ZF_ρ
0.5	6	80	N	N	Y	Y	Y	Y	Y	Y	ZF_ρ
0.6	6	80	N	N	Y	Y	Y	N	Y	Y	F
0.7	6	80	Y	N	Y	Y	Y	N	Y	Y	F
0.8	6	80	Y	Y	Y	Y	Y	N	Y	Y	$Wald_F$
0.9	6	80	Y	Y	Y	Y	Y	Y	Y	Y	$Wald_F$
0.1	7	80	N	N	N	Y	N	N	N	Y	ZF_ρ
0.2	7	80	N	N	Y	Y	N	N	Y	Y	ZF_ρ
0.3	7	80	N	N	N	Y	N	N	N	Y	ZF_ρ
0.4	7	80	N	N	N	Y	N	N	N	Y	ZF_ρ
0.5	7	80	N	N	Y	Y	N	N	Y	Y	ZF_ρ
0.6	7	80	Y	N	Y	Y	N	N	Y	Y	F
0.7	7	80	Y	N	Y	Y	Y	N	Y	Y	F
0.8	7	80	Y	N	Y	Y	Y	Y	Y	Y	F
0.9	7	80	Y	Y	Y	Y	Y	Y	Y	Y	$Wald_F$
0.1	8	80	N	N	N	Y	N	N	N	Y	ZF_ρ
0.2	8	80	N	N	N	Y	N	N	N	Y	ZF_ρ
0.3	8	80	N	N	N	Y	N	N	N	Y	ZF_ρ
0.4	8	80	N	N	N	Y	N	N	N	Y	ZF_ρ
0.5	8	80	N	N	Y	Y	N	N	Y	Y	ZF_ρ
0.6	8	80	N	N	Y	Y	Y	N	Y	Y	F
0.7	8	80	N	N	Y	Y	Y	N	Y	Y	F
0.8	8	80	Y	N	Y	Y	Y	N	Y	Y	F
0.9	8	80	Y	Y	Y	Y	Y	N	Y	Y	$Wald_F$
0.1	9	80	N	N	N	Y	N	N	N	Y	ZF_ρ
0.2	9	80	N	N	N	Y	N	N	N	Y	ZF_ρ

0.3	9	80	N	N	N	Y	N	N	N	Y	ZF_ρ
0.4	9	80	N	N	N	Y	N	N	N	Y	ZF_ρ
0.5	9	80	N	N	N	Y	N	N	N	Y	ZF_ρ
0.6	9	80	N	N	Y	Y	N	N	Y	Y	F
0.7	9	80	N	N	Y	Y	N	N	Y	Y	F
0.8	9	80	Y	N	Y	Y	N	N	Y	Y	F
0.9	9	80	Y	Y	Y	Y	Y	N	Y	Y	$Wald_F$
0.1	10	80	N	N	N	Y	N	N	N	Y	ZF_ρ
0.2	10	80	N	N	N	Y	N	N	N	Y	ZF_ρ
0.3	10	80	N	N	N	Y	N	N	N	Y	ZF_ρ
0.4	10	80	N	N	N	Y	N	N	N	Y	ZF_ρ
0.5	10	80	N	N	N	Y	N	N	N	Y	ZF_ρ
0.6	10	80	N	N	N	Y	N	N	N	Y	F
0.7	10	80	N	N	Y	Y	N	N	Y	Y	F
0.8	10	80	Y	N	Y	Y	Y	N	Y	Y	F
0.9	10	80	Y	Y	Y	Y	Y	N	Y	Y	$Wald_F$
0.1	2	90	N	N	Y	Y	Y	Y	N	Y	Z_F
0.2	2	90	N	N	Y	Y	Y	Y	N	Y	Z_F
0.3	2	90	N	N	Y	Y	Y	Y	N	Y	Z_F
0.4	2	90	N	N	Y	Y	Y	Y	N	Y	Z_F
0.5	2	90	N	N	Y	Y	Y	Y	N	Y	Z_F
0.6	2	90	N	N	Y	Y	Y	Y	N	Y	Z_F
0.7	2	90	N	N	Y	Y	Y	Y	N	Y	Z_F
0.8	2	90	N	N	Y	Y	Y	Y	N	Y	Z_F
0.9	2	90	N	N	Y	Y	Y	Y	N	Y	Z_F
0.1	3	90	N	N	Y	Y	N	N	Y	Y	ZF_ρ
0.2	3	90	N	N	N	Y	N	N	Y	Y	ZF_ρ
0.3	3	90	N	N	Y	Y	N	N	Y	Y	ZF_ρ
0.4	3	90	N	N	Y	Y	Y	N	Y	Y	ZF_ρ
0.5	3	90	N	N	Y	Y	Y	N	Y	Y	ZF_ρ
0.6	3	90	N	N	Y	Y	N	N	Y	Y	ZF_ρ
0.7	3	90	N	N	Y	Y	N	N	Y	Y	F
0.8	3	90	N	N	Y	Y	Y	N	Y	Y	F
0.9	3	90	N	N	Y	Y	Y	Y	Y	Y	F
0.1	4	90	N	N	N	Y	N	N	Y	Y	ZF_ρ
0.2	4	90	N	N	N	Y	N	N	Y	Y	ZF_ρ
0.3	4	90	N	N	Y	Y	N	N	Y	Y	ZF_ρ
0.4	4	90	N	N	Y	Y	Y	Y	Y	Y	ZF_ρ
0.5	4	90	Y	N	Y	Y	Y	Y	Y	Y	ZF_ρ
0.6	4	90	Y	N	Y	Y	Y	Y	Y	Y	F
0.7	4	90	Y	Y	Y	Y	Y	Y	Y	Y	$Wald_F$
0.8	4	90	Y	Y	Y	Y	Y	Y	Y	Y	$Wald_F$
0.9	4	90	Y	N	Y	Y	Y	Y	Y	Y	$Wald_S$
0.1	5	90	N	N	N	Y	N	N	N	Y	ZF_ρ
0.2	5	90	N	N	Y	Y	N	N	Y	Y	ZF_ρ
0.3	5	90	N	N	Y	Y	N	N	Y	Y	ZF_ρ
0.4	5	90	N	N	Y	Y	Y	N	Y	Y	ZF_ρ
0.5	5	90	N	N	Y	Y	Y	N	Y	Y	ZF_ρ
0.6	5	90	N	N	Y	Y	Y	N	Y	Y	F
0.7	5	90	Y	N	Y	Y	Y	Y	Y	Y	F

0.8	5	90	Y	N	Y	Y	Y	Y	Y	Y	F
0.9	5	90	Y	N	Y	Y	Y	Y	Y	Y	$Wald_S$
0.1	6	90	N	N	N	Y	N	N	N	Y	ZF_ρ
0.2	6	90	N	N	Y	Y	N	N	Y	Y	ZF_ρ
0.3	6	90	N	N	Y	Y	N	N	Y	Y	ZF_ρ
0.4	6	90	N	N	Y	Y	Y	N	Y	Y	ZF_ρ
0.5	6	90	Y	N	Y	Y	Y	N	Y	Y	ZF_ρ
0.6	6	90	Y	N	Y	Y	Y	N	Y	Y	F
0.7	6	90	N	N	Y	Y	Y	N	Y	Y	F
0.8	6	90	N	N	Y	Y	Y	Y	Y	Y	F
0.9	6	90	Y	N	Y	Y	Y	N	Y	Y	F
0.1	7	90	N	N	N	Y	N	N	N	Y	ZF_ρ
0.2	7	90	N	N	N	Y	N	N	N	Y	ZF_ρ
0.3	7	90	N	N	N	Y	N	N	N	Y	ZF_ρ
0.4	7	90	N	N	Y	Y	N	N	Y	Y	ZF_ρ
0.5	7	90	N	N	Y	Y	Y	N	Y	Y	ZF_ρ
0.6	7	90	N	N	Y	Y	Y	N	Y	Y	F
0.7	7	90	N	N	Y	Y	Y	Y	Y	Y	F
0.8	7	90	N	N	Y	Y	Y	N	Y	Y	F
0.9	7	90	Y	N	Y	Y	Y	Y	Y	Y	F
0.1	8	90	N	N	N	Y	N	N	N	Y	ZF_ρ
0.2	8	90	N	N	N	Y	N	N	N	Y	ZF_ρ
0.3	8	90	N	N	N	Y	N	N	N	Y	ZF_ρ
0.4	8	90	N	N	N	Y	N	N	Y	Y	ZF_ρ
0.5	8	90	N	N	Y	Y	N	N	Y	Y	ZF_ρ
0.6	8	90	N	N	Y	Y	Y	N	Y	Y	F
0.7	8	90	N	N	Y	Y	Y	N	Y	Y	F
0.8	8	90	Y	N	Y	Y	Y	Y	Y	Y	F
0.9	8	90	Y	N	Y	Y	Y	Y	Y	Y	F
0.1	9	90	N	N	N	Y	N	N	N	Y	ZF_ρ
0.2	9	90	N	N	N	Y	N	N	N	Y	ZF_ρ
0.3	9	90	N	N	N	Y	N	N	N	Y	ZF_ρ
0.4	9	90	N	N	N	Y	N	N	N	Y	ZF_ρ
0.5	9	90	N	N	Y	Y	Y	N	Y	Y	ZF_ρ
0.6	9	90	N	N	Y	Y	Y	N	Y	Y	F
0.7	9	90	Y	N	Y	Y	Y	N	Y	Y	F
0.8	9	90	Y	N	Y	Y	Y	Y	Y	Y	F
0.9	9	90	Y	Y	Y	Y	Y	Y	Y	Y	$Wald_F$
0.1	10	90	N	N	N	Y	N	N	N	Y	ZF_ρ
0.2	10	90	N	N	N	Y	N	N	N	Y	ZF_ρ
0.3	10	90	N	N	N	Y	N	N	N	Y	ZF_ρ
0.4	10	90	N	N	N	Y	N	N	N	Y	ZF_ρ
0.5	10	90	N	N	N	Y	N	N	N	Y	ZF_ρ
0.6	10	90	N	N	Y	Y	N	N	Y	Y	F
0.7	10	90	Y	N	Y	Y	Y	Y	Y	Y	F
0.8	10	90	Y	N	Y	Y	Y	Y	Y	Y	F
0.9	10	90	Y	N	Y	Y	Y	Y	Y	Y	F
0.1	2	100	N	N	Y	Y	Y	Y	N	Y	Z_F
0.2	2	100	N	N	Y	Y	Y	Y	N	Y	Z_F
0.3	2	100	N	N	Y	Y	Y	Y	N	Y	Z_F

0.4	2	100	N	N	Y	Y	Y	Y	N	Y	Z_F
0.5	2	100	N	N	Y	Y	Y	Y	N	Y	Z_F
0.6	2	100	N	N	Y	Y	Y	Y	N	Y	Z_F
0.7	2	100	N	N	Y	Y	Y	Y	N	Y	Z_F
0.8	2	100	N	N	Y	Y	Y	Y	N	Y	Z_F
0.9	2	100	N	N	Y	Y	Y	Y	N	Y	Z_F
0.1	3	100	N	N	N	Y	N	N	Y	Y	ZF_ρ
0.2	3	100	N	N	N	Y	N	N	Y	Y	ZF_ρ
0.3	3	100	N	N	Y	Y	N	N	Y	Y	ZF_ρ
0.4	3	100	N	N	Y	Y	N	N	Y	Y	ZF_ρ
0.5	3	100	N	N	Y	Y	N	N	Y	N	F
0.6	3	100	N	N	Y	Y	N	N	Y	N	F
0.7	3	100	N	N	Y	Y	N	N	Y	Y	F
0.8	3	100	N	N	Y	Y	N	N	Y	Y	F
0.9	3	100	N	N	Y	Y	Y	N	Y	Y	F
0.1	4	100	N	N	N	Y	N	N	N	Y	ZF_ρ
0.2	4	100	N	N	N	Y	N	N	N	Y	ZF_ρ
0.3	4	100	N	N	N	Y	N	N	Y	Y	ZF_ρ
0.4	4	100	N	N	N	Y	N	N	Y	N	F
0.5	4	100	N	N	N	Y	N	N	Y	Y	ZF_ρ
0.6	4	100	N	N	N	Y	N	N	Y	Y	F
0.7	4	100	N	N	Y	Y	N	N	Y	N	F
0.8	4	100	N	N	Y	Y	N	N	Y	N	F
0.9	4	100	N	N	Y	Y	Y	N	Y	Y	F
0.1	5	100	N	N	N	Y	N	N	N	Y	ZF_ρ
0.2	5	100	N	N	N	Y	N	N	N	Y	ZF_ρ
0.3	5	100	N	N	N	Y	N	N	N	N	F
0.4	5	100	N	N	N	Y	N	N	N	N	F
0.5	5	100	N	N	N	Y	N	N	N	Y	ZF_ρ
0.6	5	100	N	N	Y	Y	N	N	Y	Y	F
0.7	5	100	N	N	Y	Y	N	N	Y	Y	F
0.8	5	100	N	N	Y	Y	Y	N	Y	Y	F
0.9	5	100	Y	N	Y	Y	Y	Y	Y	Y	$Wald_S$
0.1	6	100	N	N	N	Y	N	N	N	Y	ZF_ρ
0.2	6	100	N	N	N	Y	N	N	N	Y	ZF_ρ
0.3	6	100	N	N	N	Y	N	N	N	Y	ZF_ρ
0.4	6	100	N	N	Y	Y	N	N	N	Y	ZF_ρ
0.5	6	100	N	N	Y	Y	N	N	Y	Y	ZF_ρ
0.6	6	100	Y	N	Y	Y	N	N	Y	Y	F
0.7	6	100	Y	N	Y	Y	Y	N	Y	Y	F
0.8	6	100	Y	N	Y	Y	Y	N	Y	Y	F
0.9	6	100	Y	Y	Y	Y	Y	N	Y	Y	$Wald_F$
0.1	7	100	N	N	N	Y	N	N	N	Y	ZF_ρ
0.2	7	100	N	N	N	Y	N	N	N	Y	ZF_ρ
0.3	7	100	N	N	N	Y	N	N	N	Y	ZF_ρ
0.4	7	100	N	N	Y	Y	N	N	N	Y	ZF_ρ
0.5	7	100	N	N	Y	Y	N	N	Y	Y	ZF_ρ
0.6	7	100	Y	N	Y	Y	N	N	Y	Y	F
0.7	7	100	Y	N	Y	Y	Y	N	Y	Y	F
0.8	7	100	Y	N	Y	Y	Y	Y	Y	Y	F

0.9	7	100	Y	Y	Y	Y	Y	Y	Y	Y	$Wald_F$
0.1	8	100	N	N	N	Y	N	N	N	Y	ZF_ρ
0.2	8	100	N	N	N	Y	N	N	N	Y	ZF_ρ
0.3	8	100	N	N	N	Y	N	N	N	Y	ZF_ρ
0.4	8	100	N	N	N	Y	N	N	N	Y	ZF_ρ
0.5	8	100	N	N	Y	Y	N	N	Y	Y	ZF_ρ
0.6	8	100	N	N	Y	Y	N	N	Y	Y	F
0.7	8	100	N	N	N	Y	Y	Y	Y	Y	F
0.8	8	100	Y	N	Y	Y	Y	N	Y	Y	F
0.9	8	100	Y	N	Y	Y	Y	Y	Y	Y	F
0.1	9	100	N	N	N	Y	N	N	N	Y	ZF_ρ
0.2	9	100	N	N	N	Y	N	N	N	Y	ZF_ρ
0.3	9	100	N	N	N	Y	N	N	N	Y	ZF_ρ
0.4	9	100	N	N	N	Y	N	N	N	Y	ZF_ρ
0.5	9	100	N	N	Y	Y	N	N	Y	Y	ZF_ρ
0.6	9	100	N	N	Y	Y	N	N	Y	Y	F
0.7	9	100	Y	N	Y	Y	N	N	Y	Y	F
0.8	9	100	Y	Y	Y	Y	Y	N	Y	Y	F
0.9	9	100	Y	Y	Y	Y	Y	Y	Y	Y	$Wald_F$
0.1	10	100	N	N	N	Y	N	N	N	Y	ZF_ρ
0.2	10	100	N	N	N	Y	N	N	N	N	F
0.3	10	100	N	N	N	Y	N	N	N	Y	ZF_ρ
0.4	10	100	N	N	N	Y	N	N	N	Y	ZF_ρ
0.5	10	100	N	N	N	Y	N	N	N	Y	ZF_ρ
0.6	10	100	N	N	N	Y	N	N	N	Y	F
0.7	10	100	Y	N	Y	Y	N	N	Y	Y	F
0.8	10	100	Y	N	Y	Y	Y	Y	Y	Y	F
0.9	10	100	Y	Y	Y	Y	Y	Y	Y	Y	$Wald_F$

0.4 Supplementary Table 4

The table gives a summary of the number of participants when the confidence interval methods show acceptable coverage for different values of ρ and k.

ρ	k	$Wald_S$	$Wald_F$	$Wald_{Ze}$	F	Z_S	Z_F	Z_{Ze}	ZF_ρ
0.1	3			≥50<br>≠100	≥20	≥70<br>≠90,100	≥80<br>≠90,100	≥20	≥30
0.1	4			≥80<br>≠90,100	≥20	≥80<br>≠90,100		≥50<br>≠60,70,100	≥30
0.1	5			≥80<br>≠90,100	≥20			≥80<br>≠90,100	≥30<br>≠40,60
0.1	6			≥80<br>≠90,100	≥20			≥80<br>≠90,100	≥40
0.1	7				≥20				≥40<br>≠50
0.1	8				≥20				≥50<br>≠60
0.1	9				≥20				≥50
0.1	10				≥20				≥40
0.2	2			≥30	≥20	≥40	≥40		≥40
0.2	3			≥40<br>≠90,100	≥20	≥70<br>≠90,100	≥70<br>≠90,100	≥20	≥30
0.2	4			≥70<br>≠90,100	≥20			≥50<br>≠60,100	≥30
0.2	5			≥80<br>≠100	≥20	≥80<br>≠90,100		≥80<br>≠100	≥30<br>≠40,60,70
0.2	6			≥80<br>≠100	≥20	≥80<br>≠90,100		≥80<br>≠100	≥30
0.2	7			≥80<br>≠90,100	≥20			≥80<br>≠90,100	≥40<br>≠50,60
0.2	8				≥20				≥50<br>≠60

ρ	k	$Wald_S$	$Wald_F$	$Wald_{Ze}$	F	Z_S	Z_F	Z_{Ze}	ZF_ρ
0.2	9				≥20				≥40 ≠60
0.2	10				≥20				≥40 ≠100
0.3	2			≥30	≥20	≥40	≥40 ≠50		≥40
0.3	3	≥80 ≠90,100		≥30	≥20	≥70 ≠90,100	≥70 ≠90,100	≥20	≥30
0.3	4			≥70 ≠100	≥20			≥50	≥30
0.3	5			≥80 ≠100	≥20	≥80 ≠90,100		≥80 ≠100	≥30 ≠60,100
0.3	6			≥80 ≠100	≥20	≥80 ≠90,100		≥80 ≠100	≥30
0.3	7				≥20				≥40 ≠60
0.3	8				≥20				≥50 ≠60
0.3	9				≥20				≥40 ≠60
0.3	10				≥20				≥40 ≠60
0.4	2			≥30	≥20	≥20	≥40		≥20
0.4	3			≥30	≥20	≥70 ≠100	≥80 ≠90,100	≥20	≥30
0.4	4			≥50 ≠60,100	≥20	≥80 ≠100	≥90 ≠100	≥30 ≠40	≥30 ≠40,100

ρ	k	$Wald_S$	$Wald_F$	$Wald_{Ze}$	F	Z_S	Z_F	Z_{Ze}	ZF_ρ
0.4	5			≥80 ≠100	≥20	≥80 ≠100		≥50 ≠60,100	≥30 ≠100
0.4	6	≥80 ≠90,100		≥80	≥20	≥80 ≠100		≥70 ≠100	≥20 ≠30
0.4	7			≥90	≥20			≥90 ≠100	≥40 ≠60
0.4	8				≥20			≥90 ≠100	≥40
0.4	9				≥20				≥40 ≠60
0.4	10				≥20				≥40
0.5	2			≥30	≥20	≥20	≥40		≥20
0.5	3			≥30	≥20	≥70 ≠100	≥70 ≠90,100	≥40	≥30 ≠100
0.5	4	≥90 ≠100		≥40 ≠100	≥20	≥80 ≠100	≥90 ≠100	≥20	≥30
0.5	5			≥80 ≠100	≥20	≥80 ≠100		≥40 ≠100	≥30
0.5	6	≥90 ≠100		≥80	≥20	≥80 ≠100	≥80 ≠90,100	≥40 ≠50	≥20 ≠30
0.5	7			≥80	≥20	≥90 ≠100		≥80	≥40 ≠50,60
0.5	8			≥80	≥20			≥80	≥40
0.5	9			≥90	≥20	≥90 ≠100		≥90	≥40 ≠50,60
0.5	10			≥70 ≠80,90,100	≥20				≥40
0.6	2			≥20	≥20	≥20	≥40		≥20

ρ	k	$Wald_S$	$Wald_F$	$Wald_{Ze}$	F	Z_S	Z_F	Z_{Ze}	ZF_ρ
0.6	3	≥80<br>≠90,100	≥80<br>≠90,100	≥20	≥20	≥50<br>≠90,100	≥60<br>≠90,100	≥50	≥30<br>≠100
0.6	4	≥90<br>≠100		≥20<br>≠100	≥20	≥70<br>≠100	≥90<br>≠100	≥20	≥30
0.6	5	≥80<br>≠90,100		≥40<br>≠60	≥20	≥80<br>≠100		≥40	≥40
0.6	6	≥90		≥40<br>≠50,60	≥20	≥80<br>≠100		≥40	≥40
0.6	7	≥80<br>≠90		≥80	≥20	≥90<br>≠100		≥70	≥40<br>≠60
0.6	8			≥70	≥20	≥80<br>≠100		≥80	≥40
0.6	9			≥70	≥20	≥90<br>≠100		≥80	≥40<br>≠60
0.6	10			≥90<br>≠100	≥20			≥90<br>≠100	≥40
0.7	2			≥20	≥20	≥20	≥40		≥20
0.7	3	≥80<br>≠90,100	≥80<br>≠90,100	≥20	≥20	≥40<br>≠90,100	≥60<br>≠90,100	≥50	≥30
0.7	4	≥80<br>≠100	≥90<br>≠100	≥20	≥20	≥60<br>≠80,100	≥90<br>≠100	≥20	≥40<br>≠100
0.7	5	≥70<br>≠100		≥20	≥20	≥80<br>≠100	≥90<br>≠100	≥20	≥40
0.7	6	≥80<br>≠90		≥40<br>≠50	≥20	≥80		≥40	≥40
0.7	7	≥80<br>≠90		≥40	≥20	≥80	≥90<br>≠100	≥40	≥40<br>≠60

ρ	k	$Wald_S$	$Wald_F$	$Wald_{Ze}$	F	Z_S	Z_F	Z_{Ze}	ZF_ρ
0.7	8	≥50<br>≠60,70,80,90,100		≥40<br>≠60,100	≥20	≥80	≥100	≥50<br>≠60	≥40
0.7	9	≥40<br>≠60,80		≥40<br>≠60	≥20	≥90<br>≠100		≥70	≥40<br>≠60
0.7	10	≥40<br>≠50,60,70,80		≥40<br>≠70	≥20	≥90<br>≠100	≥90<br>≠100	≥70	≥40
0.8	2			≥20	≥20	≥20	≥40		≥20
0.8	3	≥70<br>≠90,100	≥80<br>≠90,100	≥20	≥20	≥30<br>≠100	≥50<br>≠90,100	≥60	≥30
0.8	4	≥60<br>≠100	≥90<br>≠100	≥20	≥20	≥40<br>≠100	≥70<br>≠80,100	≥20	≥40<br>≠100
0.8	5	≥40<br>≠60,100	≥70<br>≠90,100	≥20	≥20	≥70	≥80<br>≠100	≥20	≥40
0.8	6	≥40<br>≠50,90	≥80<br>≠90,100	≥20	≥20	≥40<br>≠50,60	≥90<br>≠100	≥20	≥40
0.8	7	≥40<br>≠50,60,90		≥20	≥20	≥70	≥80<br>≠90	≥30	≥40<br>≠60
0.8	8	≥40<br>≠60	≥40<br>≠50,60,80,90,100	≥30	≥20	≥80	≥90<br>≠100	≥40	≥40
0.8	9	≥40<br>≠60	≥40<br>≠50,60,70,80,90	≥30	≥20	≥70<br>≠80	≥90<br>≠100	≥40	≥40
0.8	10	≥40<br>≠60,70	≥40<br>≠50,60,70,80,90,100	≥30	≥20	≥70	≥90	≥40	≥40
0.9	2			≥20	≥20	≥20	≥40		≥20
0.9	3	≥50<br>≠60,90,100	≥80<br>≠90,100	≥20	≥20	≥40	≥50<br>≠100	≥60	≥30
0.9	4	≥40<br>≠100	≥50<br>≠90,100	≥20	≥20	≥40	≥80<br>≠100	≥30	≥40

ρ	k	$Wald_S$	$Wald_F$	$Wald_{Ze}$	F	Z_S	Z_F	Z_{Ze}	ZF_ρ
0.9	5	≥30	≥40<br>≠60,90,100	≥20	≥20	≥40<br>≠60	≥80	≥20	≥40
0.9	6	≥30	≥40<br>≠60,90	≥20	≥20	≥40	≥60<br>≠70,90,100	≥20	≥40
0.9	7	≥30	≥40<br>≠60,90	≥20	≥20	≥40<br>≠50	≥80	≥20	≥40
0.9	8	≥20	≥40<br>≠90,100	≥20	≥20	≥50<br>≠60	≥90	≥20	≥40
0.9	9	≥20	≥40<br>≠60,70	≥20	≥20	≥50<br>≠60	≥90	≥40	≥40
0.9	10	≥20	≥40<br>≠60,90	≥20	≥20	≥50<br>≠60	≥90	≥40	≥40
